# Aerobic C−N Bond Formation through Enzymatic Nitroso‐Ene‐Type Reactions**

**DOI:** 10.1002/anie.202213671

**Published:** 2023-01-09

**Authors:** Christina Jäger, Mona Haase, Katja Koschorreck, Vlada B. Urlacher, Jan Deska

**Affiliations:** ^1^ University of Helsinki Department of Chemistry A.I. Virtasen aukio 1 00560 Helsinki Finland; ^2^ Aalto University Department of Chemistry Kemistintie 1 02150 Espoo Finland; ^3^ Heinrich-Heine-Universität Düsseldorf Universitätsstr. 1 40225 Düsseldorf Germany

**Keywords:** Amination, Biocatalysis, Cyclization, Ene Reaction, Heterocycles

## Abstract

The biocatalytic oxidation of acylated hydroxylamines enables the direct and selective introduction of nitrogen functionalities by activation of allylic C−H bonds. Utilizing either laccases or an oxidase/peroxidase couple for the formal dehydrogenation of *N*‐hydroxycarbamates and hydroxamic acids with air as the terminal oxidant, acylnitroso species are generated under particularly mild aqueous conditions. The reactive intermediates undergo C−N bond formation through an ene‐type mechanism and provide high yields both in intramolecular and intermolecular enzymatic aminations. Investigations on different pathways of the two biocatalytic systems and labelling studies provide more insight into this unprecedented promiscuity of classical oxidoreductases as catalysts for nitroso‐based transformations.

## Graphical abstract

In an exciting case of catalytic promiscuity, peroxidases and laccases participate in C−N bond‐forming reactions through the generation of reactive nitroso intermediates from acylated hydroxylamines. The formal allylic C−H bond activation proceeds with air as the terminal oxidant and provides high yields both in intramolecular and intermolecular amination reactions through this unprecedented biocatalytic nitroso‐ene‐type reaction pathway.

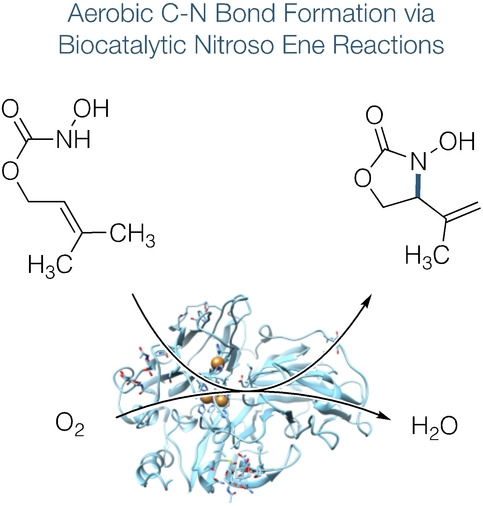

## Introduction

**Scheme 1:**
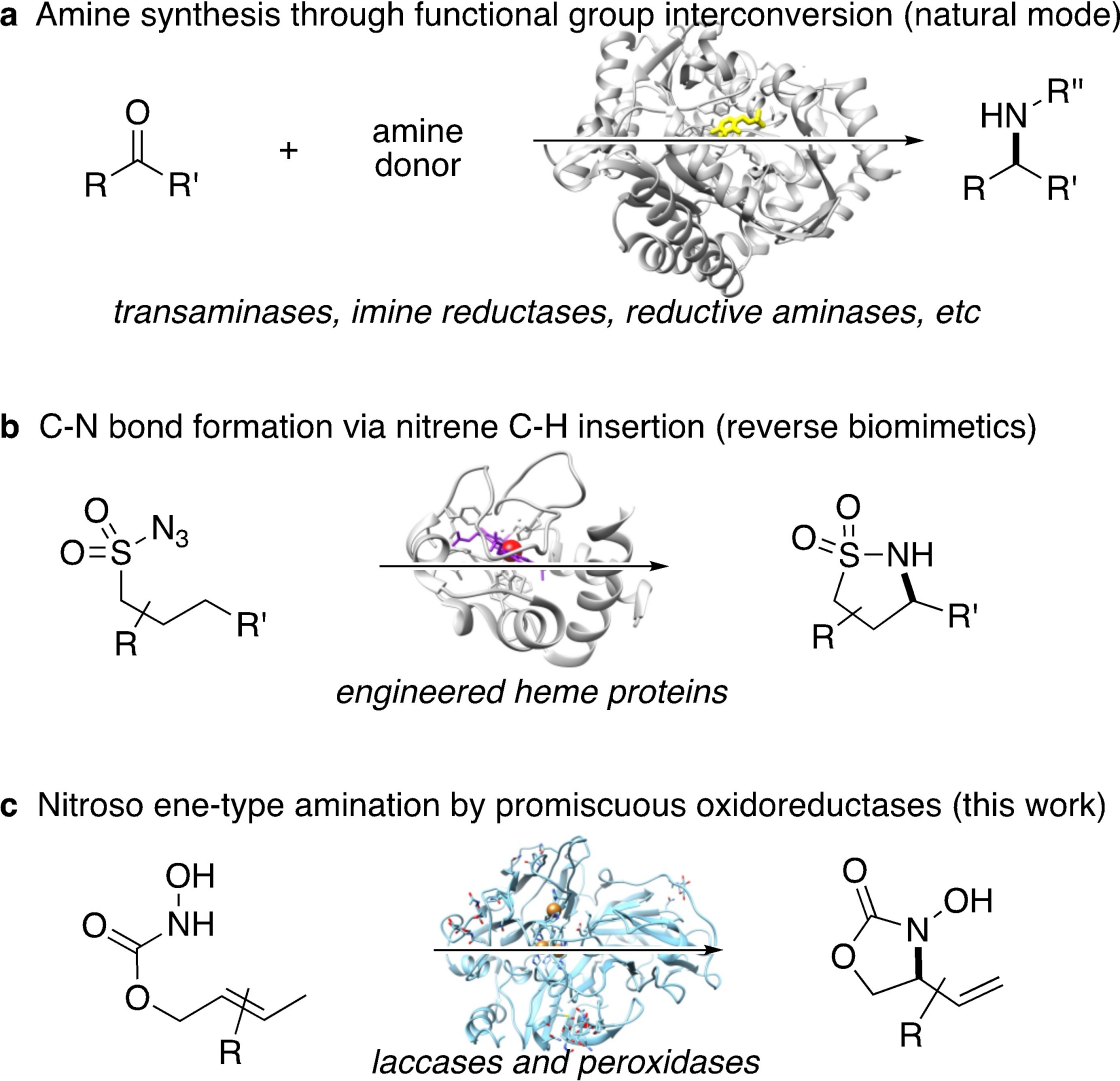
Approaches for C−N bond formation mediated by enzyme catalysts: a) established biocatalytic amination by transferases and oxidoreductases; b) designer biocatalysts enabling nitrene insertion chemistry; c) ene‐type functionalization by laccases and peroxidases.

Nature's repertoire to introduce nitrogen moieties into molecular frameworks is generally limited to direct functional group interconversions. In addition to the well‐developed amide‐forming enzymes, the toolbox of synthetically valuable biocatalytic methods has been expanded steadily in the past years through a number of nitrogen‐fixation strategies based on reductive aminations and additions catalyzed by transaminases, imine reductases, and amine dehydrogenases, to name a few.^[1]^ In a complementary approach mimicking chemistry by biological means, most recently the biocatalytic imitation of non‐natural but synthetically relevant reactions was moved into the spotlight,^[2]^ and in particular, nitrene insertion‐based transformations utilizing engineered heme proteins introduced whole new pathways for the biocatalytic C−N bond formation (Scheme 1).^[3]^

As a yet untapped template from the world of traditional synthesis, another attractive alternative approach for selective C−N bond formations engages reactive nitroso species as electrophiles with allylic functionalities. First explored in the 1960s for the synthesis of allylamines using nitrosobenzene,^[4]^ nitroso ene reactions, and the related nitroso‐Diels‐Alder cycloadditions, are nowadays found in a wide variety of synthetic strategies towards natural products and pharmaceuticals.^[5]^ Due to their high reactivity, nitroso reagents are most commonly created in situ through oxidation of amines or hydroxylamines, or the reduction of nitro moieties.^[6]^ Among the different derivatives, particularly acylnitroso species have been proven as versatile reaction partners, as these electrophiles offer a good compromise between reactivity and selectivity.^[7]^ As the C−N bond formation through nitroso ene reactions proceeds with specificity on allylic fragments even on more functionalized molecules, a variety of different methodologies for the synthesis and trapping of acylnitroso species have been developed over the years. Common approaches include the thermal dissociation,^[8]^ photooxidations,^[9]^ or the application of stoichiometric amounts of oxidizing agents, such as peroxides,^[10]^ diacetoxyiodobenzene,^[11]^ *N*‐methylmorpholine *N*‐oxide (NMO),^[12]^ and periodates.^[13]^ Logically, those methods were subsequently supplemented by a variety of catalytic versions employing transition‐metal complexes as mediators for the oxidative or reductive generation of the nitroso electrophiles, covering protocols based on iridium, ruthenium, rhodium, molybdenum and palladium.^[14]^ Particularly mild conditions have been achieved by rather simple catalytic systems utilizing copper^[15]^ or iron^[16]^ catalysts in combination with oxygen or hydrogen peroxide as terminal oxidants, which provide effective C−N bond formation both in intra‐ and intermolecular reactions. Although single examples of catalytic enantioselective methods have been reported for nitroso‐Diels‐Alder cycloadditions,^[17]^ the application of the nitroso ene reaction for the synthesis of optically active amines has so far been limited to auxiliary‐controlled diastereoselective transformations.[^15c^, ^18^]

In our endeavor to discover new enzymatic tools that are inspired by traditional organic chemistry, we envisaged that oxidative biocatalysts could act as mediators for this attractive C−N‐coupling reaction. While ensuring mild conditions and benign oxidants, the potential enzyme catalysts would need to provide a protective environment for the reactive nitroso electrophiles, which, if successful, could also serve as starting point for protein engineering efforts to develop enantioselective biocatalyst variants.

## Results and Discussion

With both copper‐ and iron‐based homogeneous systems described in the literature, our investigation on the biocatalytic nitroso ene reaction started with broad screening of a series of redox‐active metalloproteins featuring either of these two elements as an active site motif. As one of the most prominent copper‐dependent enzyme classes with precedent as a tool in organic synthesis and precedent in the formation of N=O species in oxoammonium‐mediated dehydrogenation reactions,^[19]^ oxygen‐activating laccases were initially taken into the focus of our study. We commenced our screening for this non‐natural activity with a small set of three fungal and two bacterial laccases. Among the fungal blue‐copper enzymes, laccases from *Myceliophthora thermophila* and *Moniliophthora roreri* failed to show substantial nitroso‐ene reactivity (Table 1, entries 1 and 2), and only Lccβ from *Trametes versicolor* exhibited significant selectivity in the aerobic activation of *N*‐hydroxycarbamate **1 a**, providing high yields of the oxazolidinone **2 a** (Table 1, entry 3). Likewise, while the *Streptomyces*‐derived laccase Ssv1 could not convert model substrate **1 a**, CotA from *Bacillus licheniformis* offered highly effective aerobic C−N bond formation with 74 % yield of the heterocycle **2 a** (Table 1, entries 4 and 5). The discrepancy between conversion and yields indicates minor selectivity issues and it appears that subsequent oxidation and decomposition of the *N*‐hydroxy oxazolidinone **2 a** may hamper the process. As CotA features a good tolerance to neutral or even slightly basic reaction conditions,^[20]^ the bacterial laccase was chosen for further optimization and general method development (Supporting Information Figures S2 and S3). Especially at lower reaction temperatures, the selectivity of CotA could be further improved, reaching 83 % yield at 5 °C after 26 h (Supporting Information Figure S4). Although the solubility of **1 a** in the phosphate buffer was not an issue, cosolvent solutions were taken into consideration, as structural variations on related *N*‐hydroxy carbamates and hydroxamic acids could affect substrate lipophilicity unfavorably. Here, concentrations of 10 vol % of water‐miscible cosolvents, such as dioxane, dimethylsulfoxide and acetonitrile, were well tolerated (Table 1, entry 6 and Supporting Information Figure S5).

**Table 1:** Enzyme screening for the oxidative cyclization of **1 a**.

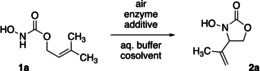					
Entry	Enzyme	Additive	Cosolvent^[a]^	Time [h]	Yield [%]^[b,c]^
1	laccase Mtl (*M. thermophila*)	–	–	6	9 (n.a.^[d]^)
2	laccase Mrl2 (*M. roreri*)	–	–	6	4 (n.a.^[d]^)
3	laccase Lccβ (*T. versicolor*)	–	–	6	**78 (100)**
4	laccase Ssv1 (*S. sviceus*)	–	–	6	3 (n.a.^[d]^)
5	laccase CotA (*B. licheniformis*)	–	–	7	**74 (100)**
6	laccase CotA (*B. licheniformis*)	–	dioxane	7	69 (100)
7	peroxygenase (*A. aegerita*)	d‐Glc, GOx (*A. niger*)	–	48	0 (0)
8	lactoperoxidase (bovine)	d‐Glc, GOx (*A. niger*)	–	72	5 (n.a.^[d]^)
9	chloroperoxidase (*C. fumago*)	d‐Glc, GOx (*A. niger*)	–	29	64 (100)
10	peroxidase (from horseradish)	d‐Glc, GOx (*A. niger*)	–	2	**97 (100)**
11	peroxidase (from horseradish)	d‐Glc, GOx (*A. niger*)	EtOAc	2	96 (100)
12	peroxidase (from horseradish)	d‐Glc, GOx (*A. niger*)	dioxane	2	94 (100)
13	none	d‐Glc, GOx (*A. niger*)	–	48	0

Reaction conditions, representative for laccases: **1 a** (10 mm), laccase CotA (K316N/D500G, 1 U, 2.7 nmol), phosphate buffer (pH 7.0, 100 mm, 7 mL), 25 °C; representative for peroxidases: **1 a** (10 mm), d‐glucose (50 mm), glucose oxidase (70 U, 0.5 nmol), horseradish peroxidase (70 U, 1.5 nmol), phosphate buffer (pH 7.0, 100 mm, 7 mL), 25 °C. For other entries, enzyme amounts were normalized to their respective units relative to the benchmark assay (see the Supporting Information). [a] 10 vol %. [b] Yields of isolated **2 a**. [c] Conversion in parenthesis. [d] Full conversion was reached after a longer incubation time with higher enzyme loadings (see Supporting Information Figure S6).

Next, we turned our attention to a bi‐enzymatic system that was previously employed by us successfully in the aerobic oxidative transformation of furans and allenes.^[21]^ Combining glucose oxidase (GOx) as oxygen‐activating biocatalyst with peroxidases or peroxygenases to mediate the oxidation of the substrate, we imagined that also these biocatalysts could generally generate the reactive acylnitroso species in situ. In the presence of glucose, GOx and air, little to no product formation was observed with peroxygenase and lactoperoxidase (Table 1, entries 7 and 8). In contrast, with chloroperoxidase from *C. fumago*, full conversion of **1 a** was observed within 29 h. A yield of 64 % of **2 a**, however, indicated that also with this biocatalyst, similar to the most effective laccases, overoxidation remained a problem (Table 1, entry 9). Gratifyingly, supplementing horseradish peroxidase (HRP) resulted in not only fast conversion but also an excellent isolated yield of 97 % (Table 1, entry 10). The GOx/HRP system also proved to be very robust, and neither the addition of water‐miscible dioxane nor a two‐phase solution featuring ethyl acetate caused significant deterioration of reactivity or selectivity (Table 1, entries 11 and 12), making this bi‐enzymatic nitroso‐ene protocol a promising candidate for practical synthetic applications. In the absence of added peroxidase, no reaction took place, ruling out any action by the glucose oxidase additive itself (Table 1, entry 13). Likewise, reactions in which the peroxidase was replaced with only the hemin cofactor did not show any cyclization activity.

**Scheme 2:**
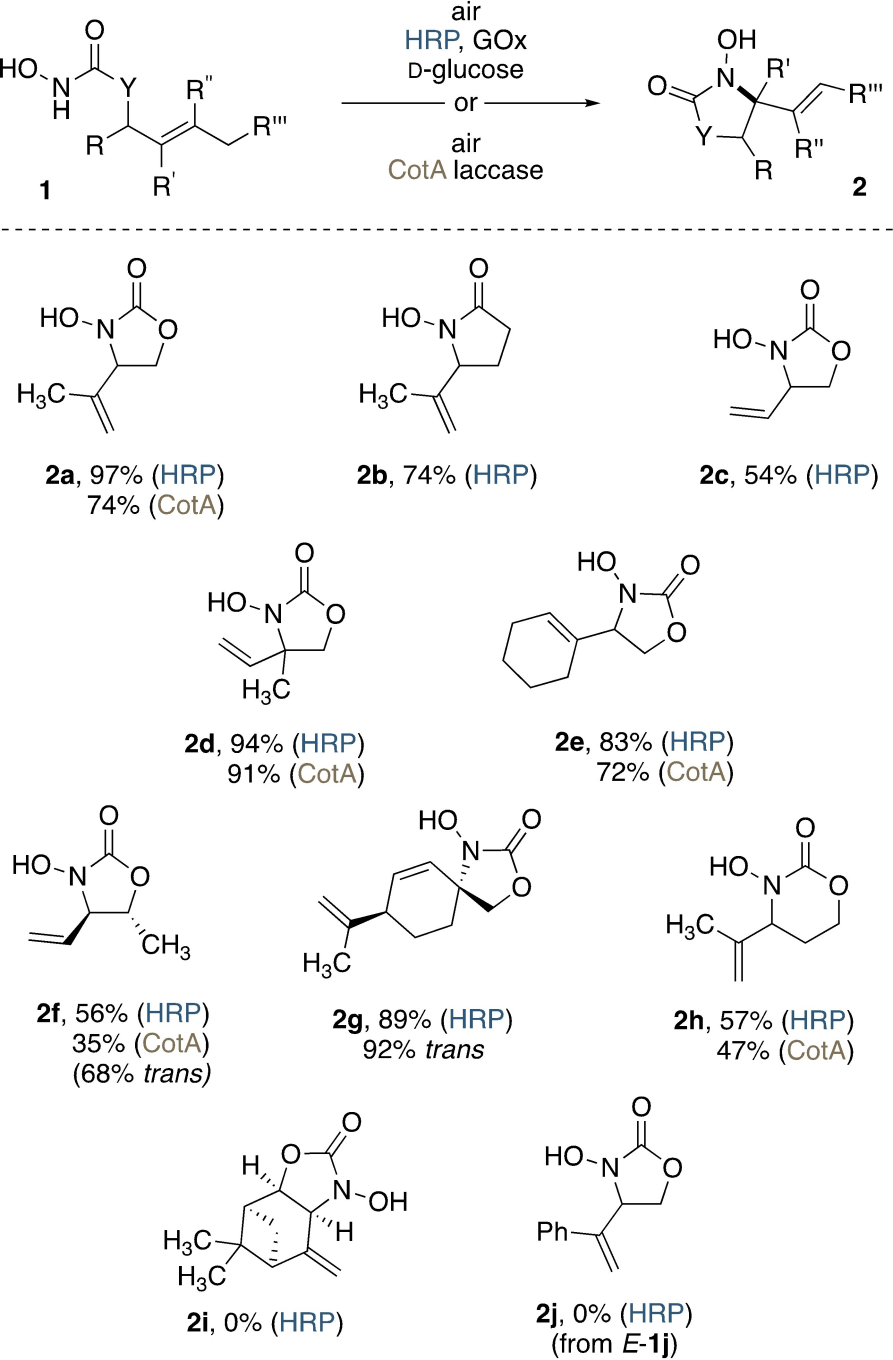
Scope of the biocatalytic aerobic nitroso ene cyclization. For total turnover numbers and standard deviations, see Supporting Information Table S2.

The peroxidase‐based cyclization protocol was subsequently examined regarding the tolerated substrate scope and structural limitations of the methodology, while at the same time, some substrates were also cyclized with CotA to allow for better comparison between the methods (Scheme 2). Changing from the *N*‐hydroxy carbamate motif to a hydroxamic acid in **1 b**, good reactivity could be maintained, and the *N*‐hydroxy lactam **2 b** was obtained in 77 % yield. Similarly, variation of the substitution pattern around the olefinic double bond was generally tolerated, as indicated by moderate to very high yields of products **2 c**–**2 e**. All reactions proceeded well at room temperature, which poses a stark contrast to the original chemical template that required up to 100 °C to achieve comparable yields.^[16]^ Utilizing chiral *N*‐hydroxy carbamates, good to excellent induced diastereoselectivity was observed, and the spirocyclic product **2 g** was isolated in 97 % yield as 92 : 8 mixture of diastereomers. Additionally, both methods also rendered the six‐membered *N*,*O*‐heterocycle **2 h** in moderate yields, suggesting possible applications beyond the oxazolidinone motif. In contrast, no conversion was observed for verbenol‐derived **1 i** or 3‐methylcinnamyl substrate *E*‐**1 j**. Overall, the HRP/GOx system delivered slightly higher yields as compared to the laccase, yet in all tested cases also the copper enzyme offered effective catalysis in this non‐natural transformation. With maximum turnover numbers of 43 500 (HRP) and 12 100 (CotA), both enzymes show good catalytic efficacy (Supporting Information Table S2), especially considering that the nitroso‐ene‐type reaction is far from the native reaction scope of either of the enzymes. The HRP‐mediated process also appeared to be well scalable, and at a seven‐fold increase in the reaction scale for the conversion of **1 a**, *N*‐hydroxyoxazolidinone **2 a** was still obtained in 92 % yield.

**Scheme 3:**
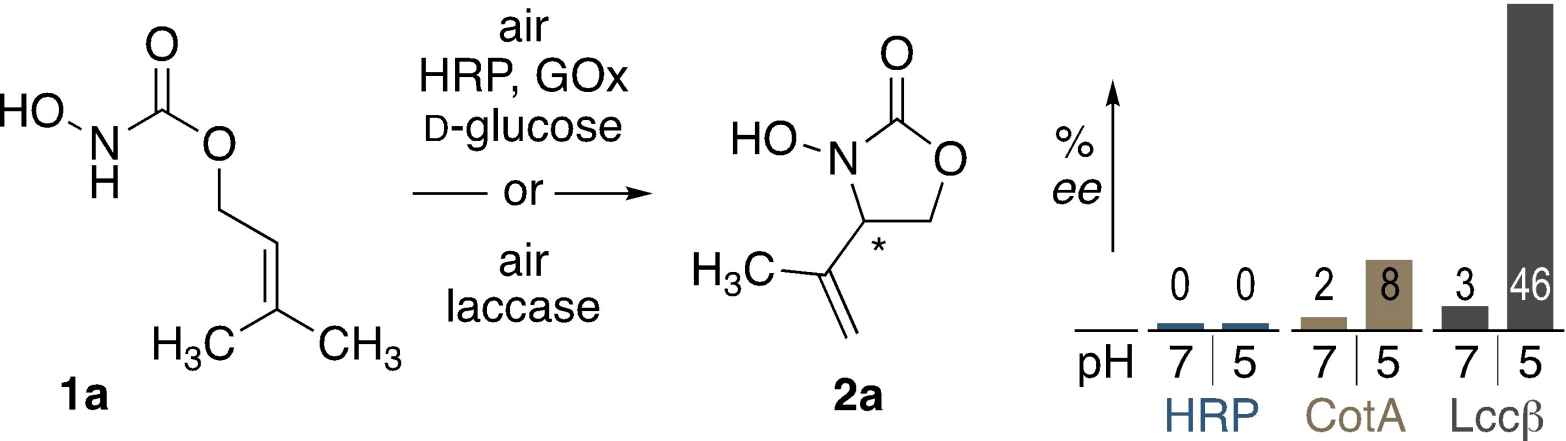
Enantioselectivity in biocatalytic nitroso ene reactions.

Apparently, the conversion of **1 a** into **2 a** includes formation of a stereogenic center, and naturally, enantioselectivity is almost taken for granted in biocatalysis. Strong stereochemical induction, however, would require a distinct binding site for the reactive nitroso intermediate, and in our study, all peroxidase‐catalyzed reactions yielded racemic **2 a**. In contrast, the architecture of the laccase may indeed provide a certain kind of substrate binding motif, as moderate enantiomeric excess was achieved with laccases, reaching up to 46 % *ee* (±4 %) with Lccβ from *T. versicolor* (Supporting Information Figures S7 and S18). Gratifyingly, changing to hydroxycarbamate **1 e**, the selectivity could be increased, and the corresponding cyclization product **2 e** was obtained with 83 % *ee* (Supporting Information Figure S19). So far, this strong stereochemical induction could only be observed under slightly acidic conditions, under which unfortunately also product degradation is facilitated (Scheme 3), leading to only mediocre yields (i.e. 30 % for **2 e**) at full conversion. Nevertheless, this observation represents a very rare case of enantiocontrol during laccase catalysis.^[22]^ The pH dependence on (dia)stereoselectivity in these oxidase‐mediated processes is not unheard of, and either changes in the protonation state of active‐site residues or the modulation of redox potential may play a role here.^[23]^ Thus, further investigation based on protein engineering with these blue‐copper enzymes, as stereoselective nitroso ene catalysts poses an attractive line of research.

**Scheme 4:**
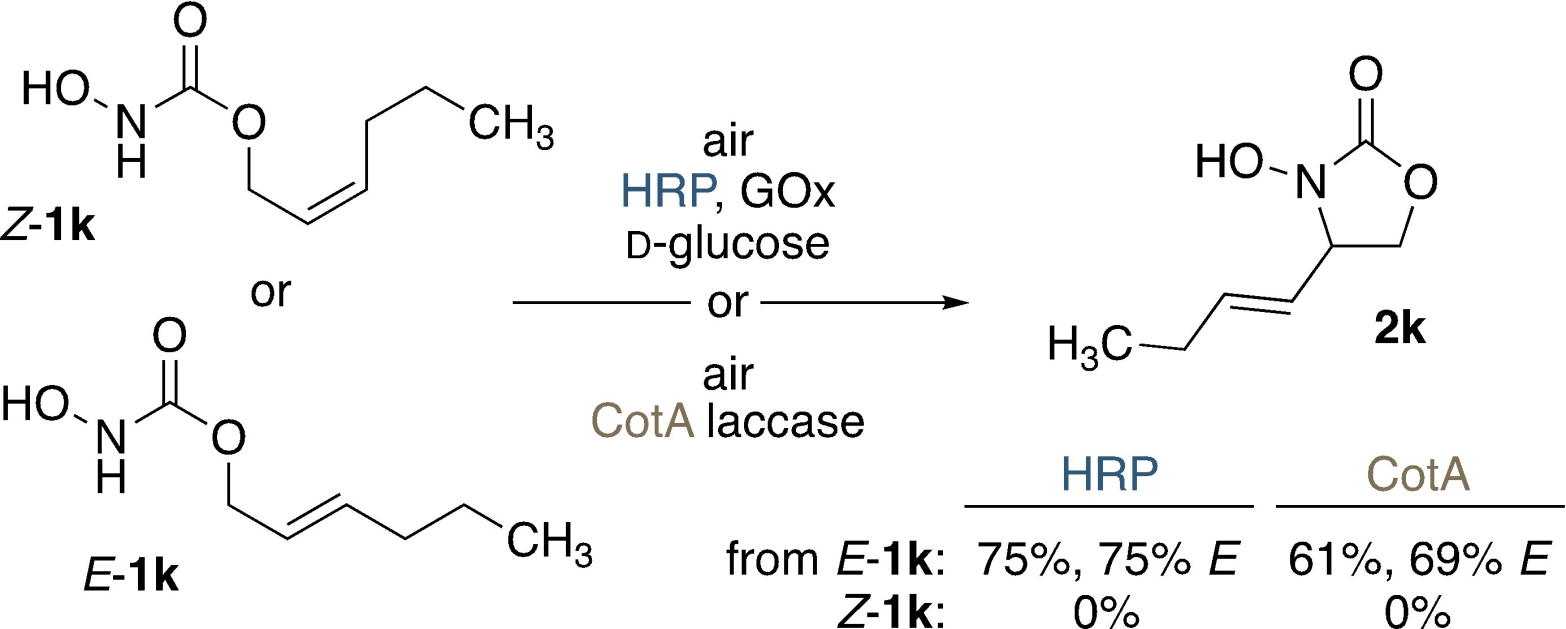
*E*/*Z*‐discrimination in the biocatalytic aerobic activation.

To get a clearer picture of the potential geometrical bias of the HRP‐ and CotA‐catalyzed cyclizations, the two isomeric 2‐hexenyl *N*‐hydroxycarbamates *E*‐**1 k** and *Z*‐**1 k** were treated under identical conditions (Scheme 4). The *E*‐configured isomer delivered the expected oxazolidinone **2 k** in good yields of 66 % and 67 %. With a rotationally flexible CH_2_ unit in the allylic position, the product was obtained as a 3 : 1 *E*/*Z* mixture. In contrast, no cyclization product could be detected with the stereoisomeric substrate. Instead, *Z*‐**1 k** was quantitatively decomposed with either of the biocatalytic systems, giving rise to the corresponding allylic alcohol, indicating the formation, hydrolysis and decarboxylation of the nitroso intermediate (Supporting Information Figure S17).

**Scheme 5:**
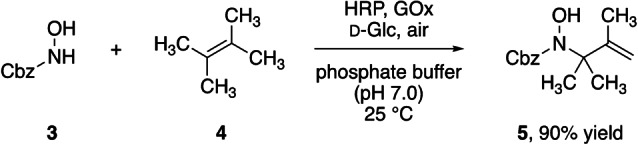
Enzymatic intermolecular nitroso ene reaction.

This observation underlines that the half‐life of acylnitroso species in the aqueous reaction medium is most likely limited, as they are generally characterized as short‐lived electrophiles. In a recent study by Goldstein and co‐workers,^[24]^ the rates of hydrolysis of nitroso carbonyl compounds prepared in situ, and reactions with other nucleophiles, were investigated and half‐lives of a few minutes were observed. In the biocatalytic cyclizations, the intramolecular nature and a potential protective function of the proteins may explain the good to excellent yields. When moving to an intermolecular system, however, the exposure of the reactive intermediate is unavoidable. Not surprisingly, initial attempts to conduct a bimolecular coupling between *N*‐hydroxycarbamate **3** and tetramethylethylene (**4**) in equimolar amounts under the previously optimized conditions with the oxidase/peroxidase couple resulted in full conversion in 2 h, but with a mediocre yield of only 30 %. To our delight, simply swapping the organic cosolvent from ethyl acetate to tetramethylethylene itself resulted in identical kinetics and the disappearance of **3** within 2 h. In this case, however, the C−N‐coupling product **5** was isolated in an excellent yield of 90 % (Scheme 5 and Supporting Information Table S1). For comparison, due to the lower tolerance of laccases to lipophilic cosolvents, the CotA‐catalyzed intermolecular coupling yielded only 53 % of **5**. Though practical for some olefinic reaction partners, the use of excess reactants is certainly not the most elegant approach. Thus, it will be necessary to further adjust the reaction conditions for this intermolecular nitroso ene reaction, either through reaction medium engineering,^[21c]^ or through modulation of the reactivity by variation of the electronics of the enophile.^[7]^

**Scheme 6:**
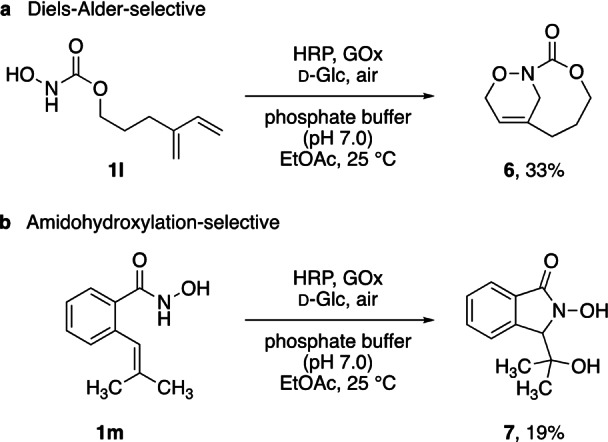
Cycloaddition‐ and amidohydroxylation‐selective conversions through HRP/GOx‐mediated aerobic oxidation.

With a highly effective biocatalytic protocol in hand to prepare and transform acylnitroso species in situ under mild aerobic conditions, the extension to related reactions as probe for the synthetic potential of this enzymatic tool appeared to be a logical next step. With proven activity in both intra‐ and intermolecular ene‐type reactions, we assumed that the peroxidase could likewise act as catalyst in nitroso‐Diels‐Alder reactions. Utilizing *N*‐hydroxycarbamate **1 l** featuring a diene moiety and a geometry that would rule out competing ene reactivities, our established method indeed translated well into an intramolecular [4+2] cycloaddition, reaching full conversion of the substrate and yielding bicycle **6** as the major product (Scheme 6a). The existence of a cycloaddition pathway further strengthens the suggested overall activation mode, where acylnitroso species are formed by dehydrogenation of the hydroxylamine derivatives.

More surprisingly, the peroxidase‐mediated oxidation of the benzoate‐derived hydroxamic acid **1 m** resulted in a slightly different reaction outcome, providing amidohydroxylated species **7** selectively as the sole product, alongside unreacted starting material (Scheme 6b). Even though similar N,O‐difunctionalizations of olefins are not unknown,^[25]^ to the best of our knowledge, this bifurcation has so far not been observed in previous nitroso ene studies and could indicate an interesting additional avenue for future investigations. Notably, none of the other substrates tested in this study led to similar amidohydroxylation products. While the current yield of 20 % would certainly require more optimization efforts to present the reaction as serious synthetic method, this deviation from the expected product outcome might help to shed some light on potential pathways of the nitroso ene reaction under the herein described aqueous enzymatic conditions (see below).

While the nitroso‐Diels‐Alder and nitroso ene reactions are identical in how the reactive R‐NO species are formed, the ene‐type reaction is believed to follow a stepwise reaction pathway as opposed to the concerted [4+2] cycloaddition of the Diels‐Alder mechanism. The underlying process of these kinds of ene reactions is still under debate, and several feasible mechanisms have been proposed over the years. Most plausible pathways, based on experimental and/or computational studies, involve zwitterionic or diradical intermediates, concerted pathways, as well as intermediary aziridine *N*‐oxides (ANOs).[^26^, ^27^, ^28^, ^29^, ^30^] Although not unchallenged, the current established view assumes a combination of hypotheses with a polarized diradical pathway that does not rule out the ANO as an “innocent bystander”.[^27^, ^30^] However, these mechanistic studies mostly refer to the intermolecular ene reaction of nitroso compounds, and are likely not fully transferable to our system owing to the unusual aqueous reaction environment.

**Scheme 7:**
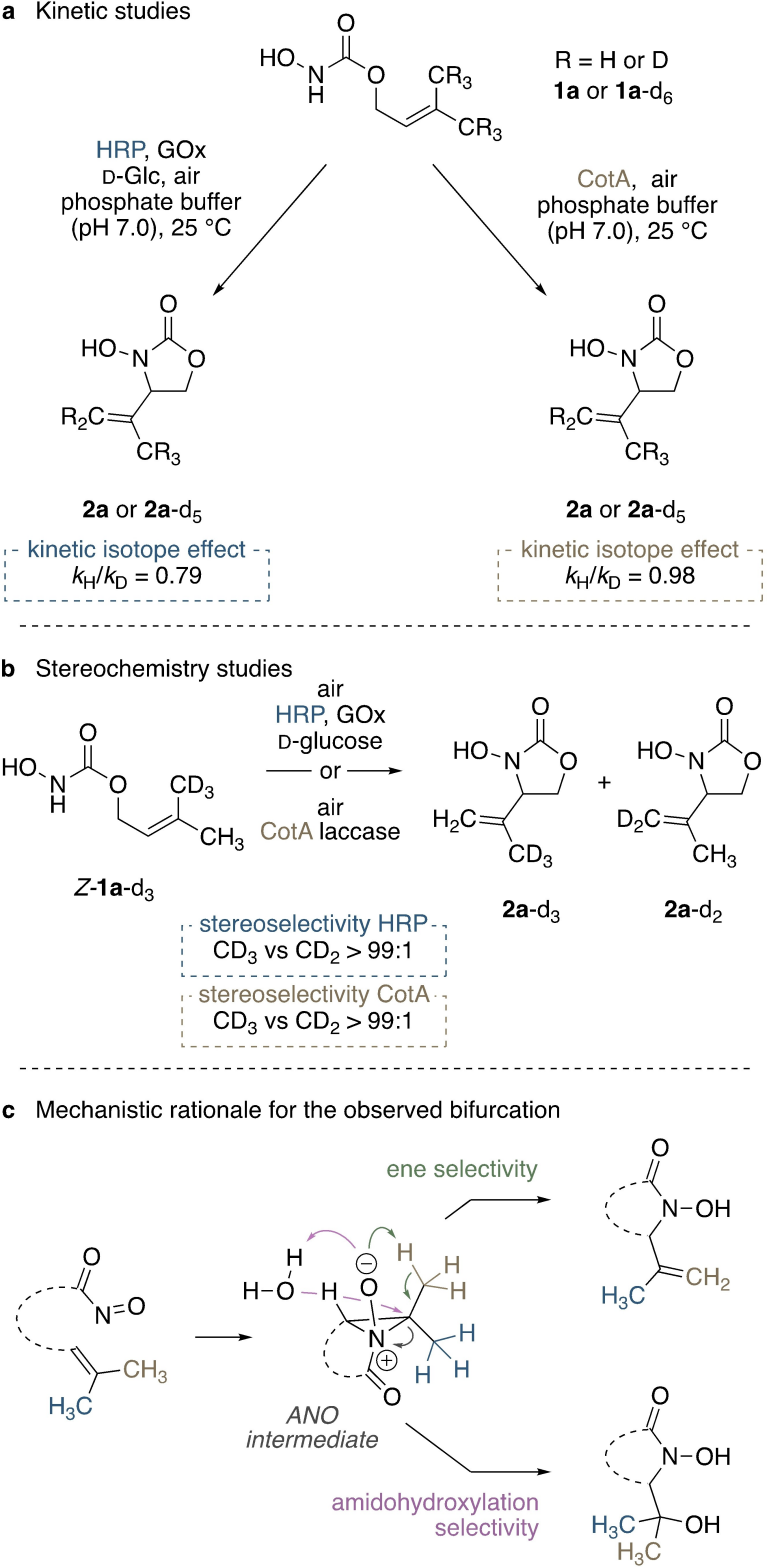
Enzyme‐mediated cyclizations of isotopomers of **1 a** provide hints on the underlying reaction pathways of aqueous nitroso ene reactions.

To shine light on the similarities and differences between the peroxidase‐ and the laccase‐mediated process, and to gain some insight into the mechanistic details of the uncommon aqueous nitroso ene chemistry, a series of isotopically labelled substrates were prepared to study the intramolecular conversion of **1 a** into the cyclic *N*‐hydroxycarbamate **2 a**. A symmetrically deuterated derivative **1 a**‐d_6_ was investigated, and comparison of initial rates of conversion of deuterated and non‐deuterated substrates revealed a strong inverse isotope effect (*k*_H_/*k*_D_=0.79; Scheme 7a). This isotope effect likely arises from the hybridization change of carbon C2 in the double bond (sp^2^ to sp^3^) during the rate‐limiting step. Similar inverse β secondary effects have not been previously observed experimentally in nitroso ene reactions, though they were also not ruled out in the first place. As competing diradical or dipolar pathways would most certainly lead to more significant normal isotope effects, it is believed that a β secondary isotope effect could not be recognized.[^26^, ^27^, ^28^, ^29^, ^30^] These expected effects of diradical or non‐ANO zwitterionic pathways are therefore not consistent with our observation, leading to the exclusion of these mechanistic options. Hence, the formation of an ANO as an intermediate instead of an inoperative bystander would be the logical conclusion. The absence of any primary KIE indicates a stepwise mechanism with irreversible formation of an intermediate in the rate‐limiting step followed by the hydrogen migration. Similar effects were observed by Orfanopoulos and co‐workers in the ene reaction of triazolinediones with phorone‐*d_6_* and styrene‐like substrates, who concluded an S_N_2‐type addition of the N=N enophile to the double bond occurred to form a three‐membered ANO‐like intermediate in the rate‐determining step.^[31]^ However, no distinct kinetic isotope effect was observed in the CotA‐catalyzed transformations, offering a strong hint that the two different enzymatic systems perform by different modi operandi, both proceeding via acylnitroso and ANO intermediates, yet most likely involving a copper‐bound activated nitroso species in the latter case.^[32]^ In‐depth investigations on differences and similarities to related established laccase/oxoammonium systems are currently underway.^[19]^

The clear on/off‐selectivity in the conversion of stereochemically defined isomers (*E* vs *Z*) was already illustrated in Scheme 4; a feature that is in line with a geometrically rigid intermediate. such as the aziridine *N*‐oxide. At the same time, this specificity stands in contrast to other protocols that do allow the transformation of *Z*‐olefins in intramolecular nitroso ene reactions,^[5c]^ supporting the possible deviation in pathways between polar‐protic and the more commonly used lipophilic media. To probe the expected selectivity for the cleavage of *E*‐oriented C−H bonds also in a sterically truly unbiased system, we furthermore synthesized unsymmetrically labelled *Z*‐**1 a**‐d_3_ and subjected it to the established enzyme‐mediated cyclizations. The thus obtained oxazolidinone **2 a**‐d_3_ carried the deuterium label specifically in the remaining methyl group (Scheme 7b), in both the peroxidase‐ and the laccase‐mediated reactions. Likewise, specific formation of the methylene‐labelled **2 a**‐d_2_ was observed when starting from the isomeric *E*‐**1 a**‐d_3_ (see the Supporting Information).

Combining the different findings—the inverse KIE, the stereospecificity and the unexpected formation of amidohydroxylation product **7**—the previously proposed aziridine *N*‐oxide as a relevant reaction intermediate seems plausible, providing a rationale for both the unusual limitation to *E*‐configured olefins and the bifurcation through water addition to the electrophilic aziridinium species (Scheme 7c). The apparent differences in observed kinetic isotope effects (Scheme 7a) and enantioselectivity (Scheme 3) further strengthen the picture that peroxidases and laccases operate in distinctly different ways.

All experimental evidence suggests that the HRP acts as a proteinogenic oxidizer with the ability to generate free acylnitroso intermediates that subsequently cyclize independently of the peroxidase, likely through formation of a rigid ANO intermediate. While the laccase‐catalyzed process shares the selectivity associated with the aziridine‐*N*‐oxide, ANO formation seems to be genuinely catalyzed by the blue‐copper enzyme, providing a stereochemically defined environment for the enantioselective C−N bond creation and eliminating the strong secondary isotope effect that would be in line with an uncatalyzed nitroso ene reaction.

## Conclusion

To conclude, two very mild C−N bond‐forming methods based on a nitroso ene pathway were developed, offering another important example of the synthetic potential of catalytically promiscuous enzymes. Utilizing a robust and reliable enzymatic couple consisting of horseradish peroxidase and glucose oxidase, reactive acylnitroso intermediates are generated in a highly selective manner, allowing both intra‐ and intermolecular aminations to take place in high yields in an aqueous medium. Our study further revealed that also certain laccases provide the necessary redox profile and protective environment to conduct nitroso‐ene‐type transformations. Classifying as genuinely biocatalytic, this process offers a first clear blueprint for the engineering of enantioselective versions of this attractive reaction. In addition to the promising starting point for stereochemical optimization, the observation of side reactivities, such as [4+2] cycloaddition and oxidative 1,2‐amidohydroxylation reactions underlines the yet untapped potential of these unprecedented enzymatic activation pathways, which will be addressed in much more detail in our future investigations.

## Conflict of interest

The authors declare no conflict of interest.

1

## Supporting information

As a service to our authors and readers, this journal provides supporting information supplied by the authors. Such materials are peer reviewed and may be re‐organized for online delivery, but are not copy‐edited or typeset. Technical support issues arising from supporting information (other than missing files) should be addressed to the authors.

Supporting Information

## Data Availability

The data that support the findings of this study are available in the supplementary material of this article.
