# Supplementary material for: Aerobic C−N Bond Formation through Enzymatic Nitroso‐Ene‐Type Reactions
Source: Angew Chem Int Ed Engl. 2023 Jan 9;62(7):e202213671. doi: 10.1002/anie.202213671 (PMC10107922; doi:10.1002/anie.202213671)
Supplement: Supplementary file 1 — Supporting Information [file ANIE-62-0-s001.pdf]

Angewandte Chemie
Eine Zeitschrift der Gesellschaft Deutscher Chemiker

Supporting Information

Aerobic C–N Bond Formation through Enzymatic Nitroso-Ene-Type Reactions

*C. Jäger, M. Haase, K. Koschorreck, V. B. Urlacher, J. Deska**

Table of Contents

General Remarks……………………………………………………………………….……..…….3

Procedures and analytical data…………………………………………………………...…………4

Synthesis of hydroxamic acids………………………………………….……………..…4

Enzymatic intramolecular nitroso-ene reactions…………………..………………14

Enzymatic intramolecular nitroso-Diels-Alder reactions…………………….….…...20

Enzymatic intermolecular nitroso-ene reactions…………………..………………21

Variability & turnover numbers..22

Laccase screening……………………………………………………………………………23

Peroxidase screening…………………………………………………...……………………...…27

Time course analysis……………………………………………………….……………………29

Michaelis-Menten kinetics………..………………………………………………..…..30

Kinetic isotope effect studies…………………………… ………………………………………..31

Decomposition analysis...……………………………………… ………………………………..33

HPLC traces…………………………………………………… ………………………………..34

NMR spectra……………………………………………………………………………………..…36

Supplementary References…………………………………………………….…………………..66

General Remarks

Commercially available reagents were used without further purification. Acetone-d_6 was purchased from Eurisotop. Glucose oxidase (Type X-S, lyophilized powder, 147019 U/g, *Aspergillus niger*), horseradish peroxidase (lyophilized powder, beige, 173 U/mg, RZ 3.39 (A_{403nm}/A_{280nm})), chloroperoxidase (aqueous suspension, 37332 U/mL *Caldariomyces fumago*), and bovine lactoperoxidase were purchased from Sigma Aldrich and used as obtained.

Recombinant production of *Bacillus licheniformis* CotA laccase mutant K316N/D500G (12.0 mg/mL),[1] *Trametes* versicolor Lccβ (0.57 mg/mL),[2] *Moniliophthora roreri* Mrl2 (5.85 mg/mL),[3] and *Streptomyces sviceus* Ssl1 laccase [1,4] was conducted as previously described. The enzyme activity U/mL was determined by measuring the change of absorption of ABTS (µmol/min) using UV-VIS. The enzyme was diluted with a factor of 100000 in phosphate buffer (pH 7.0, 100mM). In each well of a well-plate were added 180 µL of the enzyme solution and 20 µL of ABTS solution (5 mM in phosphate buffer). The absorption was measured at 420 nm over 5 min yielding following enzyme activities: CotA (*Bacillus licheniformis*) 261.4 U/mL, Lccβ (*Trametes versicolor*) 0.81 U/mL, Mrl2 (*Moniliophthora roreri)* 633.3 U/mL, Ssl1 (*Streptomyces sviceus*) 12.0 U/mL and Mtl (*Myceliophthora thermophila*) 996.7 U/mL. Protein concentrations and purity numbers (RZ) were determined on a Denovix DS-11+ microvolume spectrophotometer.

All chemical reactions were carried out under argon atmosphere and performed with dry solvents, if not stated differently. Solvents were dried with the help of a solvent drying system MB-SPS-800 from M. Braun. All enzymatic reactions were carried out under non-inert conditions. Yields are reported as the average of at least two reactions (except for *Z*-**1k** and **1m**), individual standard deviations are reported in Supplementary Table S2.

Silica gel from Merck (Millipore 60, 40-60 µm, 240-400 mesh) was used for column chromatography and silica pad filtrations. Reactions were monitored via thin layer chromatography (TLC) using precoated silica gel plates from Machery-Nagel (TLC Silica gel 60 F_{254}). The spots were identified using irradiation with UV-light and a staining solution (basic potassium permanganate solution).

^{1}H- and ^{13}C-NMR spectra were measured with a Bruker Avance NEO 400 at 20 °C. The chemical shifts are reported in ppm related to the signal of residual solvent of $CDCl_3$ (^{1}H: ($CDCl_3$) = 7.26 ppm, ^{13}C: ($CDCl_3$) = 77.2 ppm). Infrared spectra were recorded on a Bruker Alpha Eco ATR FTIR device. High resolution mass spectrometry was performed on an Agilent 6530 QTOF spectrometer. Melting points were measured on a Bibby Scientific Stuart melting point SMP30 with an error of ±0.5 °C. Melting points were determined by three individual measurements for one substance obtaining the temperature of the melting start and the fully melted state (the median of these been reported as the melting point).

HPLC measurements were performed at an Agilent 1100 system with a G1312A binary pump and a G 1312B diode array detector using a Chiralpak IA column from Daicel; method: 3% IPA in *n*-hexane, 0.8mL/min

Procedures and Analytical Data

Synthesis of hydroxamic acids

Representative general procedure: To a 0.2 M solution of alcohol (1.0 eq.) in dry acetonitrile was added CDI (1.5 eq.) under argon atmosphere. After full conversion of the alcohol (followed by TLC), imidazole (4.0 eq) and hydroxylamine hydrochloride (5.0 eq.) were added. After observing full conversion of the intermediate, the solvent was removed under reduced pressure. The resulted solid was dissolved in EtOAc and 1M HCl solution. The phases were separated, and the aqueous phase was washed 3x with EtOAc. The combined organic phases were dried over Na_2SO_4, filtered and the solvent was removed under reduced pressure. The crude was purified via flash column chromatography on silica gel.[5]

3-Methylbut-2-en-1-yl hydroxycarbamate (1a)

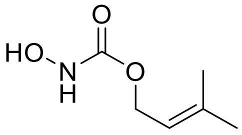

1a

According to the representative general procedure, 3-methylbut-2-en-1-ol (1.2 mL, 1.03 g, 11.98 mmol) was reacted. The crude was purified via column chromatography on silica gel (4:1 *n*-Hep/EtOAc; R_f = 0.32 (3:2 *n*-Hep/EtOAc)) to yield **1a** as a colorless oil (1.62 g, 11.18 mmol, 145.16 g/mol, 93 %). Spectroscopic data are in agreement with literature precedent.[5]

^{1}H-NMR (400 MHz, $CDCl_3$) δ [ppm] = 7.23 (s, 1H, -NH-), 6.85 (bs, 1H, -OH), 5.41-5.27 (m, 1H), 4.65 (d, *J* = 7.3 Hz, 2H), 1.76 (d, *J* = 0.8 Hz, 3H), 1.71 (d, *J* = 0.9 Hz, 3H). **^{13}C-NMR** (100 MHz, $CDCl_3$) δ [ppm] = 159.5, 140.2, 118.1, 63.1, 25.8, 18.1. **FT-IR** (ATR) ν [cm^{-1}] = 3290 (w), 2933 (vw), 1706 (s), 1445 (m), 1259 (m), 1101 (s).

3-(Methyl-d$_3$)but-2-en-1-yl-4,4,4-d$_3$ hydroxycarbamate (1a-d$_6$)

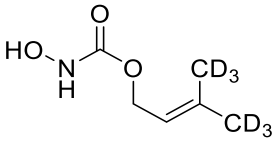

1a-d$_6$

To a solution of triethyl phosphonoacetate (4.1 mL, 20.4 mmol, 1.5 eq.) in 35 mL THF was added *n*-BuLi (7.6 mL, 1.4 eq, 2.5 M in hexanes) at 0 °C. After full addition, the reaction mixture was allowed to warm to room temperature. After 30 min, acetone-*d6* (1 mL, 13.6 mmol, 1.0 eq.) was added. After 5 h, the reaction was quenched with saturated aqueous NH_4Cl solution. The aqueous phase was extracted 3x with Et_2O. The combined organic phases were washed with brine, dried over Na_2SO_4, filtered and the solvent was removed under reduced pressure. The crude was purified via column chromatography on silica gel (2:1 *n*-Pen/Et_2O) to yield ethyl 3-(methyl-d$_3$)but-2-enoate-4,4,4-d$_3$ (1.54 g, 11.5 mmol, 134.21 g/mol, 85 %) Spectroscopic data are in agreement with literature precedent.[12]

^{1}H-NMR (400 MHz, $CDCl_3$): δ [ppm] = 5.67 (s, 1H), 4.14 (q, *J* = 7.1 Hz, 2H), 1.27 (t, J = 7.1 Hz, 3H).

To a solution of 3-(methyl-d$_3$)but-2-enoate-4,4,4-d$_3$ (1.12 g, 8.9 mmol, 1.0 eq.) in 10 mL DCM was added DIBAL-H (27 mL, 3.0 eq., 1 M in *c*-Hex) at 0 °C. After 2 h the reaction was quenched with MeOH. The mixture was poured to 30 mL Et_2O and 30 mL of 2 M HCl solution was added. The aqueous phase was extracted 3x with Et_2O. The combined organic layers were washed with 1 M HCl solution and 2x with brine, dried over Na_2SO_4, filtered and the solvent was removed under reduced pressure. The crude was purified via bulb-to-

bulb distillation to afford 3-(methyl-d$_3$)but-2-en-4,4,4-d$_3$-1-ol (450 mg, 4.9 mmol, 92.17 g/mol, 55 %). Spectroscopic data are in agreement with literature precedent.[13]

^{1}H-NMR (400 MHz, $CDCl_3$): δ [ppm] = 5.41 (t, *J* = 7.1 Hz, 1H), 4.12 (d, *J* = 7.0 Hz, 2H), 1.21 (s, 1H, -OH).

According to the representative general procedure, 3-(methyl-d$_3$)but-2-en-4,4,4-d$_3$-1-ol (200 mg, 2.2 mmol) was reacted. The crude was purified via column chromatography on silica gel (2:1 *n*-Pen/Et_2O; R_f = 0.15 (2:1 *n*-Hep/EtOAc)) to yield **1a-d$_6$** as a colorless oil (151.2 mg, 1.5 mmol, 229.4 g/mol, 69 %, D_6 ≥ 99 %).

^{1}H-NMR (400 MHz, $CDCl_3$): δ [ppm] = 7.26 (s, 1H, -NH-), 7.06 (s, 1H, -OH), 5.34 (t, *J* = 7.3 Hz, 1H), 4.65 (d, *J* = 7.3 Hz, 2H). **FT-IR** (ATR) ν [cm^{-1}] = 3294 (w), 2958 (vw), 1705 (m), 1463 (w), 1257 (s), 1104 (s). **HRMS** (ESI): *m/z* [M+Na$^+$] calcd for $C_6H_5D_6NO_3$: 174.1008; found: 174.1014.

E-3-Methylbut-2-en-1-yl-4,4,4-d3 hydroxycarbamate (1a-*E*-d$_3$)

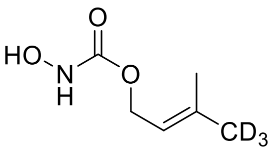

1a-*E*-**d$_3$**

To a solution of thiophenol (1.7 g, 15.44 mmol, 1.2 eq.) in 30 mL EtOH was added NaOH (618 mg, 15.44 mmol, 1.2 eq.). After stirring for 30 min at room temperature, a solution of ethyl but-2-ynoate (1.5 mL, 12.87 mmol, 1.0 eq.) in 5 mL EtOH was added. After 4 h the reaction was slightly acidified with diluted acetic acid. The aqueous phase was extracted 3x with Et_2O and the combined organic phases were washed with 4 % NaOH solution, H_2O, brine and dried over Na_2SO_4, filtered and the solvent was removed under reduced pressure. The crude was purified via column chromatography (*n*-Hep/EtOAc 30:1; R_f = 0.54 & 0.44 (6:1 *n*-Hep/EtOAc)) to yield and separate ethyl *Z*-3-(phenylthio)but-2-enoate (460 mg, 2.07 mmol, 222.3 g/mol 16 %) and ethyl *E*-3-(phenylthio)but-2-enoate (1.88 g, 8.47 mmol, 222.3 g/mol 66 %). Spectroscopic data are in agreement with literature precedent.[22]

E-Isomer

^{1}H-NMR (400 MHz, $CDCl_3$): δ [ppm] = 7.45-7.41 (m, 2H), 7.39-7.34 (m, 3H), 5.19 (d, *J* = 1.1 Hz, 1H), 4.02 (q, *J* = 7.1 Hz, 2H), 2.37 (d, *J* = 1.1 Hz, 3H), 1.15 (t, *J* = 7.1 Hz, 3H). **^{13}C-NMR** (100 MHz, $CDCl_3$): δ [ppm] = 165.6, 159.8, 135.6, 130.0, 129.9, 111.1, 59.7, 20.2, 14.4.

Z-Isomer

^{1}H-NMR (400 MHz, $CDCl_3$): δ [ppm] = 7.57-7.48 (m, 2H, -Ph), 7.45-7.32 (m, 3H, -Ph), 5.85 (d, *J* = 1.1 Hz, 1H, H-2), 4.22 (q, *J* = 7.1 Hz, 2H, H-5), 1.81 (d, *J* = 1.1 Hz, 3H, H-4), 1.30 (t, *J* = 7.1 Hz, 3H, H-6). **^{13}C-NMR** (100 MHz, $CDCl_3$): δ [ppm] = 166.3, 158.3, 136.1, 130.9, 129.5, 129.1, 111.9, 59.9, 25.1, 14.4.

CuI (648 mg, 3.4 mmol, 2.0 eq.) was dispensed in 18 mL THF. CD_3MgI (9 mL, 5.0 eq., 1M in Et_2O) was slowly added at -78 °C and the reaction mixture was stirred for 15 min at that temperature. Ethyl *E*-3-(phenylthio)but-2-enoate (400 mg, 1.8 mmol, 1.0 eq.) in 9 mL THF was slowly added at -78 °C. The reaction mixture was stirred for 8 h at the same temperature and was then quenched with saturated NH_4Cl solution at 0 °C. The aqueous phase was extracted 2x with Et_2O. The combined organic phases were washed with 5 % NaOH solution, H_2O, brine and dried over Na_2SO_4, filtered and the solvent was removed under reduced pressure (max. 350 mbar at 30 °C). The crude was purified via column chromatography (*n*-Pen/Et_2O 30:1; R_f = 0.76 (2:1 *n*-Hep/EtOAc)) to yield ethyl *E*-3-methylbut-2-enoate-4,4,4-d$_3$ in residual Et_2O. Spectroscopic data are in agreement with literature precedent.[22]

^{1}H-NMR (400 MHz, $CDCl_3$): δ [ppm] = 5.66 (d, *J* = 1.0 Hz, 1H), 4.13 (q, *J* = 7.1 Hz, 2H), 2.15 (d, *J* = 1.2 Hz, 3H), 1.26 (t, *J* = 7.1 Hz, 3H).

To a solution of the obtained ethyl *E*-3-methylbut-2-enoate-4,4,4-d_3 in residual Et_2O and 1 mL DCM was added DIBAL-H (3.2 mL, 3.0 eq., 1M in *c*-Hex) at 0 °C. After full conversion (followed by TLC), the reaction was quenched with MeOH followed by 1M HCl. The aqueous phase was extracted 2x with Et_2O. The combined organic phases were washed with brine, dried over Na_2SO_4, filtered and the solvent was removed under reduced pressure (volatile!). The crude was purified via bulb-to-bulb distillation to yield *E*-3-methylbut-2-en-4,4,4-d_3-1-ol in residual Et_2O. Spectroscopic data are in agreement with literature precedent.[22]

^{1}H-NMR (400 MHz, $CDCl_3$): δ [ppm] = 5.41 (td, *J* = 7.1, 1.3 Hz, 1H), 4.13 (d, *J* = 7.1 Hz, 2H), 1.68 (s, 3H).

According to the representative general procedure, *E*-3-methylbut-2-en-4,4,4-d_3-1-ol in residual Et_2O was reacted. The crude was purified via column chromatography on silica gel (3:1 *n*-Hep/EtOAc; R_f = 0.21 (2:1 *n*-Hep/EtOAc)) to yield **1a**-*E*-**d₃** as a colorless oil (49 mg, 10.33 mmol, 148.18 g/mol, 18 % over 3 steps, $D_3 \geq$ 95 %).

^{1}H-NMR (400 MHz, $CDCl_3$): δ [ppm] = 7.18 (s, 1H), 6.58 (s, 1H), 5.35 (td, *J* = 7.3, 1.2 Hz, 1H), 4.66 (d, *J* = 7.3 Hz, 2H), 1.72 (d, *J* = 0.7 Hz, 3H). **FT-IR** (ATR) ν [cm^{-1}] = 3295 (w), 2917 (vw), 1705 (m), 1456 (w), 1257 (m), 1102 (s). **HRMS** (ESI): *m/z* [$M+H^+$] calcd for $C_6H_8D_3NO_3$: 149.1000; found: 149.1007.

Z-3-Methylbut-2-en-1-yl-4,4,4-d₃ hydroxycarbamate (1a-*Z*-d₃)

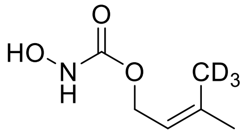

1a-*Z*-**d₃**

Z-3-methylbut-2-en-4,4,4-d_3-1-ol was synthesized from ethyl *Z*-3-(phenylthio)but-2-enoate like described before.[22] According to the representative general procedure, *Z*-3-methylbut-2-en-4,4,4-d_3-1-ol in residual Et_2O was reacted. The crude was purified via column chromatography on silica gel (3:1 *n*-Hep/EtOAc; R_f = 0.37 (1:1 *n*-Hep/EtOAc)) to yield **1a**-*Z*-**d₃** as a colorless oil (75 mg, 0.5 mmol, 148.18 g/mol, 28 % over 3 steps, $D_3 \geq$ 95 %).

^{1}H-NMR (400 MHz, $CDCl_3$): δ [ppm] = 7.21 (s, 1H), 6.73 (s, 1H), 5.35 (t, *J* = 7.3 Hz, 1H), 4.65 (dd, *J* = 7.3, 0.7 Hz, 2H), 1.76 (s, 3H). **FT-IR** (ATR) ν [cm^{-1}] = 3298 (w), 2915 (vw), 1705 (m), 1445 (w), 1289 (m), 1112 (s). **HRMS** (ESI): *m/z* [$M+H^+$] calcd for $C_6H_8D_3NO_3$: 149.1000; found: 149.0986.

N-Hydroxy-5-methylhex-4-enamide (1b)

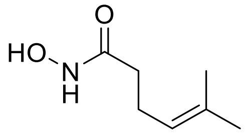

1b

Methyl 5-methylhex-4-enoate was prepared from 2-methylbut-3-en-2-ol according to literature precedent.[21] To 30 mL of a 2 M solution of hydroxylamine hydrochloride in MeOH was added 30 mL of a 4 M solution of KOH in MeOH at 0 °C. The formed KCl was filtered off and the mixture was added to methyl 5-methylhex-4-enoate (1 g, 7.03 mmol) and adjusted to pH 10.0 using a solution of KOH in MeOH. The reaction mixture was stirred for 4 h and then adjusted to pH 5.0 with a 5 M HCl solution. The solvent was removed under reduced pressure and the resulting solid was dissolved in MeOH. Remaining insoluble solid was removed via filtration. The solvent was again removed under reduced pressure. This step was repeated with DCM. The crude was

purified via column chromatography (DCM + 4 % MeOH; R_f = 0.28 (DCM + 3 % MeOH)) to yield **1b** as a beige solid (338.3 mg, 2.36 mmol, 143.19 g/mol, 34 %). Spectroscopic data are in agreement with literature precedent.[8]

^{1}H-NMR (400 MHz, $CDCl_3$): δ [ppm] = 8.54 (bs, 2H, -NH-, -OH), 5.06 (t, *J* = 6.7 Hz, 1H), 2.32 (q, *J* = 7.0 Hz, 2H), 2.18 (t, *J* = 7.2 Hz, 2H), 1.69 (s, 3H), 1.61 (s, 3H). **^{13}C-NMR** (100 MHz, $CDCl_3$): δ [ppm] = 171.3, 134.1, 121.9, 33.2, 25.7, 23.8, 17.7. **FT-IR** (ATR) ν [cm^{-1}] = 3200 (m), 2910 (m), 1624 (vs), 1539 (s), 1067 (s). **Melting point**: 59.8 °C.

_E_-But-2-en-1-yl hydroxycarbamate (1c)

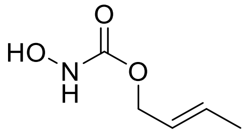

1c

According to the representative general procedure, crotyl alcohol (0.5 ml, 5.9 mmol) was reacted. The crude was purified via column chromatography on silica gel (*n*-Hep/EtOAc 4:1; R_f = 0.26 (2:1 *n*-Hep/EtOAc)) to yield **1c** as a colorless oil (49.8 mg, 0.37 mmol, 131.13 g/mol, 6 %). Spectroscopic data are in agreement with literature precedent.[6]

^{1}H-NMR (400 MHz, $CDCl_3$): δ [ppm] = 7.23 (s, 1H, -NH-), 6.72 (bs, 1H, -OH), 5.90-5.76 (m, 1H), 5.65-5.51 (m, 1H), 4.58 (d, *J* = 6.6 Hz, 2H), 1.72 (dd, *J* = 6.6, 1.3 Hz, 3H). **^{13}C-NMR** (100 MHz, $CDCl_3$): δ [ppm] = 132.4, 124.6, 66.9, 17.8. **FT-IR** (ATR) ν [cm^{-1}] = 3290 (w), 2945 (vw), 1705 (s), 1449 (m), 1260 (m), 1105 (s).

_E_-2-Methylbut-2-en-1-yl hydroxycarbamate (1d)

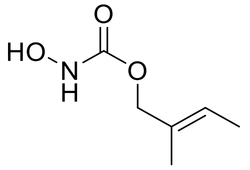

1d

To a solution of *E*-2-methylbut-2-enal (0.5 mL, 5.17 mmol) in 8 mL MeOH was added $NaBH_4$ (391 mg, 10.34 mmol) at 0 °C. After 1 h, the reaction mixture was quenched with water and extracted 2x with DCM. The combined organic phases were washed with brine, dried over $MgSO_4$, filtered and the solvent was removed under reduced pressure. The crude was purified via bulb-to-bulb distillation to afford *E*-2-methylbut-2-en-1-ol (358 mg, 4.16 mmol, 80 %).

^{1}H-NMR (400 MHz, $CDCl_3$): δ [ppm] = 5.57- 5.40 (m, 1H), 3.99 (d, *J* = 3.7 Hz, 2H), 1.67 (s, 3H), 1.62 (ddd, *J* = 6.7, 2.1, 1.0 Hz, 3H), 1.35 (bs, 1H, -OH). **^{13}C-NMR** (100 MHz, $CDCl_3$): δ [ppm] = 135.6, 120.8, 69.2, 13.5, 13.2.

According to the representative general procedure, *E*-2-methylbut-2-en-1-ol (200 mg, 2.3 mmol) was reacted. The crude was purified via column chromatography on silica gel (*n*-Hep/EtOAc 4:1; R_f = 0.21 (2:1 *n*-Hep/EtOAc)) to yield **1d** as a colorless oil (70.3mg g, 0.48 mmol, 145.16 g/mol, 21 %).

^{1}H-NMR (400 MHz, $CDCl_3$): δ [ppm] = 7.31 (s, 1H, -NH-), 7.09 (bs, 1H, -OH), 5.67-5.47 (m, 1H), 4.53 (s, 2H), 1.64-1.62 (m, 6H). **^{13}C-NMR** (100 MHz, $CDCl_3$): δ [ppm] = 159.5, 130.4, 125.0, 72.1, 13.5, 13.3. **FT-IR** (ATR) ν [cm^{-1}] = 3289 (w), 2920 (vw), 1704 (s), 1447 (m), 1263 (s), 1100 (s). **HRMS** (ESI): *m/z* [M+Na$^+$] calcd for $C_6H_{11}NO_3$: 168.0631; found: 168.0634.

2-Cyclohexylideneethyl hydroxycarbamate (1e)

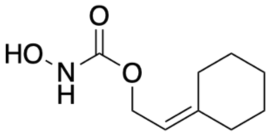

1e

To a solution of cyclohexanone (3.24 g, 33.0 mmol, 1.0 eq.) in 45 mL toluene was added Methyl (triphenylphosphoranylidene)acetate (13.24 g, 39.6 mmol, 1.2 eq.). The reaction mixture was stirred for 23 h at 110 °C. The solid was filtered off and washed with EtOAc. The solvent was removed under reduced pressure and the resulted solid was again filtered off and washed with EtOAc. The solvent was again removed under reduced pressure and the crude was purified via column chromatography (8:1 n-Hep/EtOAc + 3 % TEA) to afford methyl 2-cyclohexylideneacetate as a yellow oil (2.15 g, 14.0 mmol, 42 %). Spectroscopic data are in agreement with literature precedent.[10]

^{1}H-NMR (400 MHz, $CDCl_3$): δ [ppm] = 5.67-5.53 (m, 1H), 3.67 (s, 2H), 2.82 (m, 2H), 2.23-2.14 (m, 2H), 1.70-1.54 (m, 6H). **^{13}C-NMR** (100 MHz, $CDCl_3$): δ [ppm] = 167.4, 164.0, 112.7, 50.9, 38.1, 30.0, 28.8, 28.0, 26.4.

To a solution of 2-cyclohexylideneacetate (2.11 g, 13.7 mmol, 1.0 eq.) in 56 mL DCM was added DIBAL-H (38.4 mL, 38.4 mmol, 1 M in *c*-Hex) at -78 °C. The reaction mixture was allowed to warm to 0 °C after 3 h. The reaction was quenched with water after 25 h. The mixture was washed with 1 M HCl solution. The aqueous phase was extracted 3x with DCM. The combined organic phases were dried over Na_2SO_4, filtered and the solvent was removed under reduced pressure. The crude was purified via silica pad filtration (2:1 *n*-Hep/EtOAc) to afford 2-cyclohexylideneethan-1-ol as a colorless oil (1.57 g, 12.4 mmol, 91 %). Spectroscopic data are in agreement with literature precedent.[11]

^{1}H-NMR (400 MHz, $CDCl_3$): δ [ppm] = 5.34 (t, *J* = 7.1, 1H), 4.12 (d, *J* = 7.1 Hz, 2H), 2.17 (t, *J* = 5.6 Hz, 2H), 2.10 (t, *J* = 5.7 Hz, 2H), 1.47-1.57 (m, 6H), 1.39 (t, *J* = 6.2 Hz, 1H). **^{13}C-NMR** (100 MHz, $CDCl_3$): δ [ppm] = 144.50, 120.4, 58.6, 37.2, 29.0, 28.5, 28.0, 26.81.

According to the representative general procedure, 2-cyclohexylideneethan-1-ol (500 g, 3.96 mmol) was reacted. The crude was purified via column chromatography on silica gel (*n*-Hep/EtOAc 3:1; R_f = 0.44 (1:1 *n*-Hep/EtOAc)) to yield **1e** as a white solid (530 mg g, 2.87 mmol, 185.22 g/mol, 73 %). Spectroscopic data are in agreement with literature precedent.[7]

^{1}H-NMR (400 MHz, $CDCl_3$): δ [ppm] = 7.28 (s, 1H, -NH-), 7.13 (s, 1H, -OH), 5.28 (t, *J* = 7.4 Hz, 1H), 4.66 (d, *J* = 7.4 Hz, 2H), 2.19 (s, 2H), 2.11 (s, 2H), 1.55 (s, 6H). **^{13}C-NMR** (100 MHz, $CDCl_3$): δ [ppm] = 159.6, 148.0, 114.6, 62.4, 37.0, 29.0, 28.3, 27.7, 26.6. **FT-IR** (ATR) ν [cm^{-1}] = 3267 (w), 2928 (w), 1714 (m), 1442 (m), 1258 (m), 1104 (s). **Melting point**: 67.0 °C.

***E*-Pent-3-en-2-yl hydroxycarbamate (1f)**

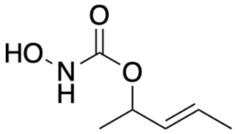

1f

According to the representative general procedure, *E*-pent-3-en-2-ol (357 mg, 4.2 mmol) was reacted. The crude was purified via column chromatography on silica gel (*n*-Hep/EtOAc 4:1; R_f = 0.16 (2:1 *n*-Hep/EtOAc)) to yield **1f** as a white solid (382 mg g, 2.6 mmol, 145.16 g/mol, 63 %). Spectroscopic data are in agreement with literature precedent.[9]

^{1}H-NMR (400 MHz, $CDCl_3$): δ [ppm] = 7.11 (s, 1H, -NH-), 6.43 (s, 1H, -OH), 5.83-5.68 (m, 1H), 5.47 (dddd, *J* = 15.3, 7.0, 3.2, 1.5 Hz, 1H), 5.29 (p, *J* = 6.6 Hz, 1H), 1.69 (d, *J* = 6.5 Hz, 3H), 1.32 (d, *J* = 6.4 Hz, 3H). **^{13}C-**

NMR (100 MHz, $CDCl_3$): δ [ppm] = 159.1, 130.2, 129.1, 73.7, 20.4, 17.7. **FT-IR** (ATR) ν [cm^{-1}] = 3343 (w), 3242 (w), 2982 (vw), 1697 (s), 1505 (m), 1276 (m), 1114 (s). **Melting point**: 41.2 °C.

S-(4-(Prop-1-en-2-yl)cyclohex-1-en-1-yl)methyl hydroxycarbamate (1g)

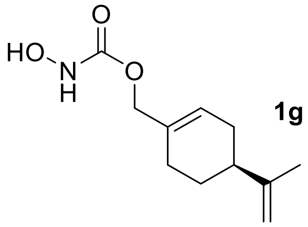

1g

According to the representative general procedure, *S*-(-)-perillyl alcohol (0.5 mL, 3.2 mmol) was reacted. The crude was purified via column chromatography on silica gel (*n*-Hep/EtOAc 4:1 → 2.1; R_f = 0.56 (1:1 *n*-Hep/EtOAc)) to yield **1g** as a white solid (632 mg g, 3.0 mmol, 211.26 g/mol, 95 %).

^{1}H-NMR (400 MHz, $CDCl_3$): δ [ppm] = 7.27 (s, 1H, -NH-), 6.90 (s, 1H, -OH), 5.78 (s, 1H), 4.72 (d, *J* = 10.2 Hz, 2H), 4.54 (s, 2H), 2.22-1.80 (m, 6H), 1.73 (s, 3H), 1.54-1.41 (m, 1H). **^{13}C-NMR** (100 MHz, $CDCl_3$): δ [ppm] = 159.5, 149.5, 132.3, 126.7, 108.9, 70.2, 40.7, 30.4, 27.2, 26.3, 20.8. **FT-IR** (ATR) ν [cm^{-1}] = 3321 (vw), 3231 (vw), 2916 (vw), 1694 (s), 1518 (m), 1280 (m), 1118 (s). **Melting point**: 103.0 °C. **HRMS** (ESI): *m/z* [M+Na$^+$] calcd for $C_{11}H_{17}NO_3$: 234.1101; found: 234.1105.

4-Methylpent-3-en-1-yl hydroxycarbamate (1h)

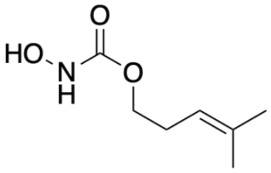

1h

A solution of trimethyl aluminum (2 M in hexanes, 13 mL, 26.4 mmol, 2.2 eq.) in 50 mL dry DCM was cooled to 0 °C and pent-3-yn-1-ol (1.0 g, 12 mmol, 1.0 eq.). The forming methane was removed using argon. The reaction mixture was cooled to -78 °C and an already cooled solution of $TiCl_4$ (2.2 g, 12 mmol, 1.0 eq.) in 33 mL dry DCM was slowly added. Stirring was continued for 3 h at -78 °C and subsequently quenched with 7 mL MeOH at 0 °C. The organic phase was washed with brine and 3 M HCl. The aqueous phase was extracted 5x with DCM, dried over $MgSO_4$, filtered and the solvent was removed under reduced pressure. The crude was purified via column chromatography on silica gel (5:1 *n*-Hep/EtOAc; R_f = 0.48 (1:1 *n*-Hep/EtOAc)) to yield 4-methylpent-3-en-1-ol as a colorless oil (0.49 g, 4.9 mmol, 100.16 g/mol, 41 %). Spectroscopic data are in agreement with literature precedent.[24]

^{1}H-NMR (400 MHz, $CDCl_3$): δ [ppm] = 5.10-5.19 (m, 1H), 3.64 (t, *J* = 6.5 Hz, 2H), 2.26-2.34 (m, 2H), 1.75 (s, 3H), 1.67 (s, 3H).

^{13}C-NMR (100 MHz, $CDCl_3$): δ [ppm] = 135.0, 120.0, 62.5, 31.6, 25.9, 17.9.

According to the representative general procedure, 4-methylpent-3-en-1-ol (260 mg, 2.6 mmol) was reacted. The crude was purified via column chromatography on silica gel (3:1 *n*-Hep/EtOAc; R_f = 0.42 (1:1 *n*-Hep/EtOAc)) to yield **1h** as a colorless oil (400 mg, 2.75 mmol, 159.19 g/mol, 88 %). Spectroscopic data are in agreement with literature precedent.[6]

^{1}H-NMR (400 MHz, $CDCl_3$): δ [ppm] = 7.50 (s, 1H), 6.89 (s, 1H), 5.06-5.13 (m, 1H), 4.13 (t, *J* = 7.2 Hz, 2H), 2.31-2.38 (m, 2H), 1.71 (s, 3H), 1.63 (s, 3H). **^{13}C-NMR** (100 MHz, $CDCl_3$): δ [ppm] = 159.7, 135.0, 118.8, 65.9, 27.8, 25.7, 17.8. **FT-IR** (ATR) v [cm^{-1}] = 3285 (vw), 2966 (vw), 1708 (s), 1449 (m), 1261 (m).

4,6,6-Trimethylbicyclo[3.1.1]hept-3-en-2-yl hydroxycarbamate (1i)

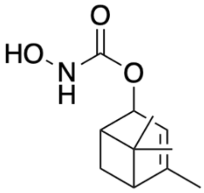

1i

To a solution of verbenon (300 mg, 2 mmol, 1.0 eq) in 2 mL Et_2O was added lithium aluminium hydride (2 mmol, 1.0 eq.) at -78 °C. After stirring for 30 min, the reaction mixture was allowed to warm to 0 °C. After 3 h the mixture was quenched with H_2O. The phases were separated, and the aqueous phase was extracted 2x with Et_2O. The combined organic phases were dried over Na_2SO_4, filtered and the solvent was removed under reduced pressure. The crude was used for the next step after a silica-pad filtration (EtOAc). According to the representative general procedure, crude Verbenol was reacted. The crude was purified via column chromatography on silica gel (n-Hep/EtOAc 4:1 → 2:1; R_f = 0.41 (1:1 *n*-Hep/EtOAc)) to yield **1i** as a colorless oil (221 mg, 1.15 mmol, 220.8 g/mol, 52 %).

^{1}H-NMR (400 MHz, $CDCl_3$): δ [ppm] = 7.15 (s, 1H), 6.74 (bs, 1H), 5.55-5.49 (m, 1H), 5.41-5.34 (m, 1H), 2.55-2.48 (m, 1H), 2.45-2.38 (m, 1H), 2.03 (t, *J* = 5.4 Hz, 1H), 1.77 (t, *J* = 1.6 Hz, 3H), 1.44-1.40 (m, 1H), 1.36 (s, 3H), 1.00 (s, 3H). **^{13}C-NMR** (100 MHz, $CDCl_3$): δ [ppm] = 159.4, 150.3, 115.2, 77.7, 47.5, 45.6, 39.7, 35.7, 26.6, 22.7, 22.6. **FT-IR** (ATR) v [cm^{-1}] = 3291 (vw), 2926 (w), 1712 (m), 1442 (w), 1249 (m), 1103 (s). **HRMS** (ESI): *m/z* [$M+H^+$] calcd for $C_{11}H_{17}NO_3$: 212.1282; found: 212.1283.

***Z*-3-Phenylbut-2-en-1-yl hydroxycarbamate (1j)**

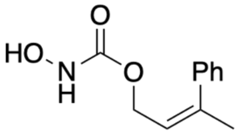

1j

To a suspension of CuI (0.43 g, 0.22 mmol, 0.05 eq.) and LiCl (0.19 g, 0.45 mmol, 0.1 eq.) in 34 mL THF was stirred for 30 min at –78 °C. Ethylbut-2-ynoate (0.5 g, 4.46 mmol, 1.0 eq.) and TMSOTf (1.04 g, 4.69 mmol, 1.05 eq.) were added and stirred for additional 5 min followed by a dropwise addition of PhMgBr (0.91 g, 5.45 mmol, 1.2 eq.). The reaction was followed by TLC and TFA (0.61 g, 5.35 mmol, 1.2 eq.) was added after full conversion of the substrate. The organic phase was washed with saturated $NaHCO_3$ solution. The combined aqueous phases were extracted 3x with EtOAc. The combined organic phases were washed with water and brine, dried over Na_2SO_4, filtered and the solvent was removed under reduced pressure. The crude was purified via column chromatography (19:1 *n*-Pen/Et_2O) to yield ethyl *E*-3-phenylbut-2-enoate (0.11 g, 0.58 mmol, 13 %) and ethyl *Z*-3-phenylbut-2-enoate (0.28 g, 1.47 mmol, 33 %) as colorless oils. Spectroscopic data are in agreement with literature precedent.[18]

Z-Isomer

^{1}H NMR (400 MHz, $CDCl_3$): δ [ppm] = 7.38-7.30 (m, 3H), 7.23-7.18 (m, 2H), 5.92 (q, *J* = 1.4 Hz, 1H), 4.00 (q, *J* = 7.1 Hz, 2H), 2.18 (d, *J* = 1.5 Hz, 3H), 1.08 (t, *J* = 7.1 Hz, 3H). **^{13}C NMR** (100 MHz, $CDCl_3$): δ [ppm] = 166.0, 155.4, 140.9, 127.9, 127.7, 126.8, 117.8, 59.7, 27.2, 14.0.

E-Isomer

^{1}H-NMR (400 MHz, $CDCl_3$): δ [ppm] = 7.50-7.46 (m, 2H), 7.41-7.34 (m, 3H), 6.14 (q, *J* =1.3 Hz, 1H), 4.22 (q, *J* = 7.1 Hz, 1H), 2.58 (d, *J* = 1.4 Hz, 3H), 1.32 (t, *J* = 7.0 Hz, 3H). **^{13}C-NMR** (100 MHz, $CDCl_3$): δ [ppm] = 167, 155.6, 142.4, 129.1, 128.6, 126.4, 117.3, 60.0, 18.1, 14.5.

To a solution of ethyl *Z*-3-phenylbut-2-enoate (0.52 g, 2.72 mmol, 1.0 eq.) in 11 mL DCM was added DIBAL-H (7.7 mL,7.7 mmol,2.8 eq.,1 M in *c*-Hex) at -78 °C. After 1.5 h the reaction mixture was warmed to 0 °C and quenched with EtOAc and water. The mixture was was washed with 1 M HCl solution. The phases were separated, and the aqueous phase was extracted 3x with DCM. The combined organic phases were dried over Na_2SO_4, filtered and the solvent was removed under reduced pressure. The crude was purified via column chromatography (2:1 *n*-Hep/EtOAc) to yield *Z*-3-phenylbut-2-en-1-ol (0.37 g, 2.59 mmol, 96 %) as a colorless oil. Spectroscopic data are in agreement with literature precedent.[19]

^{1}H-NMR (400 MHz, $CDCl_3$): δ [ppm] = 7.39-7.24 (m, 3H), 7.20-7.14 (m, 2H), 5.71 (tq, *J* = 7.0, 1.3 Hz, 1H), 4.07 (d, *J* = 7.0 Hz, 2H), 2.09 (dd, *J* = 2.4, 1.0 Hz, 3H). **^{13}C-NMR** (100 MHz, $CDCl_3$): δ [ppm] = 140.8, 140.2, 128.2, 127.8, 127.2, 126.1, 60.3, 25.4.

According to the representative general procedure, *Z*-3-phenylbut-2-en-1-ol (0.32 g, 2.16 mmol) was reacted. The crude was purified via column chromatography on silica gel (*n*-Hep/EtOAc 4:1 → 2.1; R_f = 0.18 (2:1 *n*-Hep/EtOAc)) to yield **1j** as a white solid (0.38 g, 1.86 mmol, 207.23 g/mol, 86 %).[23]

^{1}H-NMR (400 MHz, $CDCl_3$): δ [ppm] = 7.40-7.27 (m, 3H, -Ph), 7.21-7.14 (m, 2H, -Ph), 7.12 (s, 1H, -NH-), 6.20 (s, 1H, -OH), 5.67 (td, *J* = 7.2, 1.5 Hz, 1H), 4.59 (dd, *J* = 7.2, 0.9 Hz, 2H), 2.10 (d, *J* = 1.3 Hz, 3H). **^{13}C-NMR** (100 MHz, $CDCl_3$): δ [ppm] = 159.2, 143.7, 140.2, 128.3, 127.7, 127.5, 120.5, 64.1, 25.5. **FT-IR** (ATR) ν [cm^{-1}] = 3298 (w), 1703 (m), 1482 (m), 1266 (m), 1126 (s), 761 (s). **Melting point**: 61.6 °C.

_E_-Hex-2-en-1-yl hydroxycarbamate (_E_-1k)

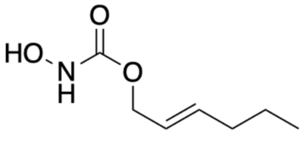

E-**1k**

According to the representative general procedure, *E*-hex-2-en-1-ol (1.0 g, 9.98 mmol) was reacted. The crude was purified via column chromatography on silica gel (*n*-Hep/EtOAc 3:1; R_f = 0.36 (1:1 *n*-Hep/EtOAc)) to yield *E*-**1k** as a colorless oil (1.07 g, 6.73 mmol, 159.19 g/mol, 67 %). Spectroscopic data are in agreement with literature precedent.[5]

^{1}H-NMR (400 MHz, $CDCl_3$): δ [ppm] = 7.35 (s, 1H, -NH-), 7.19 (s, 1H, -OH), 5.86-5.76 (m, 1H), 5.63-5.53 (m, 1H), 4.61 (d, *J* = 6.6 Hz, 2H), 2.05 (q, *J* = 7.0 Hz, 2H), 1.49-1.37 (m, 2H), 0.92 (t, *J* = 7.4 Hz, 3H). **^{13}C-NMR** (100 MHz, $CDCl_3$): δ [ppm] = 159.4, 137.3, 123.5, 67.0, 34.3, 22.0, 13.6. **FT-IR** (ATR) ν [cm^{-1}] = 3300 (w), 2958 (w), 1707 (m), 1457 (w), 1260 (m), 1110 (s).

_Z_-Hex-2-en-1-yl hydroxycarbamate (_Z_-1k)

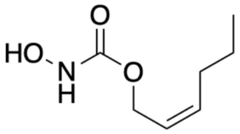

Z **-1k**

According to the representative general procedure, *Z*-hex-2-en-1-ol (1.0 g, 9.98 mmol) was reacted. The crude was purified via column chromatography on silica gel (3:1 *n*-Hep/EtOAc; R_f = 0.2 (1:1 *n*-Hep/EtOAc)) to yield

Z-**1k** as a colorless oil (1.28 g, 8.75 mmol, 159.19 g/mol, 88 %). Spectroscopic data are in agreement with literature precedent.[5]

^{1}H-NMR (400 MHz, $CDCl_3$): δ [ppm] = 7.23 (s, 1H, -NH-), 6.79 (s, 1H, -OH), 5.72-5.62 (m, 1H), 5.58-5.49 (m, 1H), 4.71 (d, *J* = 6.9 Hz, 2H), 2.08 (q, *J* = 7.3 Hz, 2H), 1.47-1.34 (m, 2H), 0.91 (t, *J* = 7.4 Hz, 3H). **^{13}C-NMR** (100 MHz, $CDCl_3$): δ [ppm] = 159.5, 135.9, 122.9, 62.0, 29.5, 22.5, 13.6. **FT-IR** (ATR) ν [cm^{-1}] = 3294 (w), 2960 (w), 1706 (m), 1458 (w), 1259 (m), 1108 (s).

4-Methylenehex-5-en-1-yl hydroxycarbamate (1l)

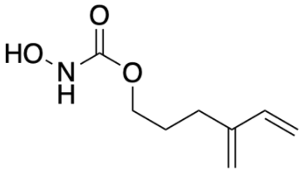

1l

4-Methylenehex-5-en-1-ol was prepared from myrcene according to literature precedent.[10] According to the representative general procedure, freshly prepared 4-methylenehex-5-en-1-ol (50 mg, 0.45 mmol) was reacted. The crude was purified via column chromatography on silica gel (5:1 *n*-Hep/EtOAc; R_f = 0.34 (1:1 *n*-Hep/EtOAc)) to yield **1l** as a colorless oil (37.4 mg, 0.22 mmol, 171.2 g/mol, 49 %). Spectroscopic data are in agreement with literature precedent.[15]

^{1}H-NMR (400 MHz, $CDCl_3$): δ [ppm] = 7.24 (s, 1H, -NH-), 6.79 (bs, 1H, -OH), 6.37 (dd, *J* = 17.6, 10.8 Hz, 1H), 5.21 (d, *J* = 17.6 Hz, 1H), 5.07 (d, *J* = 11.0 Hz, 1H), 5.02 (d, *J* = 19.2 Hz, 2H), 4.19, (t, *J* = 6.5 Hz, 2H), 2.31-2.25 (m, 2H), 1.8-1.9 (m, 2H). **^{13}C-NMR** (100 MHz, $CDCl_3$): δ [ppm] = 159.5, 145.0, 138.5, 116.3, 113.5, 66.0, 27.4, 27.2. **FT-IR** (ATR) ν [cm^{-1}] = 3279 (w), 2961 (vw), 1712 (m), 1455 (w), 1259 (m), 1112 (s).

***N*-hydroxy-2-(2-methylprop-1-en-1-yl)benzamide (1m)**

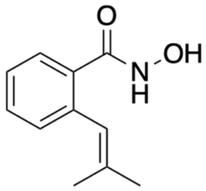

1m

A solution of methyl 2-bromobenzoate (1 g, 4.65 mmol, 1.0 eq.), 4,4,5,5-tetramethyl-2-(2-methylprop-1-en-1-yl)-1,3,2-dioxaborolane (1.27 g, 6.98 mmol, 1.5 eq.) and Cs_2CO_3 (3 g, 9.5 mmol, 2.0 eq.) in 11 mL dioxane/H_2O (4:1) was degassed with Argon for 10 min. Pd(dppf)Cl_2·DCM (380 mg, 0.47 mmol, 0.1 eq.) was added and the reaction mixture was heated to 100 °C for 3 h. The mixture was cooled down and filtered through celite using EtOAc. The filtrate was washed with brine, dried over Na_2SO_4, filtered and the solvent was removed under reduced pressure. The crude was purified via column chromatography on silica gel (10:1 *n*-Hep/EtOAc; R_f = 0.62 (5:1 *n*-Hep/EtOAc)) to yield methyl 2-(2-methylprop-1-en-1-yl)benzoate (799 mg, 4.2 mmol, 190.24 g/mol, 90 %).

^{1}H-NMR (400 MHz, $CDCl_3$): δ [ppm] = 7.92-7.87 (m, 1H), 7.45 (td, *J* = 7.6, 1.4 Hz, 1H), 7.26 (t, *J* = 7.4 Hz, 2H), 6.66 (s, 1H), 3.87 (s, 3H), 1.93 (d, *J* = 1.2 Hz, 3H), 1.71 (d, *J* = 1.2 Hz, 3H). **^{13}C-NMR** (100 MHz, $CDCl_3$): δ [ppm] = 167.9, 140.1, 134.8, 131.4, 131.3, 130.2, 129.3, 126.0, 124.7, 51.9, 26.3, 19.3.

To a solution of methyl 2-(2-methylprop-1-en-1-yl)benzoate (546 mg, 2.87 mmol) in 40 mL MeOH was added 22 mL 1M NaOH. The reaction mixture was stirred overnight and was then slightly acidified with diluted HCl. The aqueous phase was extracted 3x with EtOAc. The combined organic phases were dried over Na_2SO_4,

filtered and the solvent was removed under reduced pressure. The crude was purified via silica pad filtration to yield 2-(2-Methylprop-1-en-1-yl)benzoic acid (503.9 mg, 2.64 mmol, 191.23 g/mol, 92 %).

^{1}H-NMR (400 MHz, $CDCl_3$): δ [ppm] = 8.05 (d, *J* = 7.8 Hz, 1H), 7.53-7.44 (m, 1H), 7.35-7.27 (m, 2H), 6.71 (s, 1H), 1.93 (s, 3H), 1.73 (s, 3H). **^{13}C-NMR** (100 MHz, $CDCl_3$): δ [ppm] = 140.7, 140.1, 135.3, 132.2, 131.5, 131.2, 126.2, 124.7, 26.3, 19.4.

To a solution of 2-(2-Methylprop-1-en-1-yl)benzoic acid (200 mg, 1.05 mmol, 1.0 eq.) and a catalytic amount of DMF in 5 mL DCM was added oxalyl chloride (400 mg, 3.15 mmol, 3.0 eq.) at 0 °C. The solvent was removed after 3 h and the residue was again dissolved in 1 mL DCM. $NaHCO_3$ (198 mg, 2.36 mmol, 2.25 eq.) was dissolved in 1.2 mL H_2O and cooled to 0 °C. Hydroxylamine hydrochloride (91 mg, 1.31 mmol, 1.25 eq.) was added and the mixture was stirred for 30 min. before the solution of the residue in DCM was added. The mixture was stirred at 0 °C for 30 min. and was then allowed to warm to room temperature. After 2 h, EtOAc and H_2O was added to the reaction mixture and the aqueous phase was extracted 2x with EtOAc. The combined organic phases were washed with brine, dried over Na_2SO_4, filtered and the solvent was removed under reduced pressure. The crude was purified via column chromatography on silica gel (5:1 → 1:1 *n*-Hep/EtOAc; R_f = 0.18 (1:1 *n*-Hep/EtOAc)) to yield **1m** as a white solid (126.1 mg, 0.66 mmol, 191.23 g/mol, 63 %).

^{1}H-NMR (400 MHz, $CDCl_3$): δ [ppm] = 8.94 (s, 2H), 7.81 (d, *J* = 5.4 Hz, 1H), 7.46 (t, *J* = 7.5 Hz, 1H), 7.33 (t, *J* = 7.4 Hz, 1H), 7.22 (d, *J* = 7.6 Hz, 1H), 6.44 (s, 1H), 1.95 (s, 3H), 1.75 (s, 3H).**^{13}C-NMR** (100 MHz, $CDCl_3$): δ [ppm] = 139.3, 136.6, 131.0, 131.0, 130.8, 129.1, 126.9, 123.1, 26.1, 19.4. **FT-IR** (ATR) ν [cm^{-1}] = 3303 (w), 2906 (vw), 1633 (m), 1514 (m), 1019 (m). **Melting point**: 129.9 °C**. HRMS** (ESI): *m/z* [M+H^+] calcd for $C_{11}H_{13}NO_2$: 192.1019; found: 192.1030.

Enzyme mediated intramolecular nitroso-ene reactions

General procedure with GOx/HRP: To a 10 mM solution of substrate in 7 mL phosphate buffer (100 mM, pH 7.0), if necessary including 10 vol% of desired cosolvent (dioxane or EtOAc), was added 70 U horseradish peroxidase (1.5 nmol) and 70 U glucose oxidase (0.5 nmol). D-glucose (50 mM) was added and the reaction was incubated at 25 °C. After full conversion of starting material (followed by TLC), the reaction mixture was extracted 3x with EtOAc. The combined organic phases were dried over Na_2SO_4, filtered and the solvent was removed under reduced pressure. The crude was purified via silica pad filtration or flash column chromatography on silica gel.

General procedure with laccase: To a 5 mM solution of substrate in 7 mL phosphate buffer (100 mM, pH 7.0), if necessary including 10 vol% of desired cosolvent (dioxane or MeCN), was added 1 U (3.7 uL, 2.7 nmol) laccase CotA (*Bacillus lichenformis*). The reaction was incubated at 25 °C. After full conversion of starting material (followed by TLC), the reaction mixture was extracted 3x with EtOAc. The combined organic phases were dried over Na_2SO_4, filtered and the solvent was removed under reduced pressure. The yield was determined with quantitative ^{1}H-NMR measurements using dimethylsulfone as internal standard.

3-Hydroxy-4-(prop-1-en-2-yl)oxazolidin-2-one (2a)

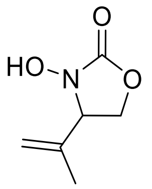

2a

According to the general procedure with GOx/HRP, **1a** was reacted and full conversion was observed after 2 h. The crude was purified via silica pad filtration with EtOAc to yield **2a** as colorless crystals (9.7 mg, 0.068 mmol, 143.14 g/mol, 97 %).

Upon upscaling to 49 mL reaction medium under otherwise identical concentrations, **2a** was obtained as colorless crystals in 92% yield (66 mg, 0.46 mmol).

According to the general procedure with laccase, **1a** was reacted and full conversion was observed after 7 h. The procedure yielded 74 % of the product **2a** (^{1}H-NMR yield).

Spectroscopic data are in agreement with literature precedent.[6]

^{1}H-NMR (400 MHz, $CDCl_3$): δ 8.29 (bs, 1H), 5.10 (dd, *J* = 8.4, 7.1 Hz, 2H), 4.49-4.30 (m, 2H), 4.08-3.97 (m, 1H), 1.81-1.76 (m, 3H). **^{13}C-NMR** (100 MHz, $CDCl_3$): δ 160.9, 139.0, 117.0, 65.7, 64.8, 16.9. **FT-IR** (ATR) ν [cm^{-1}] = 3236 (w), 2923 (w), 1743 (s), 1215 (m), 1095 (m). **Melting point**: 73.0 °C.

3-Hydroxy-4-(prop-1-en-2-yl-d$_5$)oxazolidin-2-one (2a-d$_5$)

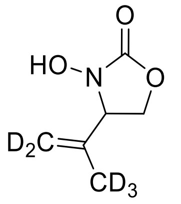

2a-d$_5$

According to the general procedure with GOx/HRP, **1a-d₆** was reacted and full conversion was observed after 2 h. The crude was purified via silica pad filtration with EtOAc to yield **2a-d₅** as colorless crystals (9.4 mg, 0.063 mmol, 148.17 g/mol, 91 %, D_5 ≥ 99 %).

According to the general procedure with laccase, **1a-d₅** was reacted and full conversion was observed after 6.5 h. The procedure yielded 65 % of the product **2a-d₅** (^{1}H-NMR yield).

^{1}H-NMR (400 MHz, $CDCl_3$): δ [ppm] = 8.26 (bs, 1H, -OH), 4.46-4.36 (m, 2H), 4.07-3.97 (m, 1H). **FT-IR** (ATR) ν [cm^{-1}] = 3183 (w), 2924 (vw), 1748 (s), 1103 (s), 1007 (m). **Melting point**: 67.5 °C. **HRMS** (ESI): *m/z* [M+Na$^+$] calcd for $C_6H_4D_5NO_3$: 171.0788; found: 171.0801.

3-Hydroxy-4-(prop-1-en-2-yl-1,1-d₂)oxazolidin-2-one (2a-d₂)

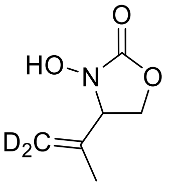

2a-d₂

According to the general procedure with GOx/HRP, **1a**-*E*-**d₃** was reacted and full conversion was observed after 2 h. The crude was purified via silica pad filtration with EtOAc to yield **2a-d₂** (9.5 mg, 0.065 mmol, 145.15 g/mol, 93 %, D_2 ≥ 95 %).

According to the general procedure with laccase, **1a-d₂** was reacted and full conversion was observed after 6 h. The procedure yielded 68 % of the product **2a-d₂** (^{1}H-NMR yield).

^{1}H-NMR (400 MHz, $CDCl_3$): δ [ppm] = 8.17 (s, 1H), 4.46-4.37 (m, 2H), 4.07-3.97 (m, 1H), 1.78 (s, 3H). **FT-IR** (ATR) ν [cm^{-1}] = 3259 (vw), 2917 (vw), 1745 (s), 1212 (m), 1088 (m). **HRMS** (ESI): *m/z* [M+H$^+$] calcd for $C_6H_7D_2NO_3$: 146.0781; found: 146.0784.

3-Hydroxy-4-(prop-1-en-2-yl-3,3,3-d₃)oxazolidin-2-one (2a-d₃)

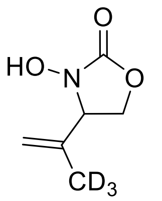

2a-d₃

According to the general procedure with GOx/HRP, **1a**-*Z*-**d₃** was reacted and full conversion was observed after 2 h. The crude was purified via silica pad filtration with EtOAc to yield **2a-d₃** (9.1 mg, 0.062 mmol, 146.15 g/mol, 89 %, D_3 ≥ 95 %).

According to the general procedure with laccase, **1a-d₃** was reacted and full conversion was observed after 6 h. The procedure yielded 67 % of the product **2a-d₃** (^{1}H-NMR yield).

^{1}H-NMR (400 MHz, $CDCl_3$): δ [ppm] = 8.25 (s, 1H), 5.13 (s, 1H), 5.10 (d, *J* = 1.0 Hz, 1H), 4.45-4.37 (m, 2H), 4.05-3.98 (m, 1H). **FT-IR** (ATR) ν [cm^{-1}] = 3259 (vw), 2917 (vw), 1747 (s), 1212 (m), 1089 (m). **HRMS** (ESI): *m/z* [M+Na$^+$] calcd for $C_6H_6D_3NO_3$: 147.0844; found: 146.0842.

1-Hydroxy-5-(prop-1-en-2-yl)pyrrolidin-2-one (2b)

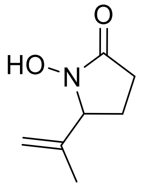

2b

According to the general procedure with GOx/HRP, **1b** was reacted in phosphate buffer containing 10 vol% dioxane and full conversion was observed after 3 h. The crude was purified via column chromatography (DCM + 5 % MeOH + 1 % TEA; R_f = 0.34 (DCM + 5 % MeOH)) to yield **2b** as white solid (7.6 mg, 0.054 mmol, 141.17 g/mol, 77 %). Spectroscopic data are in agreement with literature precedent.[16]

^{1}H-NMR (400 MHz, $CDCl_3$): δ [ppm] = 9.44 (bs, 1H, -OH), 5.00 (d, *J* = 15.5 Hz, 2H), 4.28 (dd, *J* = 8.4, 5.6 Hz, 1H), 2.51-2.31 (m, 2H), 2.29-2.16 (m, 1H), 1.86-1.75 (m, 1H), 1.73 (s, 3H). **^{13}C-NMR** (100 MHz, $CDCl_3$): δ [ppm] = 142.4, 114.2, 65.0, 27.2, 21.5, 16.9. **FT-IR** (ATR) ν [cm^{-1}] = 3085 (vw), 2497 (w), 1668 (s), 1456 (m), 926 (s). **Melting point**: 91.3 °C.

3-Hydroxy-4-vinyloxazolidin-2-one (2c)

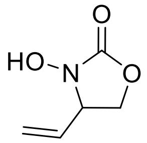

2c

According to the general procedure with GOx/HRP, **1c** was reacted and full conversion was observed after 2 h. The crude was purified via column chromatography (5:1 *n*-Hep/EtOAc; R_f = 0.23 (1:1 *n*-Hep/EtOAc)) to yield **2c** as a white solid with a diastereomeric ratio of 68 % (4.8 mg, 0.037 mmol, 129.12 g/mol, 53 %). Spectroscopic data are in agreement with literature precedent.[6]

^{1}H-NMR (400 MHz, $CDCl_3$): δ [ppm] = 6.78 (bs, 1H, -OH), 5.81 (ddd, *J* = 17.2, 10.2, 7.8 Hz, 1H), 5.47 (dd, *J* = 23.3, 13.6 Hz, 2H), 4.43 (t, *J* = 8.2 Hz, 1H), 4.33 (dd, *J* = 16.7, 8.2 Hz, H-1), 3.99 (t, *J* = 8.2 Hz, 1H). **^{13}C-NMR** (100 MHz, $CDCl_3$): δ [ppm] = 160.5, 132.3, 122.3, 66.3, 62.8. **FT-IR** (ATR) ν [cm^{-1}] = 3244 (w), 2922 (vw), 1726 (s), 1471 (w), 1090 (m). **Melting point**: 55.0 °C.

3-Hydroxy-4-methyl-4-(prop-1-en-2-yl)oxazolidin-2-one (2d)

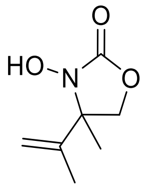

2d

According to the general procedure with GOx/HRP, **1d** was reacted and full conversion was observed after 2 h. The crude was purified via silica pad filtration with EtOAc to yield **2d** as colorless oil (9.1 mg, 0.064 mmol, 143.14 g/mol, 91 %).

^{1}H-NMR (400 MHz, $CDCl_3$): δ [ppm] = 7.65 (bs, 1H, -OH), 5.91 (dd, *J* = 17.5, 10.8 Hz, 1H), 5.45-5.19 (m, 2H), 4.12 (d, *J* = 8.4 Hz, 1H), 4.06 (d, *J* = 8.4 Hz, 1H), 1.47 (d, *J* = 5.5 Hz, 3H). **^{13}C-NMR** (100 MHz, $CDCl_3$): δ [ppm] = 159.7, 136.6, 118.1, 72.6, 64.1, 18.9. **FT-IR** (ATR) ν [cm^{-1}] = 3260 (w), 2976 (vw), 1746 (s), 1101 (m), 990 (m). **HRMS** (ESI): *m/z* [M+H$^+$] calcd for $C_6H_9NO_3$: 144.0656; found: 144.0657.

4-(Cyclohex-1-en-1-yl)-3-hydroxyoxazolidin-2-one (2e)

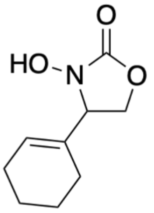

2e

According to the general procedure with GOx/HRP, **1e** was reacted in phosphate buffer containing 10 vol% EtOAc under vigorous stirring. Full conversion was observed after 2 h. The crude was purified via column chromatography (4:1 *n*-Hep/EtOAc; R_f = 0.35 (1:1 *n*-Hep/EtOAc)) to yield **2e** as colorless crystals (9.9 mg, 0.054 mmol, 183.21 g/mol, 77 %).

According to the general procedure with laccase, **1e** was reacted was reacted in phosphate buffer containing 10 vol% MeCN and full conversion was observed after 4 h. The procedure yielded 73 % of the product **2e** (^{1}H-NMR yield).

Spectroscopic data are in agreement with literature precedent.[9]

^{1}H-NMR (400 MHz, $CDCl_3$): δ [ppm] = 8.09 (bs, 1H, -OH), 5.83 (s, 1H), 4.38-4.28 (m, 2H), 4.08-3.97 (m, 1H), 2.14-1.86 (m, 4H), 1.72-1.50 (m, 4H). **^{13}C-NMR** (100 MHz, $CDCl_3$): δ [ppm] = 160.9, 131.6, 129.4, 65.7, 65.4, 25.1, 22.8, 22.2, 22.1. **FT-IR** (ATR) v [cm^{-1}] = 3252 (vw), 2927 (w), 1741 (s), 1096 (m), 1016 (m). **Melting point**: 90.1 °C.

3-Hydroxy-5-methyl-4-vinyloxazolidin-2-one (2f)

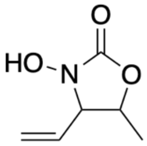

2f

According to the general procedure with GOx/HRP, **1f** was reacted in phosphate buffer containing 10 vol% EtOAc under vigorous stirring. Full conversion was observed after 5 h. The crude was purified via column chromatography (5:1 *n*-Hep/EtOAc; R_f = 0.29 & 0.21 (1:1 *n*-Hep/EtOAc)) to yield **2f** colorless oil with a diastereomeric ratio of 68:32 (5.2 mg, 0.036 mmol, 143.14 g/mol, 52 %).

According to the general procedure with laccase, **1f** was reacted in phosphate buffer containing 10 vol% MeCN and full conversion was observed after 18 h. The procedure yielded 30 % of the product **2f** with a diastereomeric ratio of 2:1 (^{1}H-NMR yield).

Spectroscopic data are in agreement with literature precedent.[9]

Major diastereomer

^{1}H-NMR (400 MHz, $CDCl_3$): δ [ppm] = 7.80 (bs, 1H, -OH), 5.87-5.71 (m, 1H), 5.53-5.38 (m, 2H), 4.75-4.65 (m, 1H), 4.33 (t, *J* = 8.0 Hz, 1H), 1.32 (d, *J* = 6.6 Hz, 3H). **^{13}C-NMR** (100 MHz, $CDCl_3$): δ [ppm] = 130.1, 122.9, 75.1, 65.8, 16.0.

Minor diastereomer (selected signals)

^{1}H-NMR (400 MHz, $CDCl_3$): δ [ppm] = 4.22 (dq, *J* = 9.3, 6.2 Hz, 1H), 3.92-3.81 (m, 1H), 1.44 (d, *J* = 6.2 Hz, 3H). **^{13}C-NMR** (100 MHz, $CDCl_3$): δ [ppm] = 132.4, 122.5, 73.4, 70.3, 17.8.

FT-IR (ATR) v [cm^{-1}] = 3280 (vw), 2936 (vw), 1748 (s), 1220 (m), 1057 m).

8R-1-Hydroxy-8-(prop-1-en-2-yl)-3-oxa-1-azaspiro[4.5]dec-6-en-2-one (2g)

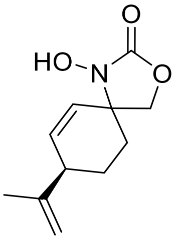

2g

According to the general procedure with GOx/HRP, **1g** was reacted in phosphate buffer containing 10 vol% EtOAc under vigorous stirring. Full conversion was observed after 4 h. The crude was purified via column chromatography (3:1 *n*-Hep/EtOAc; R_f = 0.44 (1:1 *n*-Hep/EtOAc)) to yield **2g** as a colorless oil with diastereomeric ratio of 92 % (14.2 mg, 0.068 mmol, 209.25 g/mol, 97 %).

Major diastereomer

^{1}H-NMR (400 MHz, $CDCl_3$): δ [ppm] = 8.03 (bs, 1H, -OH), 5.99 (dd, *J* = 10.0, 3.8 Hz, 1H), 5.67 (dd, *J* = 10.1, 1.5 Hz, 1H), 4.82 (d, *J* = 34.0 Hz, 2H), 4.11-3.99 (m, 2H), 2.68 (d, *J* = 4.9 Hz, 1H), 2.30-2.15 (m, 1H), 1.90 (ddd, *J* = 13.0, 8.0, 4.2 Hz, 1H), 1.76 (s, 3H), 1.78-1.68 (m, 1H), 1.64-1.53 (m, 1H). **^{13}C-NMR** (100 MHz, $CDCl_3$): δ [ppm] = 159.5, 145.7, 137.8, 126.2, 112.7, 71.7, 62.3, 41.3, 25.9, 23.4, 21.7.

Minor diastereomer (selected signals):

^{1}H-NMR (400 MHz, $CDCl_3$): δ [ppm] = 6.09 (d, *J* = 10.2 Hz, 1H), 5.59 (d, *J* = 9.3 Hz, 1H), 2.86-2.79 (m, 1H). **^{13}C-NMR** (100 MHz, $CDCl_3$): δ [ppm] = 147.2, 138.4, 126.6, 111.0, 71.5, 63.0, 42.5, 26.9, 25.2, 20.7. **FT-IR** (ATR) ν [cm^{-1}] = 3263 (vw), 2935 (vw), 1748 (s), 1451 (w), 1088 (m). **HRMS** (ESI): *m/z* [$M+H^+$] calcd for $C_{11}H_{15}NO_3$: 210.1125, found: 210.1128.

3-Hydroxy-4-(prop-1-en-2-yl)-1,3-oxazinan-2-one (2h)

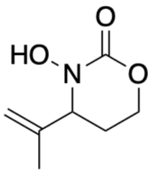

2h

According to the general procedure with GOx/HRP, **1h** was reacted in phosphate buffer containing 10 vol% EtOAc under vigorous stirring. Full conversion was observed after 2 h. The crude was purified via column chromatography (DCM + 2 % MeOH; R_f = 0.46 (DCM + 5 % MeOH)) to yield **2h** (6.4 mg, 0.041 mmol, 157.17 g/mol, 58 %).

According to the general procedure with laccase, **1h** was reacted in phosphate buffer containing 10 vol% MeCN and full conversion was observed after 4.5 h. The procedure yielded 45 % of the product **2h** (^{1}H-NMR yield).

Spectroscopic data are in agreement with literature precedent.[6]

^{1}H-NMR (400 MHz, $CDCl_3$): δ [ppm] = 8.34 (s, 1H), 5.13-5.08 (m, 2H), 4.31-4.16 (m, 3H), 2.27-2.19 (m, 1H), 2.04-1.97 (m, 1H), 1.76 (s, 1H). **^{13}C-NMR** (100 MHz, $CDCl_3$): δ [ppm] = 155.3, 141.2, 114.1, 63.9, 63.5, 27.1, 18.5. **FT-IR** (ATR) ν [cm^{-1}] = 3204 (vw), 2918 (vw), 1683 (s), 1425 (m), 1283 (m).

E-4-(But-1-en-1-yl)-3-hydroxyoxazolidin-2-one (2k)

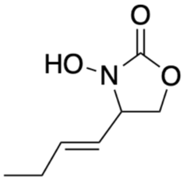

2k

According to the general procedure with GOx/HRP, *E*-**1k** was reacted in phosphate buffer containing 10 vol% EtOAc under vigorous stirring. Full conversion was observed after 2 h. The crude was purified via column chromatography (4:1 *n*-Hep/EtOAc; R_f = 0.34 (1:1 *n*-Hep/EtOAc)) to yield **2k** as a colorless oil with *E*/*Z* ratio of 75:25 (7.3 mg, 0.046 mmol, 157.17 g/mol, 66 %).

According to the general procedure with laccase, *E*-**1k** was reacted in phosphate buffer containing 10 vol% MeCN and full conversion was observed after 11.5 h. The procedure yielded 67 % of the product **2k** with an *E*/*Z* ratio of 69:31 (^{1}H-NMR yield).

Spectroscopic data are in agreement with literature precedent.[6]

E-Isomer

^{1}H-NMR (400 MHz, $CDCl_3$): δ [ppm] = 7.72 (bs, 1H, -OH), 5.94 (dt, *J* = 15.3, 6.3 Hz, 1H, 5.38 (ddt, *J* = 15.4, 8.3, 1.6 Hz, 1H), 4.39 (t, *J* = 8.1 Hz, 1H), 4.30 (dd, *J* = 16.8, 8.3 Hz, 1H), 3.95 (t, *J* = 8.1 Hz,1H), 2.26-2.04 (m, 2H), 1.02 (t, *J* = 7.4 Hz, 3H). **^{13}C-NMR** (100 MHz, $CDCl_3$): δ [ppm] = 159.4, 137.3, 123.5, 67.0, 34.3, 22.0, 13.6.

Z-Isomer (selected signals):

^{1}H-NMR (400 MHz, $CDCl_3$): δ [ppm] = 5.84 (dt, *J* = 10.7, 7.7 Hz, 1H), 4.72 (q, *J* = 9.1 Hz, 1H), 1.01 (t, *J* = 7.5 Hz, 3H). **^{13}C-NMR** (100 MHz, $CDCl_3$): δ [ppm] = 66.6, 22.5, 14.0. **FT-IR** (ATR) ν [cm^{-1}] = 3265 (vw), 2965 (vw), 1754 (s), 1462 (w), 1210 (w), 1081 (m).

2-Hydroxy-3-(2-hydroxypropan-2-yl)isoindolin-1-one (7)

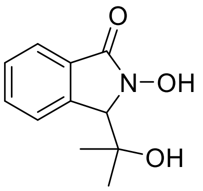

7

According to the general procedure with GOx/HRP, **1m** was reacted in phosphate buffer containing 10 % EtOAc under vigorous stirring. The reaction mixture was extracted after 30 h. The crude of four reactions was combined and purified via column chromatography (1:1 *n*-Hep/EtOAc; R_f = 0.11 (1:1 *n*-Hep/EtOAc)) to yield **7** as a white solid (7.7 mg, 0.041 mmol, 207.23 g/mol, 13 %) and reisolate **1m** (33.2 mg, 0.174 mmol, 62 %).

^{1}H-NMR (400 MHz, CD_3OD): δ [ppm] = 7.86 (d, *J* = 7.7 Hz, 1H), 7.74 (d, *J* = 7.5 Hz, 1H), 7.61 (td, *J* = 7.6, 1.2 Hz, 1H), 7.51 (t, *J* = 7.5 Hz, 1H), 5.02 (s, 1H), 1.62 (s, 3H), 0.78 (s, 3H). **^{13}C-NMR** (100 MHz, CD_3OD): δ [ppm] = 168.4, 142.0, 133.0, 131.5, 129.6, 126.9, 123.6, 84.0, 67.5, 24.6, 19.2. **HRMS** (ESI): *m/z* [M+Na$^+$] calcd for $C_{11}H_{13}NO_3$: 230.0787, found: 230.0789.

GOx/HRP mediated nitroso-Diels-Alder reactions

3,10-Dioxa-1-azabicyclo[5.3.1]undec-7-en-2-one (6)

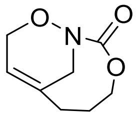

6

According to the general procedure with GOx/HRP, **1l** was reacted and full conversion was observed after 4 h. The crude was purified via column chromatography (3:1 *n*-Hep/EtOAc: R_f = 0.45 (1:1 *n*-Hep/EtOAc)) to yield **6** as colorless crystals (4.3 mg, 0.026 mmol, 169.18 g/mol, 36 %). Spectroscopic data are in agreement with literature precedent.[17]

^{1}H-NMR (400 MHz, $CDCl_3$): δ [ppm] = 5.79-5.74 (m, 1H), 4.73 (dd, *J* = 14.3, 5.5 Hz, 1H), 4.44-4.33 (m, 2H), 2.20 (dd, *J* = 15.0, 2.0 Hz, 1H), 4.15-4.04 (m, 1H), 3.49 (d, *J* = 15.0 Hz, 1H), 2.56-2.39 (m, 1H), 2.35-2.16 (m, 2H), 1.97-1.84 (m, 1H). **^{13}C-NMR** (100 MHz, $CDCl_3$): δ [ppm] = 164.2, 142.1, 123.0, 71.8, 67.3, 52.6, 31.8, 27.4. **FT-IR** (ATR) ν [cm^{-1}] = 2928 (vw), 1742 (s), 1443 (w), 1159 (s), 1050 (s). **Melting point**: 38.0 °C.

Enzyme mediated intermolecular nitroso-ene reaction

Benzyl (2,3-dimethylbut-3-en-2-yl)(hydroxy)carbamate (5)

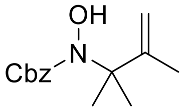

5

GOx/HRP-System: To a 10 mM solution of **3** in 7 mL phosphate buffer (100 mM, pH 7.0) including 10 vol% of EtOAc was added 2,3-dimethylbut-2-ene. 70 U horseradish peroxidase and 70 U glucose oxidase (*Aspergillus Niger*). D-glucose (50 mM) was added, and the reaction was incubated at 25 °C under vigorous stirring. After full conversion of starting material (followed by TLC), the reaction mixture was extracted 3 x with EtOAc. The combined organic phases were dried over Na_2SO_4, filtered and the solvent was removed under reduced pressure. The crude was purified via flash column chromatography on silica gel (12:1 *n*-Hep/EtOAc; R_f = 0.68 (1:1 *n*-Hep/EtOAc)) to afford **5**.

Table S1. Screening of 2,3-dimethylbut-2-ene loading in the nitroso-ene reaction of **3** using GOx/HRP.

entry	amount of 2,3-dimethylbut-2-ene	yield [%][a]
1	1.0 eq.	30
2	1.5 eq.	51
3	5.0 eq.	55
4	as cosolvent[b]	90

[a] isolated yield. [b] 10 vol %; instead of EtOAc.

Laccase-System: To a 5 mM solution of **3** in 7 mL phosphate buffer (100 mM, pH 7.0) including 10 vol% 2,3-dimethylbut-2-ene was added 1 U laccase CotA (*Bacillus licheniformis*) and the reaction was incubated at 25 °C. After full conversion of starting material (followed by TLC), the reaction mixture was extracted 3 x with EtOAc. The combined organic phases were dried over Na_2SO_4, filtered and the solvent was removed under reduced pressure. The procedure yielded 53 % of the product **5** (^{1}H-NMR yield).

Spectroscopic data are in agreement with literature precedent.[6]

^{1}H-NMR (400 MHz, $CDCl_3$): δ [ppm] = 7.37-7.30 (m, 5H), 6.23 (bs, 1H), 5.17 (s, 2H), 4.85 (s, 1H), 4.77 (s, 1H), 1.75 (s, 3H), 1.48 (s, 6H). **^{13}C-NMR** (100 MHz, CDCl3): δ [ppm] = 158.1, 149.5, 135.8, 128.5, 128.3, 128.2, 109.4, 68.0, 66.0, 25.4, 19.1. **FT-IR** (ATR) ν [cm^{-1}] = 3298 (vw), 2925 (vw), 1681 (m), 1326 (m), 1096 (m).

Variability & Turnover Numbers

Table S2. Substrate scope and total turnover numbers of HRP and CotA-mediated C-N bond forming reactions.

Entry	Substrate	Product	with HRP/GOx		with CotA	
			yield [a]	TTN [b]	yield [c]	TTN [b]
1	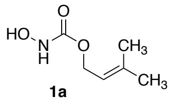 1a	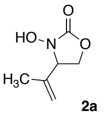 2a	97% ± 2%	43526	74% ± 0%	9739
2	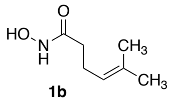 1b	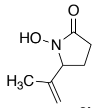 2b	74% ± 4%	32917		
3	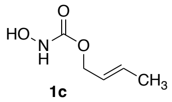 1c	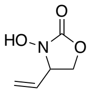 2c	54% ± 1%	23814		
4	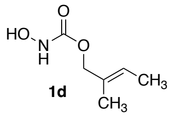 1d	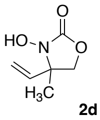 2d	94% ± 3%	42147	91% ± 5%	12135
5	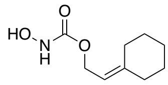 1e	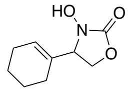 2e	83% ± 5%	37115	72% ± 1%	9611
6	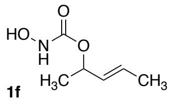 1f	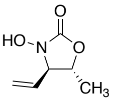 2f	56% ± 3%	24840	35% ± 5%	4782
7	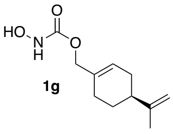 1g	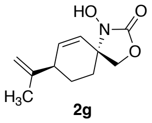 2g	89% ± 8%	39968		
8	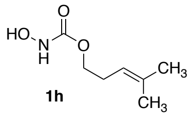 1h	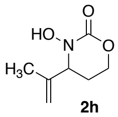 2h	57% ± 1%	25545	47% ± 2%	
9	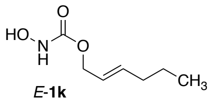 *E*-1k	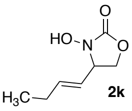 2k	75% ± 9%	33718	61% ± 6%	8061
10	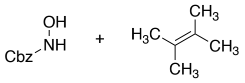 3 + 4	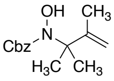 5	90% ± 8%	40224	53% ± 7%	7072
11	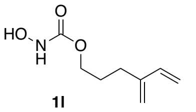 1l	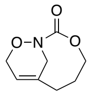 6	33% ± 3%	14968		
12	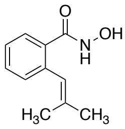 1m	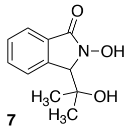 7	19% ± 2%	8205		

Laccase Screening

General procedure for laccase screenings: To a 10 mM solution of **1a** in 7 mL phosphate buffer (pH 7.0) or acetate buffer (100mM, pH 5.0), 1U laccase (Lccβ from *Trametes versicolor*, Mrl2 from *Moniliophthora roreri*, CotA from *Bacillus licheniformis*, Ssl1 from *Streptomyces sviceus* and Mtl from *Myceliophthora thermophila*) was added. The reaction was incubated at 25 °C. Full conversion of starting material was followed by TLC, the reaction mixture was extracted 3 x with EtOAc. The combined organic phases were dried over Na_2SO_4, filtered and the solvent was removed under reduced pressure. The yield was determined with quantitative ^{1}H-NMR measurements using dimethylsulfone as internal standard.

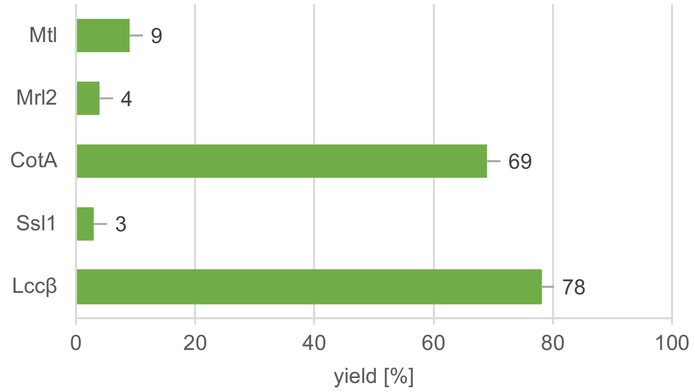

Figure S1. Screening of different laccases in the nitroso-ene reaction of **1a** using 1 U of each laccase. The reaction was stopped after 6h.

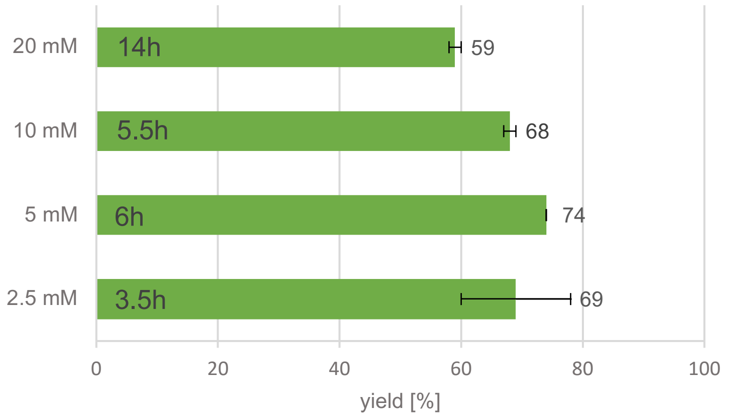

Figure S2. Screening of different substrate loadings in the nitroso-ene reaction of **1a** using 1 U laccase CotA. Times indicate the duration to reach full conversion of **1a** under given conditions.

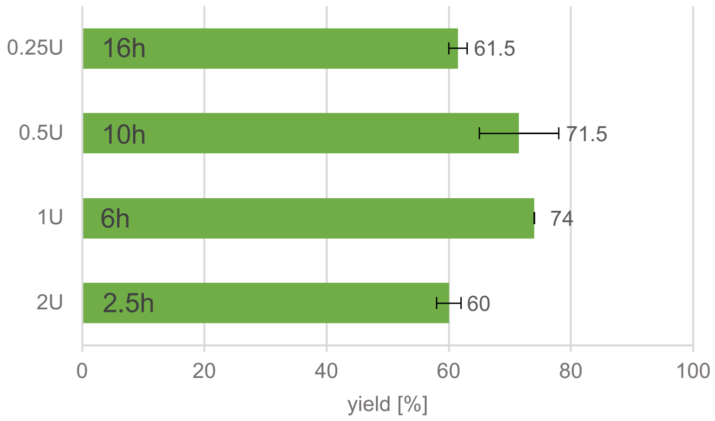

Figure S3. Screening of different enzyme loadings in the nitroso-ene reaction of **1a** using laccase CotA. Times indicate the duration to reach full conversion of **1a** under given conditions.

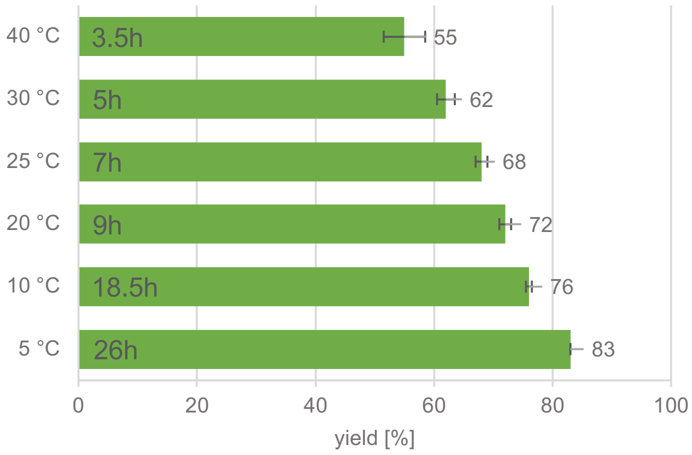

Figure S4. Screening of different temperatures in the nitroso-ene reaction of **1a** using 1 U laccase CotA. Times indicate the duration to reach full conversion of **1a** under given conditions.

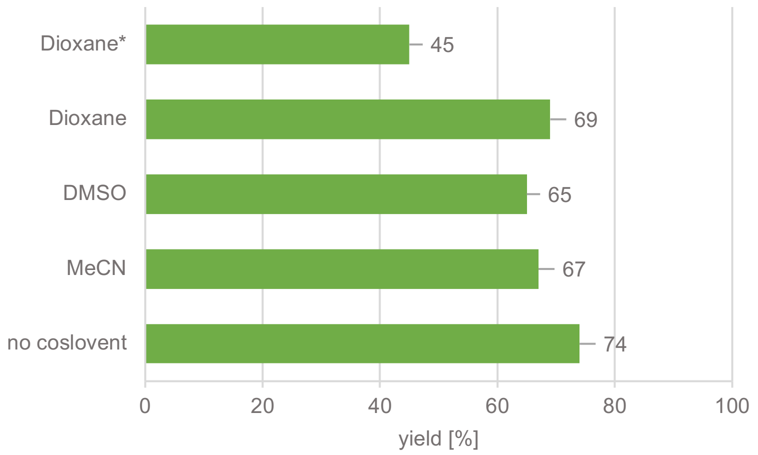

Figure S5. Screening of different cosolvents (10 vol%) in the nitroso-ene reaction of **1a** using 1 U laccase CotA. Full conversion was reached after 7 h for each reaction. [*] 40 vol%, stopped after 7 h.

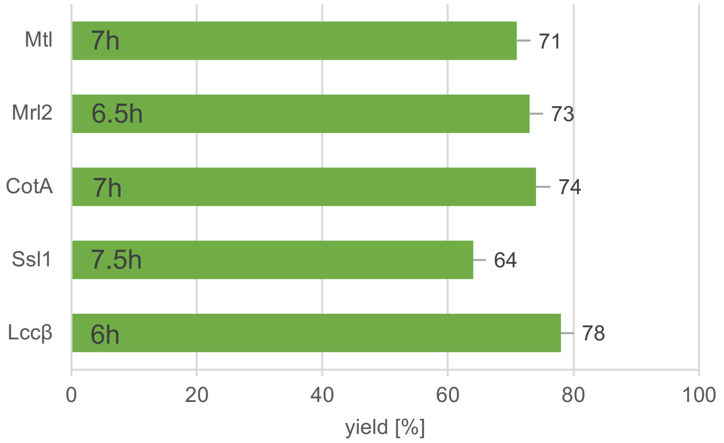

Figure S6. Screening of different laccases in the nitroso-ene reaction of **1a** using laccase (1U Lccβ, 10U Mrl2, 1U CotA, 20U Ssl1, 5U Mtl). Times indicate the duration to reach full conversion of **1a** under given conditions.

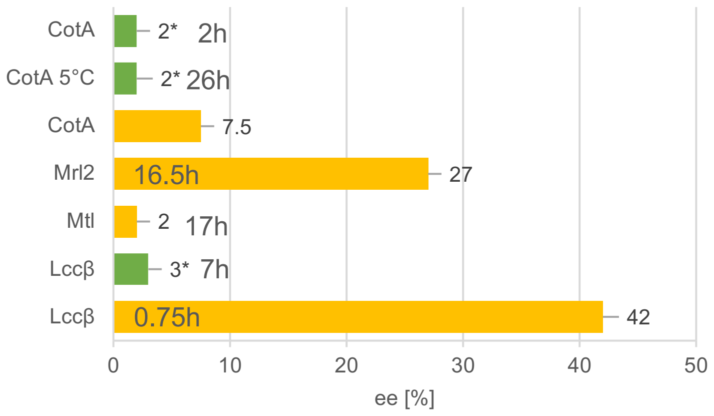

Figure S7. Stereoselectivity in acidic buffer solution (NaOAc buffer pH 5.0, 100mM, yellow) in the nitroso-ene reaction of **1a** using laccase (1 U Lccβ, 5 U Mrl2, 2 U CotA, 20 U Ssl1, 10 U Mtl) and corresponding reaction times. CotA did not show full conversion and the reaction was stopped after 24h. [*] In phosphate buffer (100 mM, pH 7.0, green).

Peroxidase Screening

General procedure for peroxidase screenings: To a 10 mM solution of **1a** in 7 mL phosphate buffer (100 mM, pH 7.0) or citrate buffer (100 mM, pH 5.5), 70 U peroxidase and 70 U glucose oxidase (*Aspergillus niger*) was added. D-glucose (50 mM) was added, and the reaction was incubated at 25 °C. The conversion was followed by TLC. After full conversion, the reaction mixture was extracted 3 x with EtOAc. The combined organic phases were dried over Na_2SO_4, filtered and the solvent was removed under reduced pressure. The crude was purified via silica pad filtration or flash column chromatography on silica gel. Reactions with lactoperoxidase and chloroperoxidase were performed in citrate buffer (100 mM, pH 5.5).

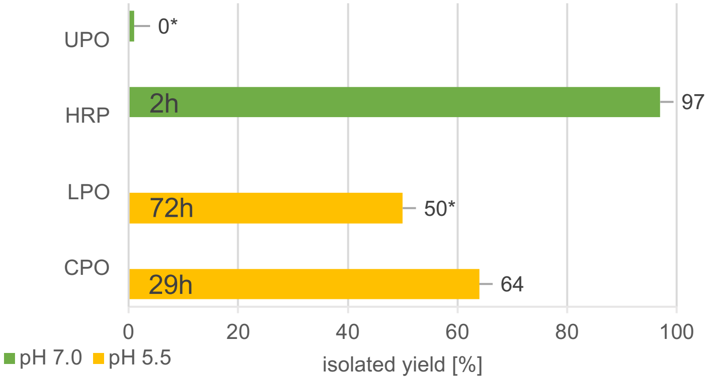

Figure S8. Screening of different peroxidases in the nitroso-ene reaction of **1a** using the GOx/HRP-system. [*] Conversion; yield not determined.

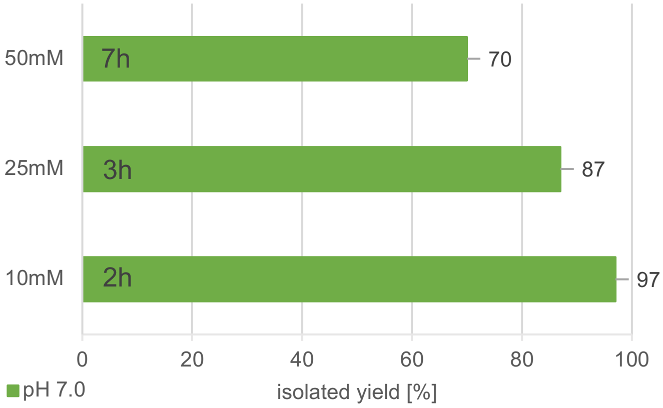

Figure S9. Screening of different substrate loadings in the nitroso-ene reaction of **1a** using the GOx/HRP-system.

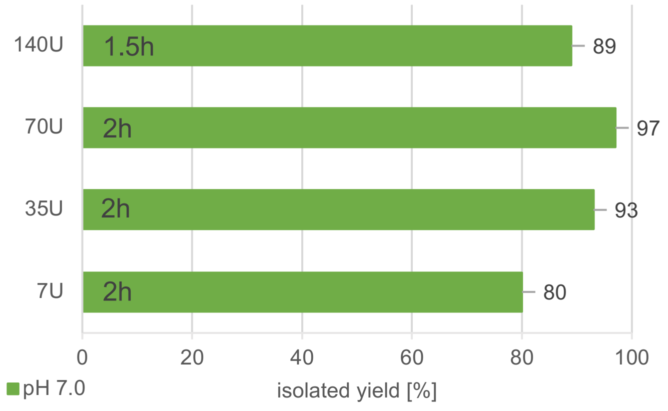

Figure S10. Screening of different GOx loadings in the nitroso-ene reaction of **1a** using the GOx/HRP-system.

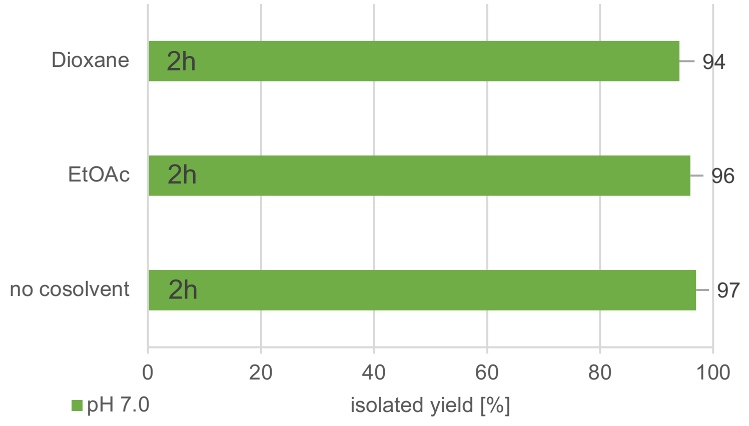

Figure S11. Screening of different cosolvents (10 vol%) in the nitroso-ene reaction of **1a** using the GOx/HRP-system.

Time course analysis

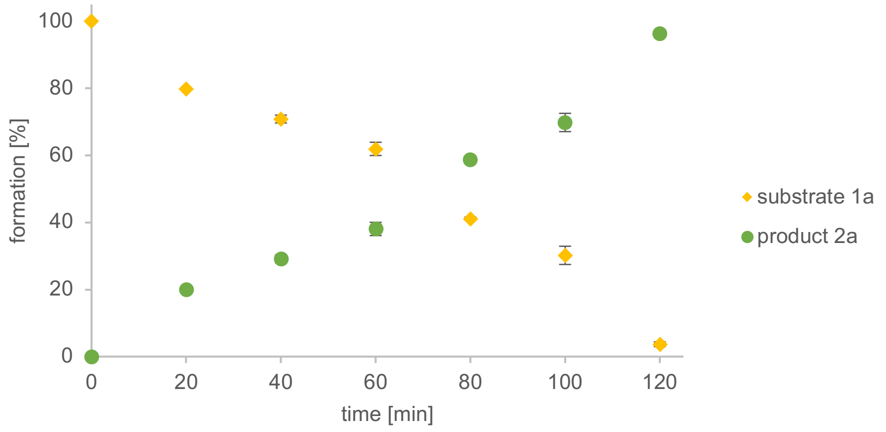

Figure S12. Formation of product **2a** in the nitroso-ene reaction of **1a** using the GOx/HRP-system.

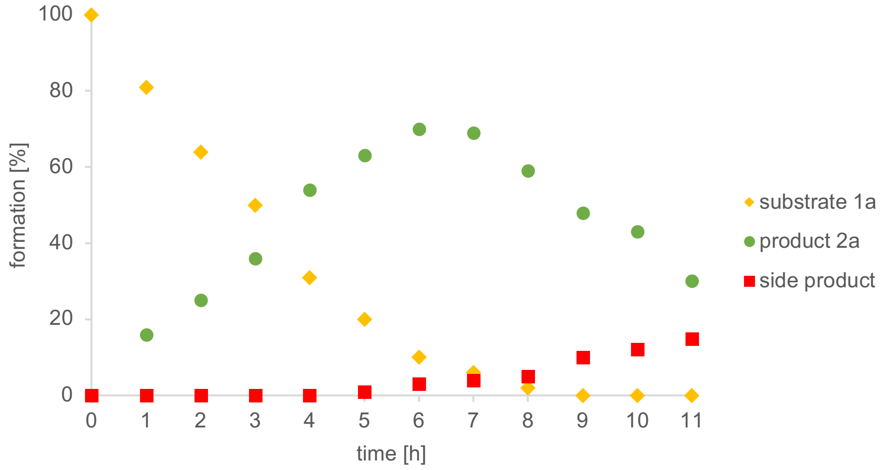

Figure S13. Formation of product **2a** and side product in the nitroso-ene reaction of **1a** using 1 U laccase CotA.

Michaelis-Menten Kinetics

To a solution of **1a** (concentrations: 2.5 mM, 5 mM, 10 mM, 20 mM, 40 mM, 60 mM, 80 mM) in 7 mL phosphate buffer (100 mM, pH 7.0), 1 U laccase CotA (*Bacillus licheniformis*) was added. The reaction was incubated at 25 °C and stopped at different times (30, 60, 90,120 min). The reaction mixture was extracted 3 x with EtOAc. The combined organic phases were dried over Na_2SO_4, filtered through a silica pad and the solvent was removed under reduced pressure. The conversion was determined by ^{1}H-NMR analysis, from which the KIE was calculated. Both reaction sets were performed twice under the same conditions.

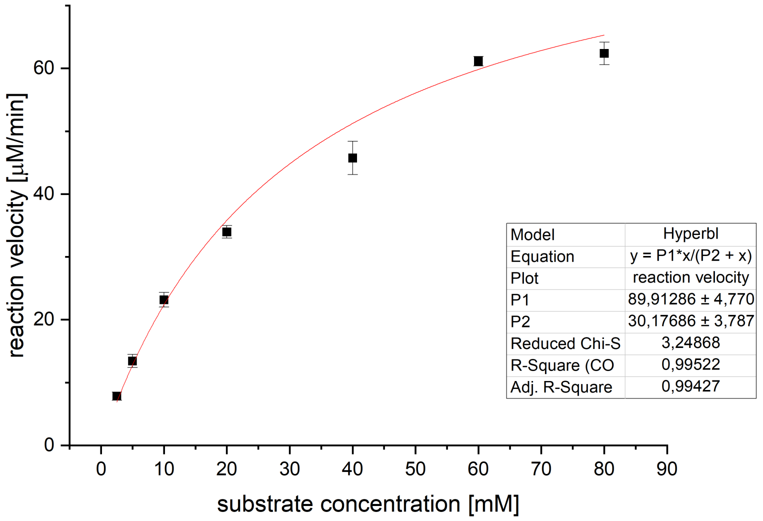

Figure S14. Michaelis Menten plot with P1 = V_{max} and P2 = K_m for the nitroso-ene reaction fo **1a** using laccase CotA.

Kinetic isotope effect studies

Kinetic isotope effect studies in the GOx/HRP catalyzed nitroso-ene reaction

Two reaction sets were run with **1a** and **1a-d$_6$** to calculate the kinetic isotope effect (KIE). To a 10 mM solution of substrate in 7 mL phosphate buffer (100 mM, pH 7.0) was added 3.5 U horseradish peroxidase and 3.5 U glucose oxidase (from *Aspergillus niger*). D-glucose (6.25 mM) was added, and the reaction was incubated at 25 °C. The reaction was stopped at different times (5, 10, 15, 20, 25, 30 min) and worked up as followed. The reaction mixture was extracted 3 x with EtOAc. The combined organic phases were dried over Na_2SO_4, filtered through a silica pad and the solvent was removed under reduced pressure. The conversion was determined by ^{1}H-NMR analysis, from which the KIE was calculated. Both reaction sets were performed twice under the same conditions. The overall average KIE was determined as inverse β secondary kinetic isotope effect **k_H/k_D = 0.79 ± 0.04**.

Table S3. Results of kinetic measurements of **1a** and **1a-d$_6$** using the GOx/HRP-system.

entry	time [min]	conversion [%][a]				k_H/k_D		
		**1a**	**1a**[b]	**1a-d$_6$**	**1a-d$_6$** [b]	1st set	2nd set	averaged
1	5	4.0	3.8	6.9	5.8	0.57	0.66	0.61 ± 0.05
2	10	7.6	8.4	9.2	10.9	0.82	0.77	0.80 ± 0.03
3	15	11.8	14.1	13.1	14.2	0.90	0.99	0.95 ± 0.05
4	20	13.0	16.8	19.3	21.0	0.68	0.80	0.74 ± 0.06
5	25	16.5	18.4	22.7	26.3	0.73	0.70	0.71 ± 0.02
6	30	23.9	25.0	27.4	26.3	0.87	0.95	0.91 ± 0.04

[a] Determined by ^{1}H-NMR analysis. [b] Second reaction set with identical conditions.

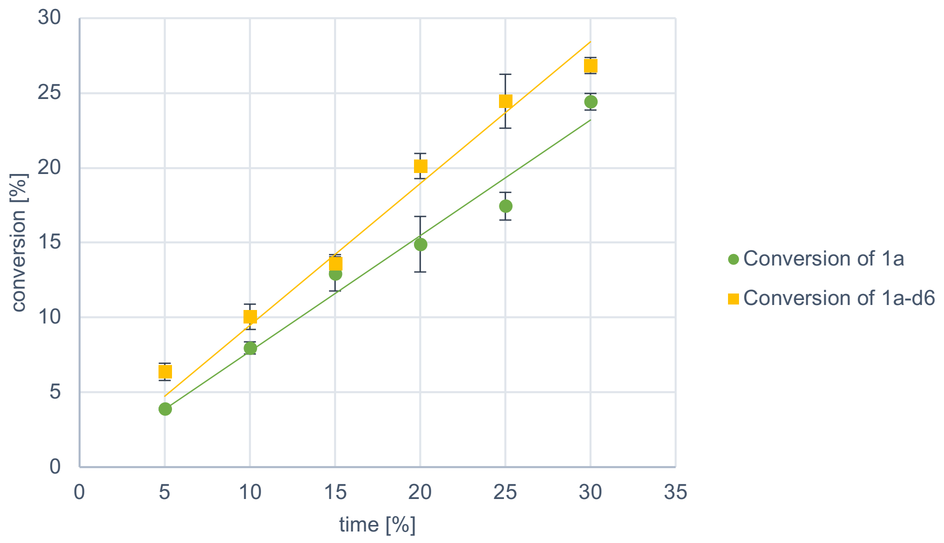

Figure S15. Average conversions of **1a** and **1a-d$_6$** using the GOx/HRP-system.

Kinetic isotope effect studies in the laccase catalyzed nitroso-ene reaction

Two reaction sets were run with **1a** and **1a-d$_6$** to calculate the kinetic isotope effect (KIE). To a 10 mM solution of substrate in 7 mL phosphate buffer (100 mM, pH 7.0) was added 1 U laccase CotA (*Bacillus licheniformis*). The reaction was incubated at 25 °C and stopped at different times (15, 30, 45, 60, 75, 90, 105, 120 min) and worked up as followed. The reaction mixture was extracted 3 x with EtOAc. The combined organic phases were dried over Na_2SO_4, filtered through a silica pad and the solvent was removed under reduced pressure. The conversion was determined by ^{1}H-NMR analysis, from which the KIE was calculated. Both reaction sets were performed twice under the same conditions. The overall average KIE was determined as a β secondary kinetic isotope effect **k_H/k_D = 0.99 ± 0.06**.

Table S4. Results of kinetic measurements of **1a** and **1a-d$_6$** using laccase CotA.

Entry	Time [min]	conversion [%][a]				k_H/k_D		
		**1a**	**1a**[b]	**1a-d$_6$**	**1a-d$_6$** [b]	1st set	2nd set	averaged
1	15	6.3	7.3	6.5	7.7	0.98	0.95	0.97 ± 0.02
2	30	8.4	8,3	7.7	8.2	1.09	1.02	1.06 ± 0.04
3	45	12.4	12.7	10.8	10.6	1.15	1.2	1.18 ± 0.03
4	60	13.2	14.3	13.6	13.3	0.97	1.08	1.03 ± 0.06
5	75	16.2	15.8	18.0	19.3	0.90	0.82	0.86 ± 0.04
6	90	17.5	19.2	20.3	21.7	0.86	0.89	0.88 ± 0.02
7	105	23.0	25.8	24.4	23.9	0.94	1.08	1.01 ± 0.07
8	120	22.9	25.3	26.4	25.4	0.87	0.99	0.93 ± 0.06

[a] Determined by ^{1}H-NMR analysis. [b] Second reaction set with identical conditions.

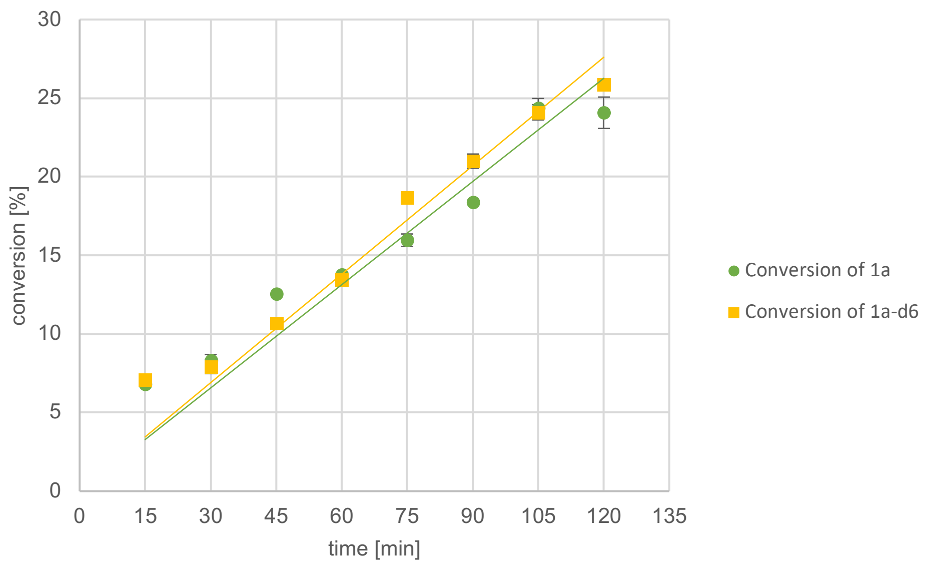

Figure S16. Average conversions of **1a** and **1a-d$_6$** using laccase CotA.

Decomposition analysis

a nitroso intermediate undergoes hydrolytic decomposition

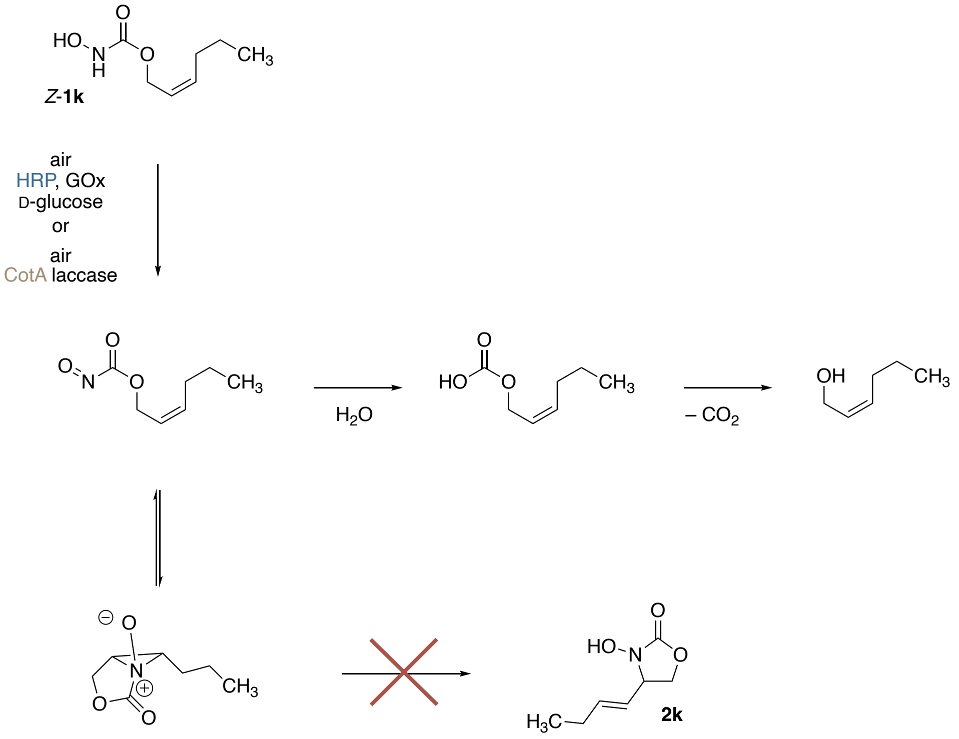

b GC chromatograms of hexenol reference and the incubation product of *Z*-**1k**

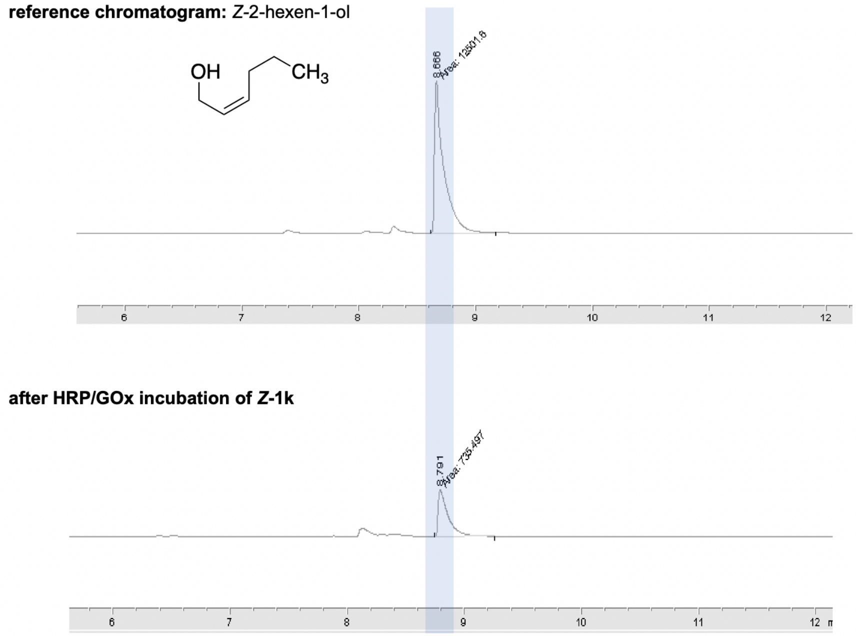

Figure S17. Proposed side reaction of non-reactive ene substrates: formation of alcohols via hydrolysis & decarboxylation:

HPLC traces

Reference chromatograms (Chiralpak IA, 3% IPA in n-Hexane, 0.8mL/min)

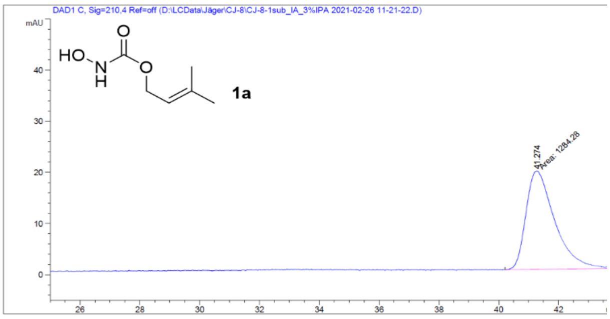

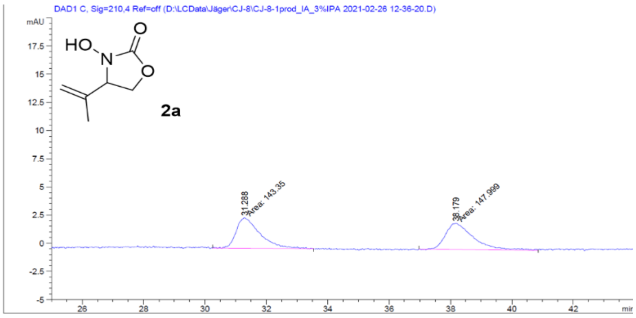

Laccase-mediated cyclizations (Chiralpak IA, 3% IPA in n-Hexane, 0.8mL/min)

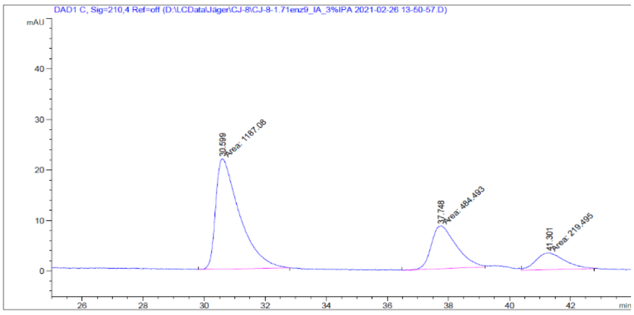

Lccβ: 46% *ee* ± 4% (n = 3)

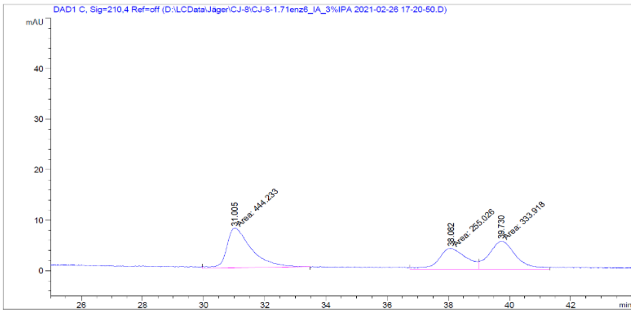

Mrl2: 27% *ee*

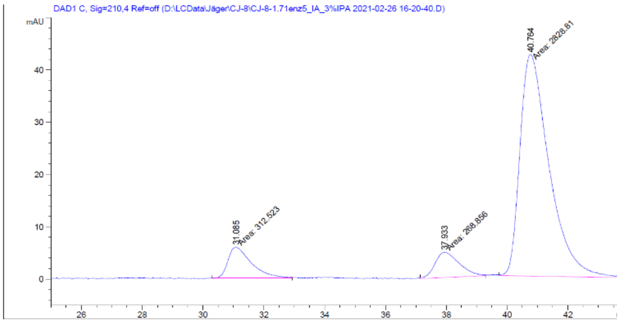

CotA: 8% *ee*

Figure S18. Reference chromatograms of **1a** and *rac*-**2a** (top), and chromatograms after incubation with laccases (bottom).

Reference chromatograms (Chiralpak IA, 3% IPA in n-Hexane, 0.8mL/min)

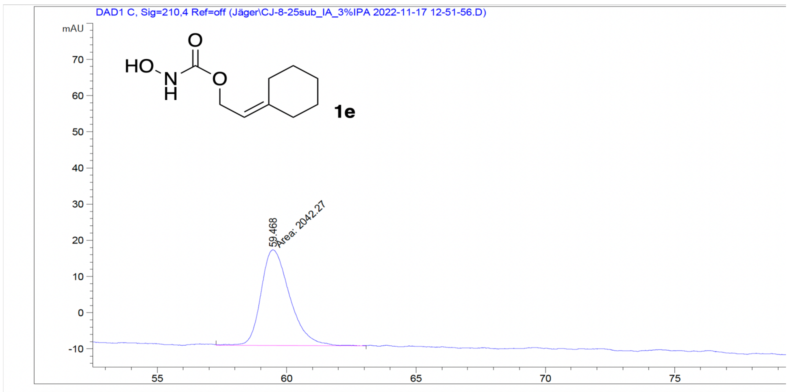

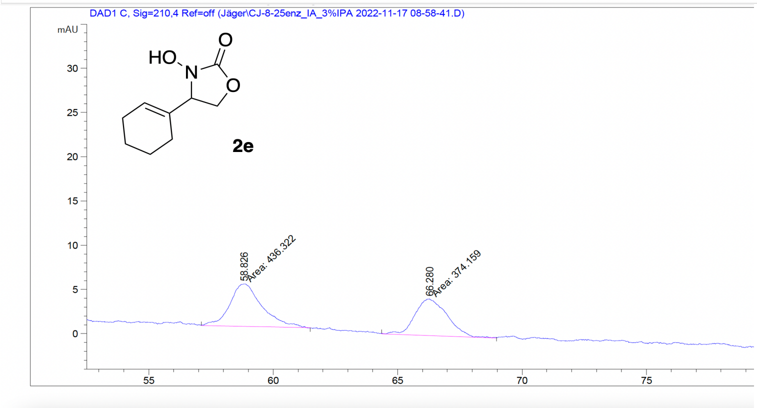

Lccβ-mediated cyclization (Chiralpak IA, 3% IPA in n-Hexane, 0.8mL/min)

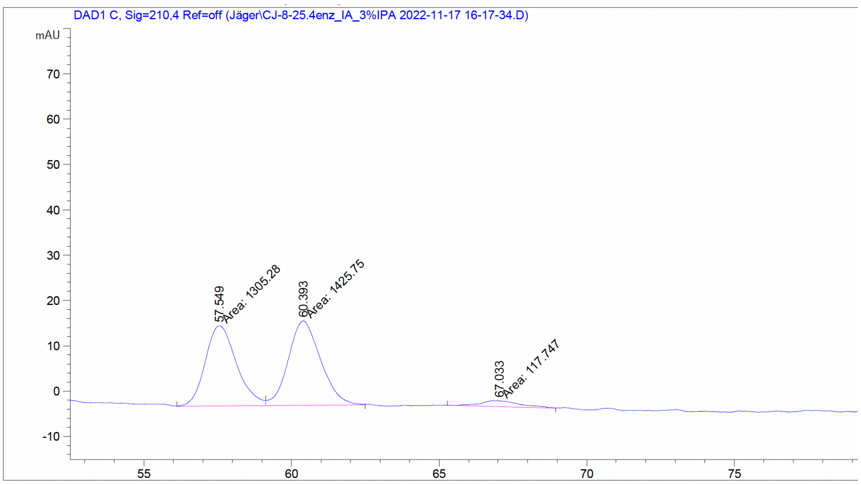

Lccβ: 82% *ee*

Figure S19. Reference chromatograms of **1e** and *rac*-**2e** (top) and chromatograms after incubation with Lccβ (bottom).

NMR Spectra

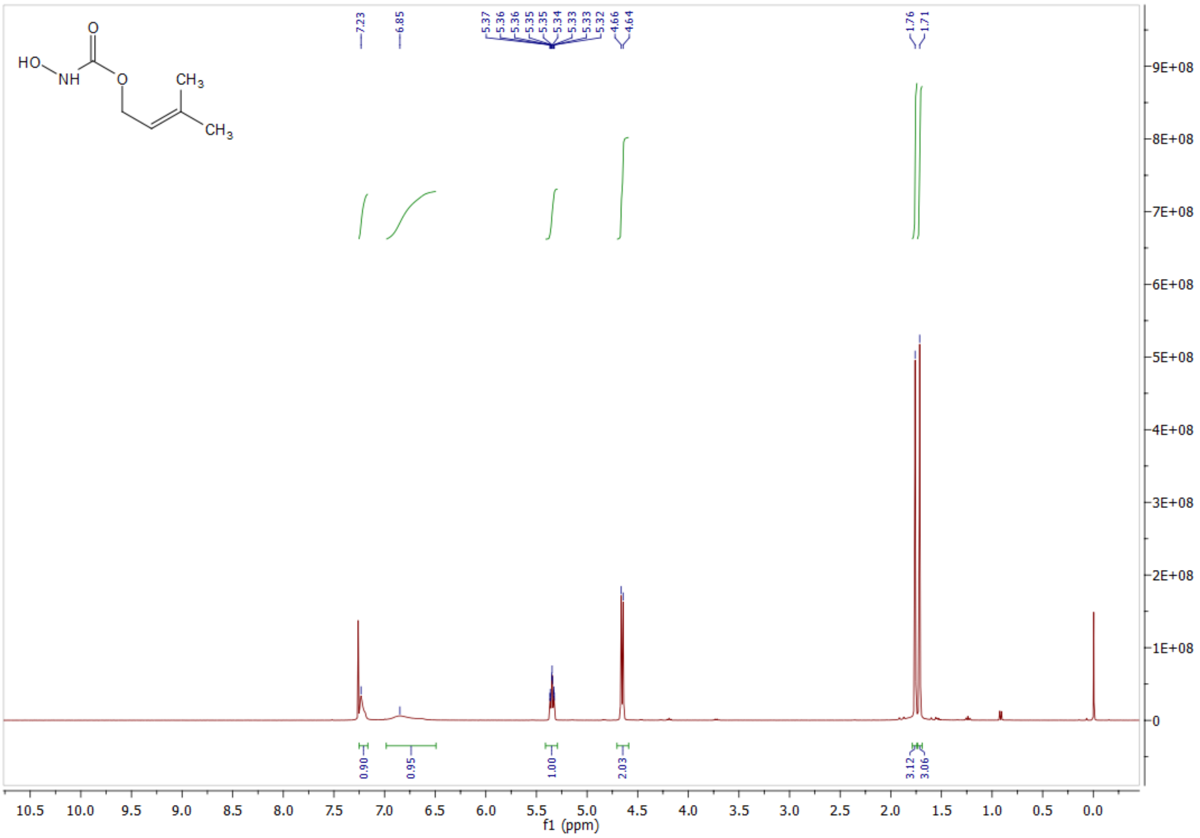
HO
NH
O
O
CH3
CH3
7.23
6.85
5.37
5.36
5.36
5.35
5.35
5.34
5.33
5.33
5.32
4.66
4.64
1.76
1.71
0.90
0.95
1.00
2.03
3.12
3.06
f1 (ppm)

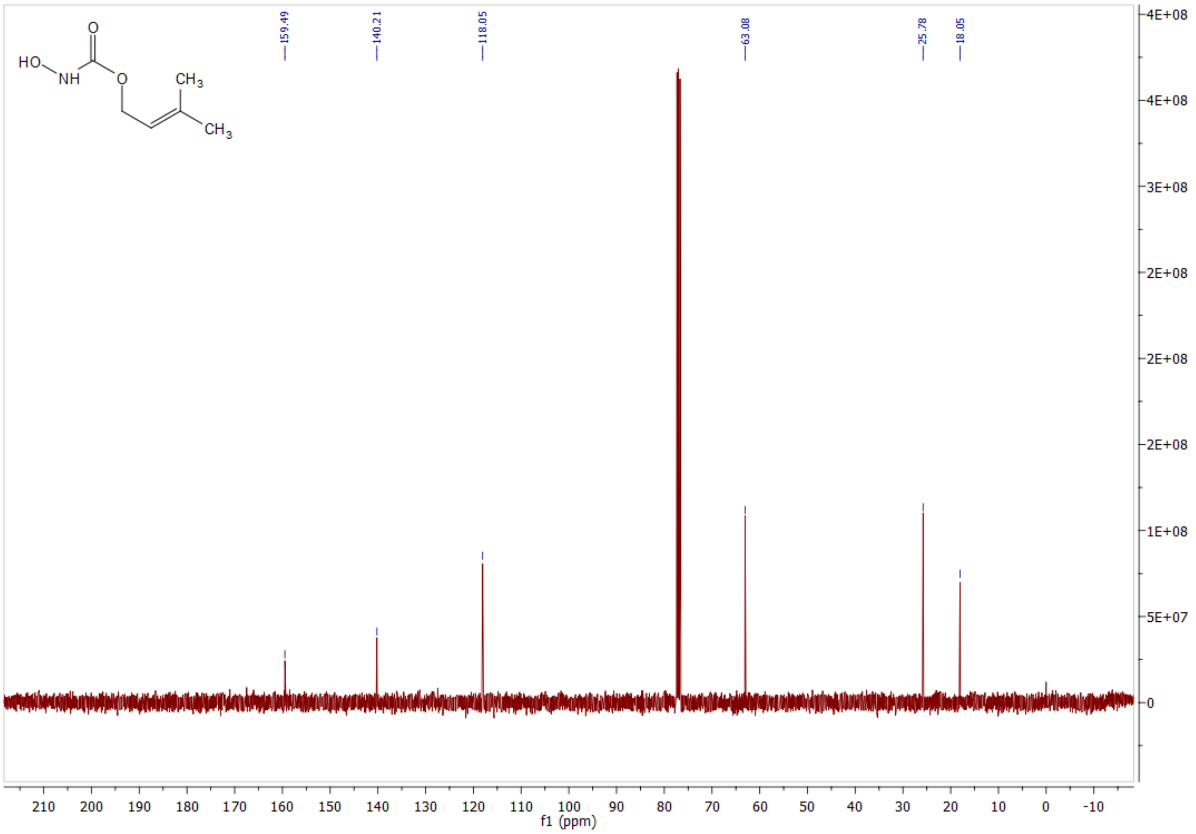
HO
NH
O
O
CH3
CH3
159.49
140.21
118.05
63.08
25.78
18.05
f1 (ppm)

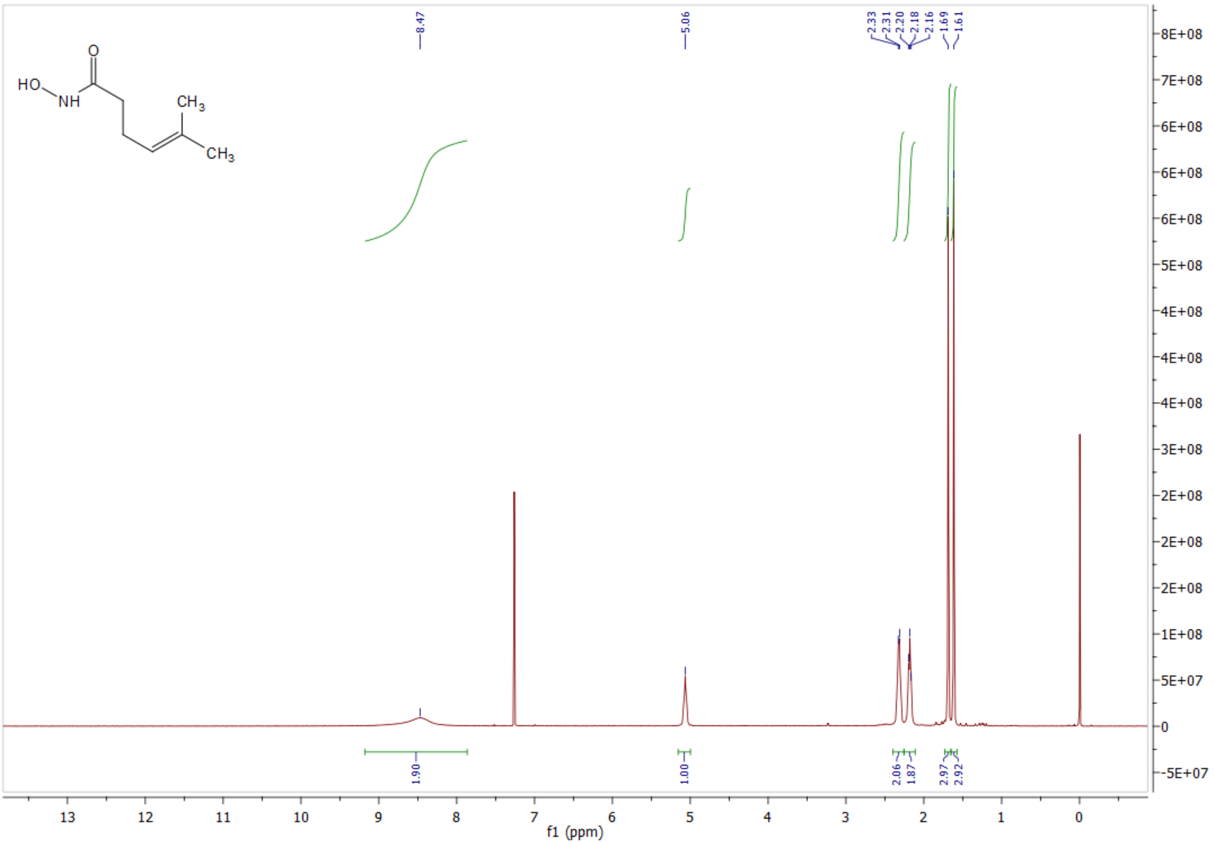
HO
NH
O
CH3
CH3
8.47
5.06
2.33
2.31
2.20
2.18
2.16
1.69
1.61
1.90
1.00
2.06
1.87
2.97
2.92
f1 (ppm)

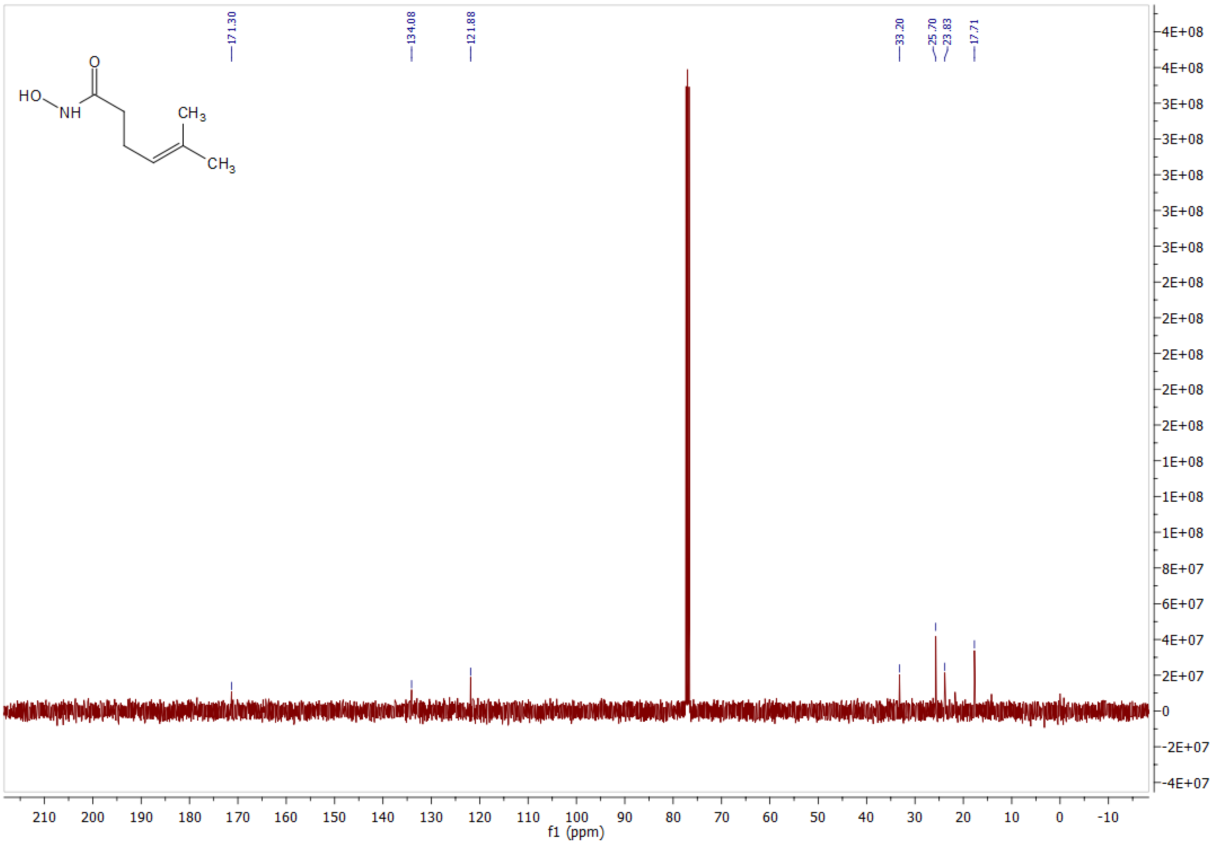
HO
NH
O
CH3
CH3
171.30
134.08
121.88
33.20
25.70
23.83
17.71
f1 (ppm)

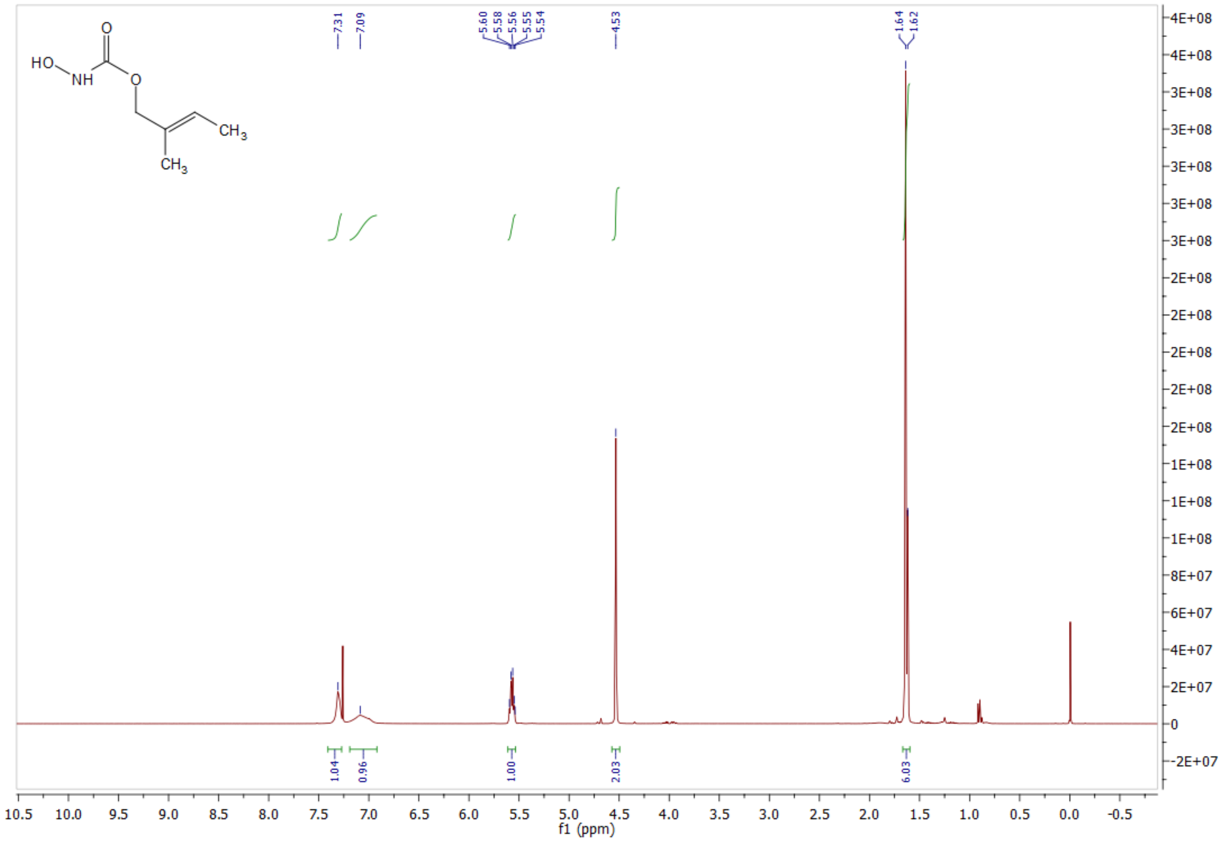
HO
NH
O
O
CH3
CH3
7.31
7.09
5.60
5.58
5.56
5.55
5.54
4.53
1.64
1.62
1.04
0.96
1.00
2.03
6.03
f1 (ppm)

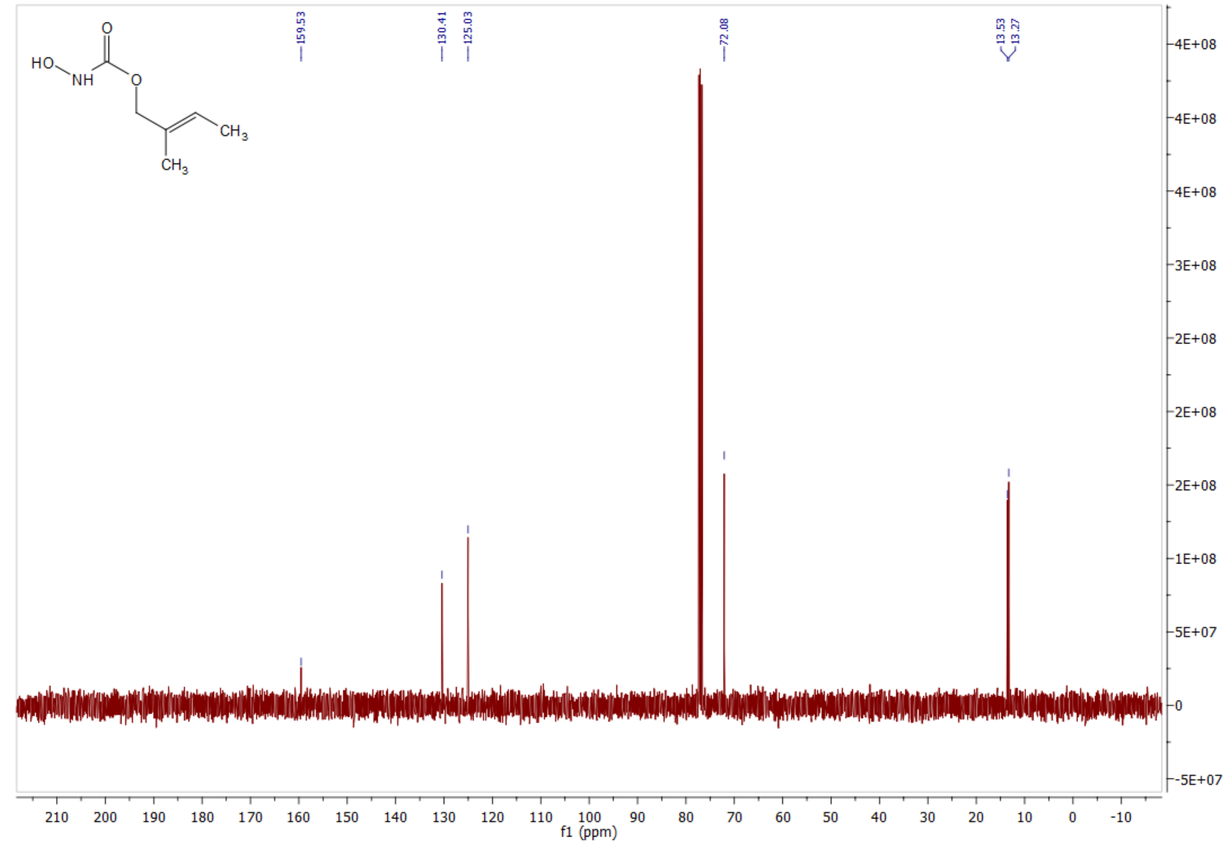
HO
NH
O
O
CH3
CH3
159.53
130.41
125.03
72.08
13.53
13.27
f1 (ppm)

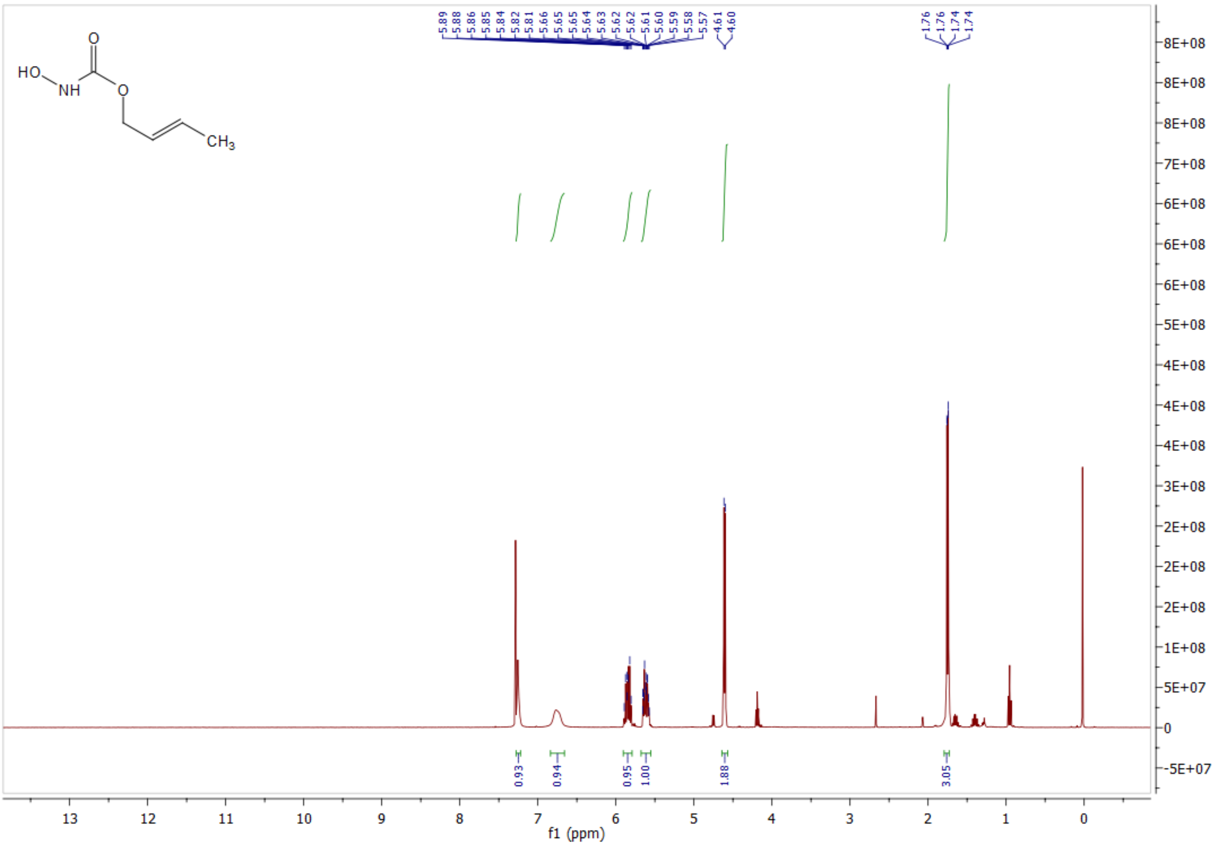
HO
NH
O
O
CH3
5.89
5.88
5.86
5.85
5.84
5.82
5.81
5.66
5.65
5.65
5.64
5.63
5.62
5.62
5.61
5.60
5.59
5.58
5.57
4.61
4.60
1.76
1.76
1.74
1.74
0.93
0.94
0.95
1.00
1.88
3.05
f1 (ppm)

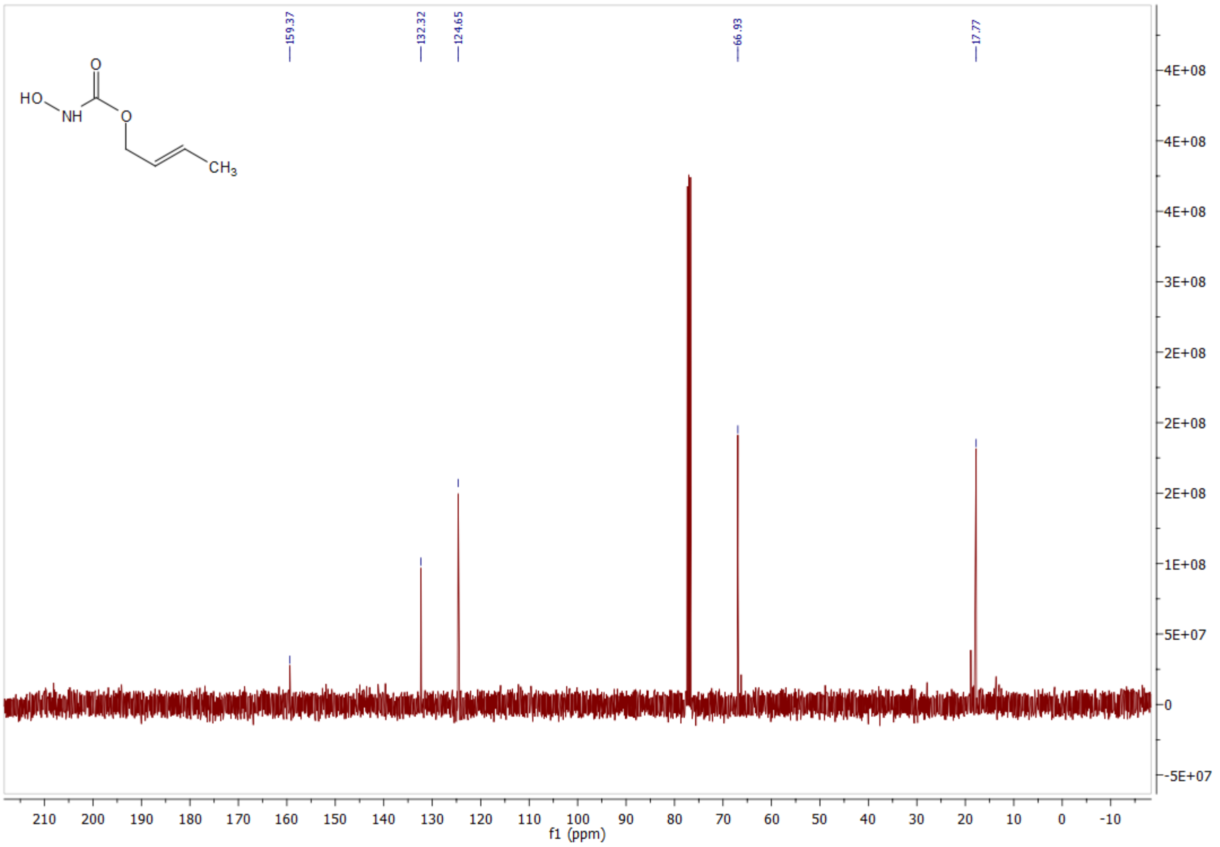
HO
NH
O
O
CH3
159.37
132.32
124.65
66.93
17.77
f1 (ppm)

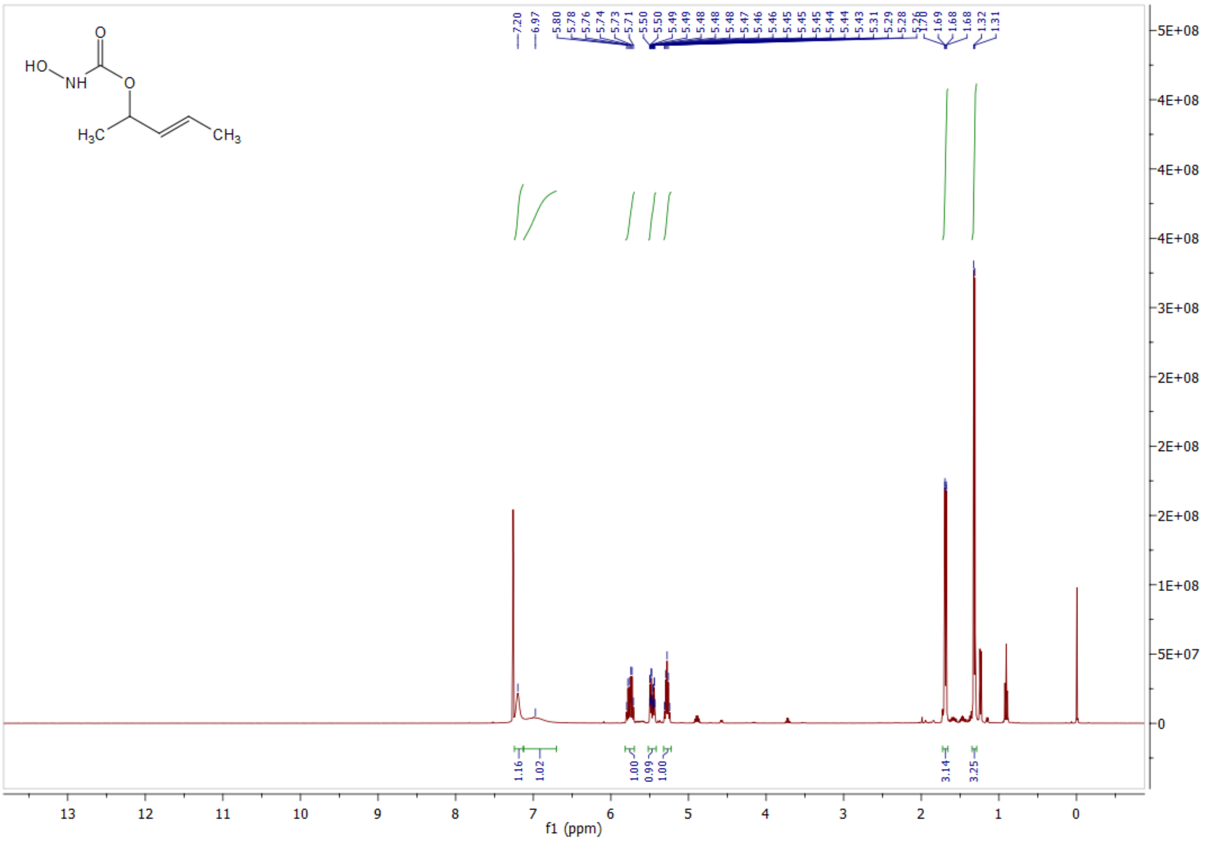
HO
NH
O
O
H3C
CH3
f1 (ppm)

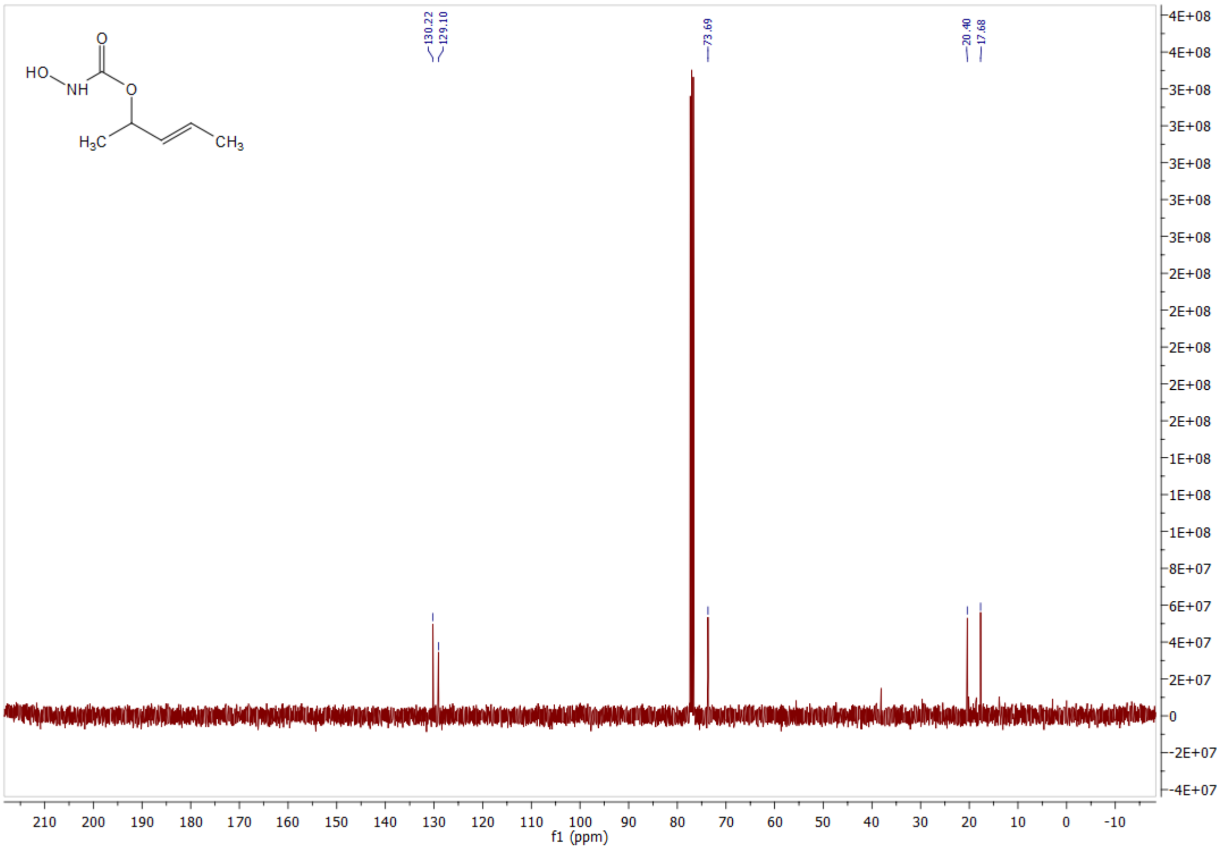
HO
NH
O
O
H3C
CH3
130.22
129.10
73.69
20.40
17.68
f1 (ppm)

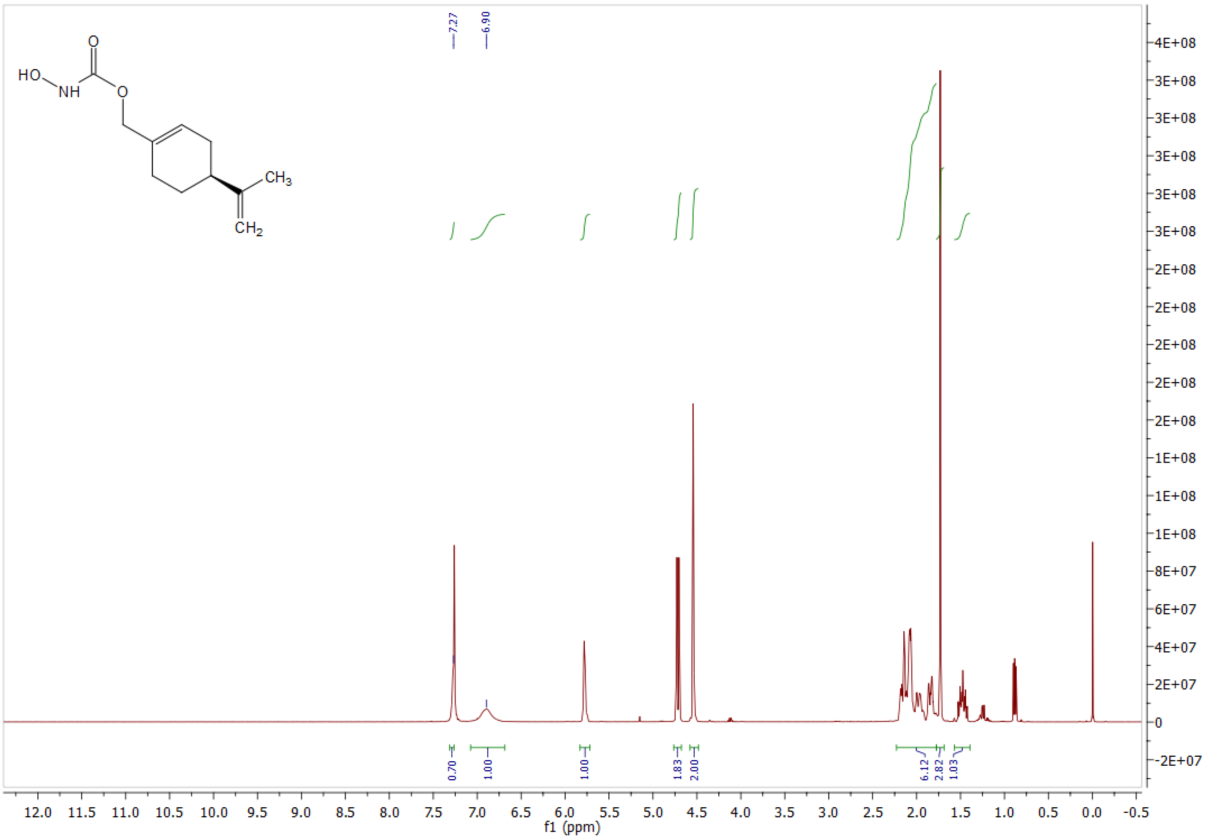
HO
NH
O
O
CH3
CH2
7.27
6.90
0.70
1.00
1.00
1.83
2.00
6.12
2.82
1.03
f1 (ppm)

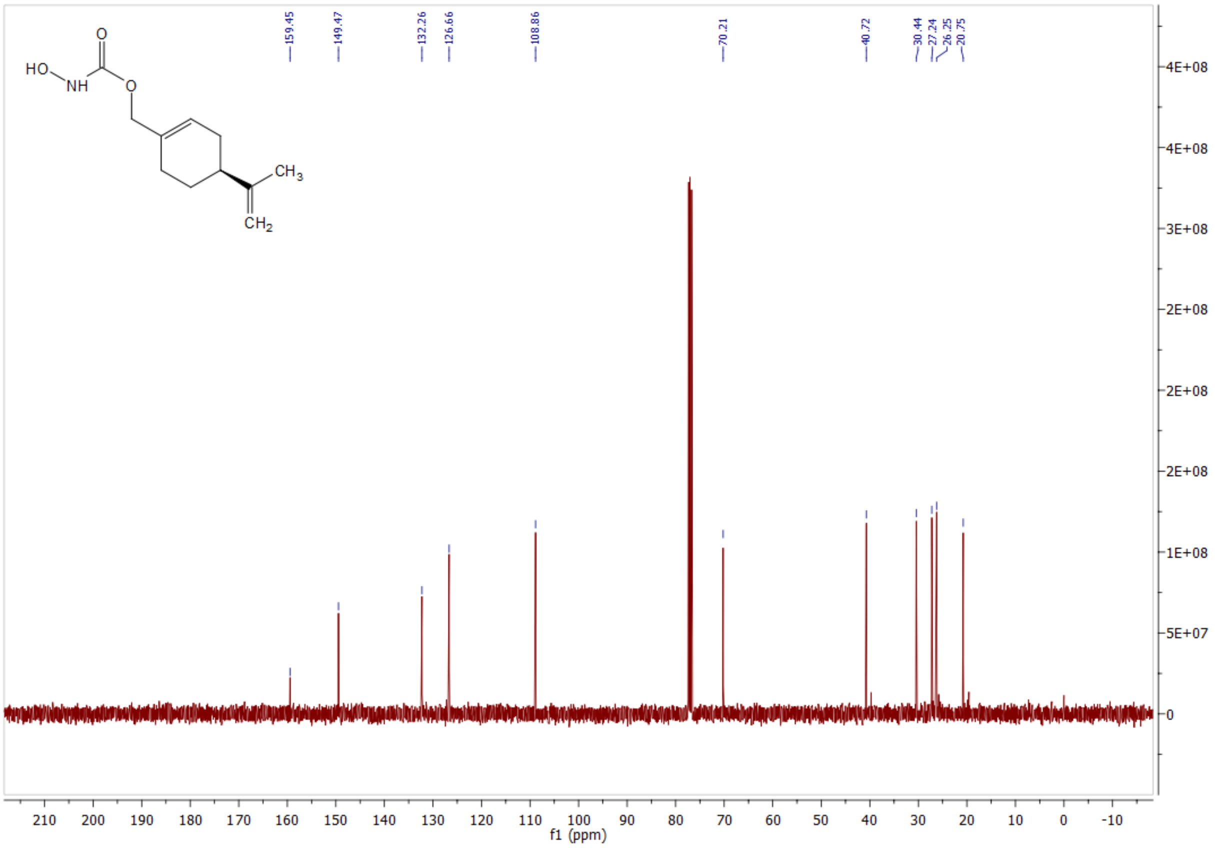
HO
NH
O
O
CH3
CH2
159.45
149.47
132.26
126.66
108.86
70.21
40.72
30.44
27.24
26.25
20.75
f1 (ppm)

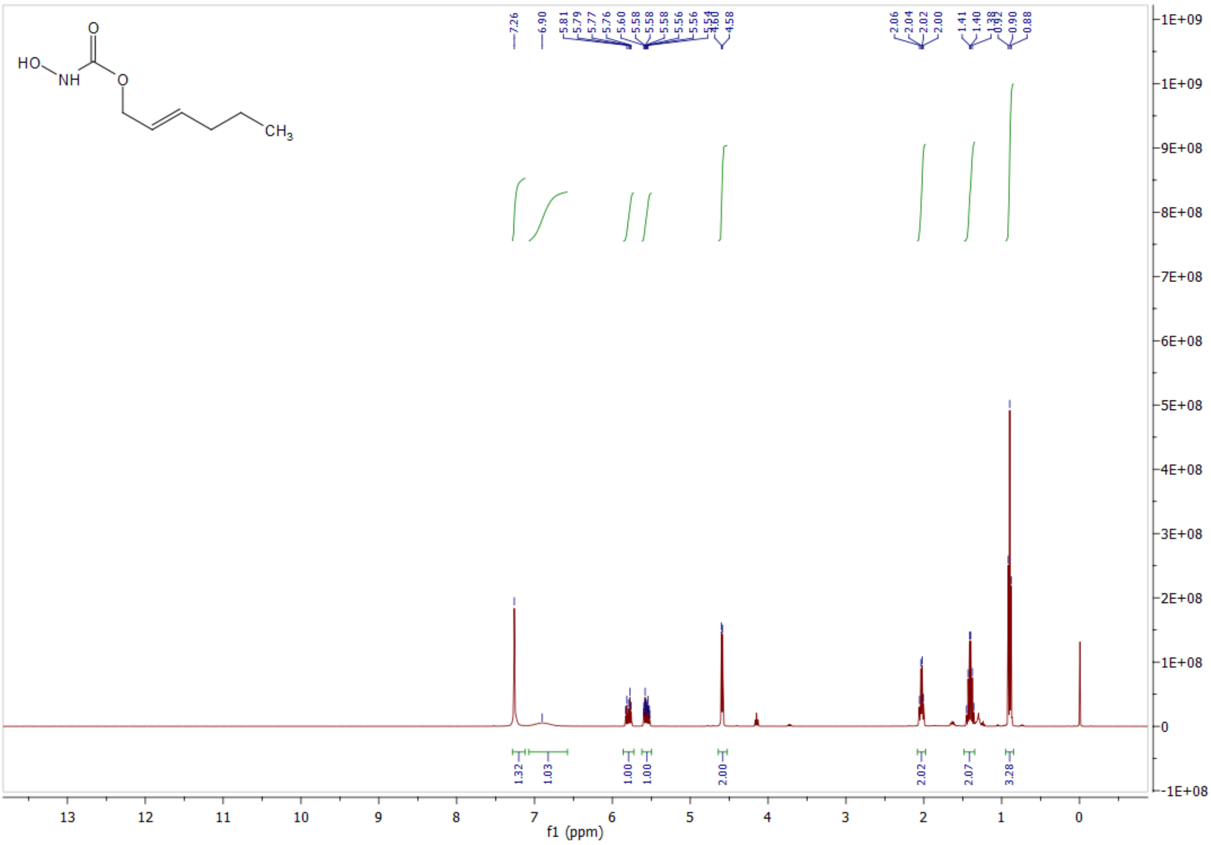
HO
NH
O
O
CH3
7.26
6.90
5.81
5.79
5.77
5.76
5.60
5.58
5.58
5.58
5.56
5.56
4.58
2.06
2.04
2.02
2.00
1.41
1.40
0.90
0.88
1.32
1.03
1.00
1.00
2.00
2.02
2.07
3.28
f1 (ppm)

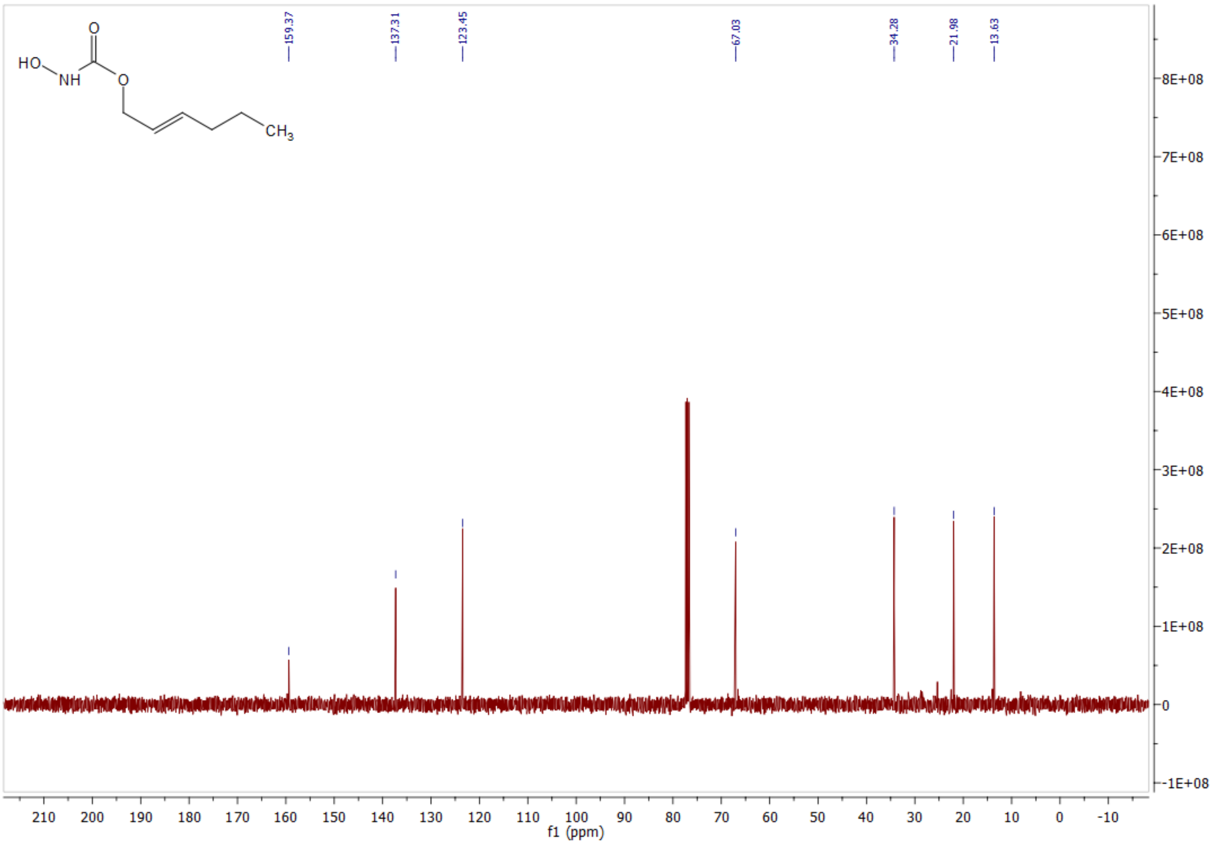
HO
NH
O
O
CH3
159.37
137.31
123.45
67.03
34.28
21.98
13.63
f1 (ppm)

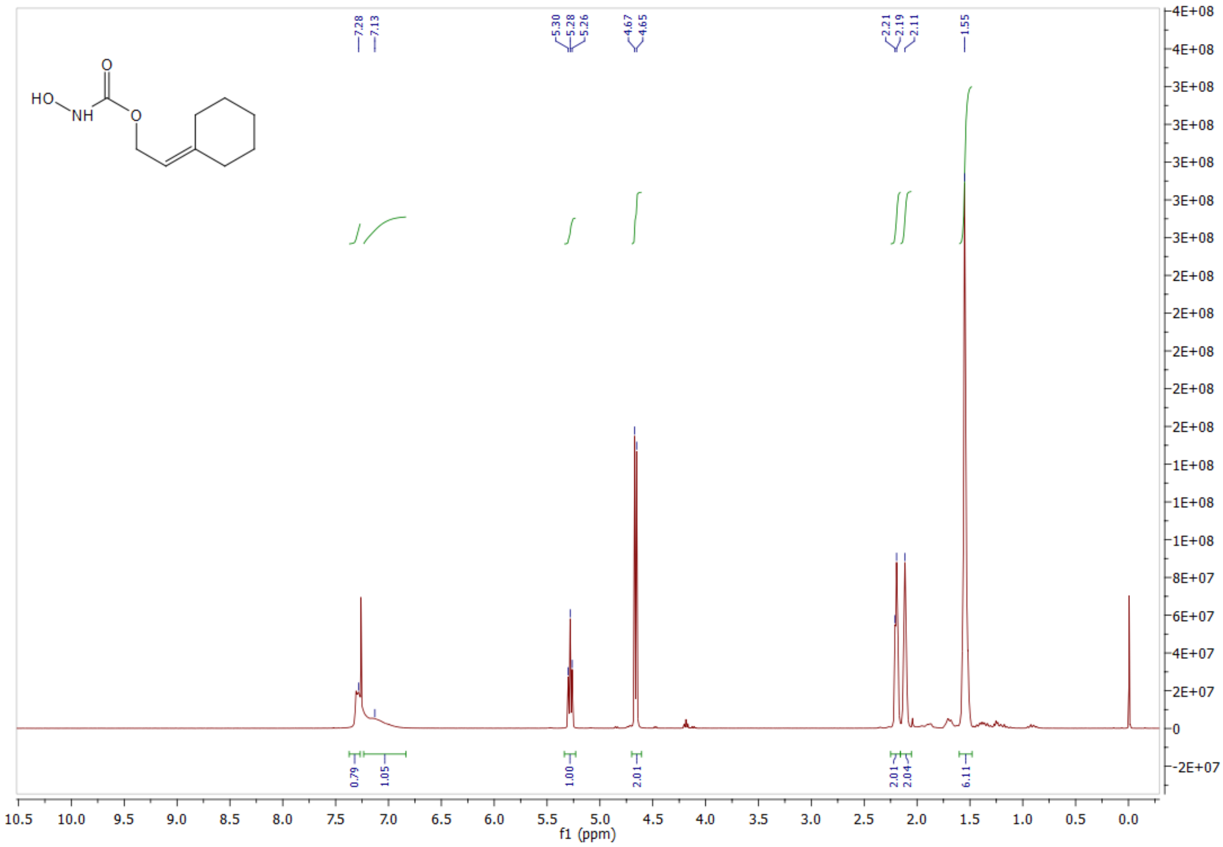
HO
NH
O
O
7.28
7.13
5.30
5.28
5.26
4.67
4.65
2.21
2.19
2.11
1.55
0.79
1.05
1.00
2.01
2.01
2.04
6.11
f1 (ppm)

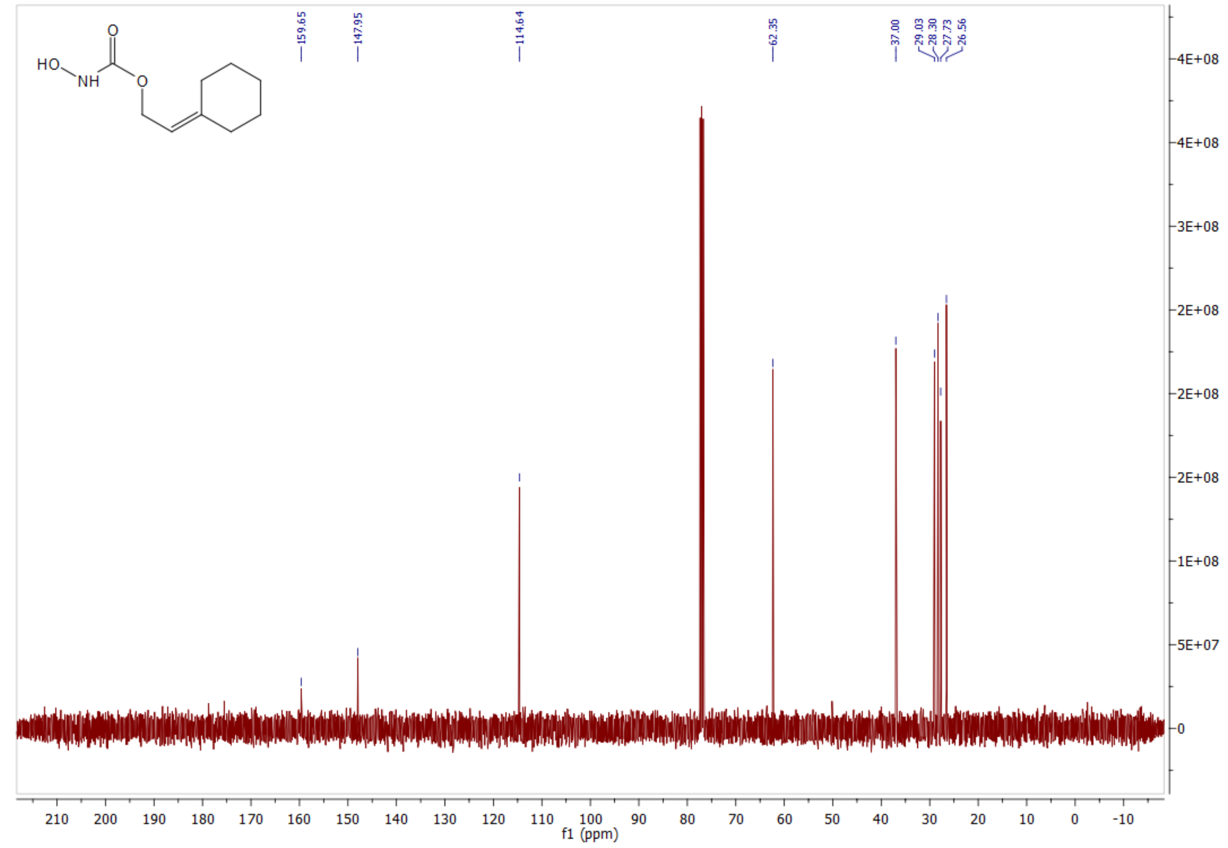
HO
NH
O
O
159.65
147.95
114.64
62.35
37.00
29.03
28.30
27.73
26.56
f1 (ppm)

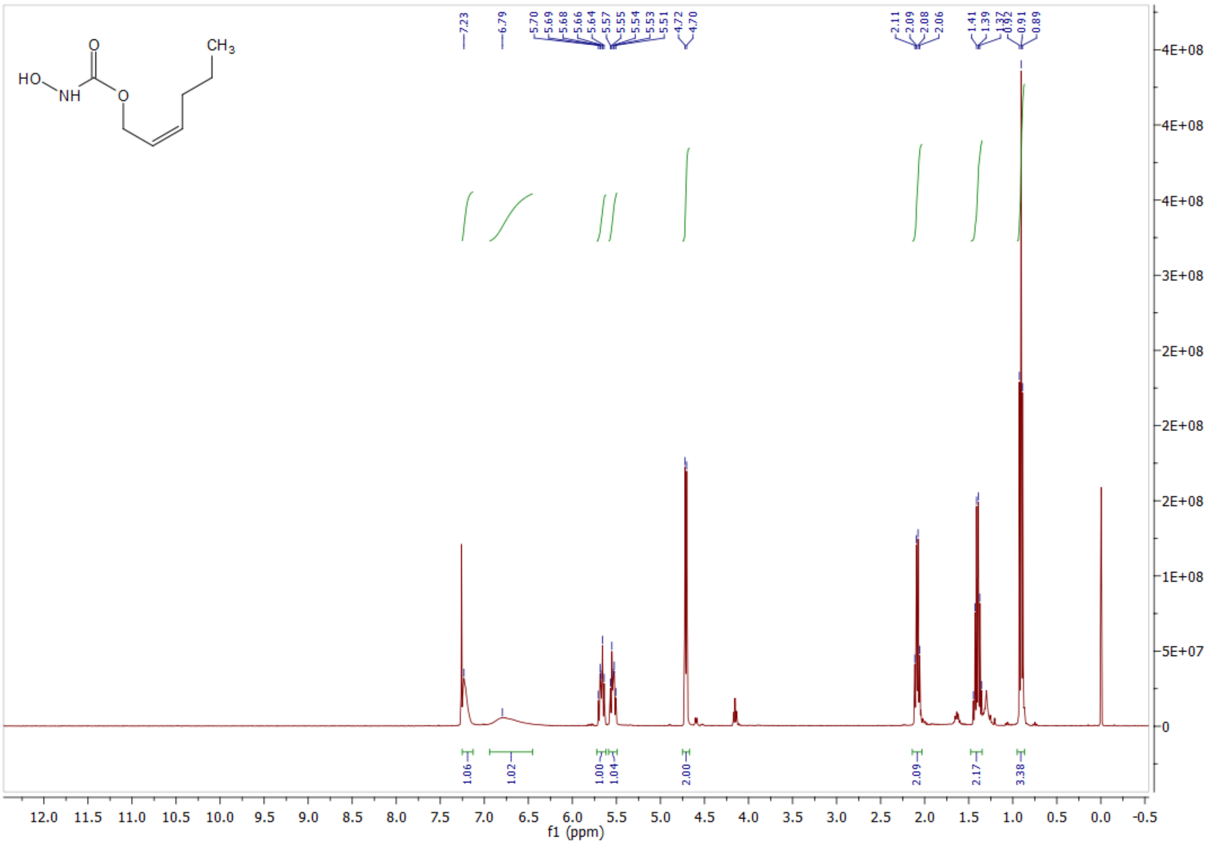
HO
NH
O
O
CH3
7.23
6.79
5.70
5.69
5.68
5.66
5.64
5.57
5.55
5.55
5.54
5.53
5.51
4.72
4.70
2.11
2.09
2.08
2.06
1.41
1.39
1.37
0.92
0.91
0.89
1.06
1.02
1.00
1.04
2.00
2.09
2.17
3.38
f1 (ppm)

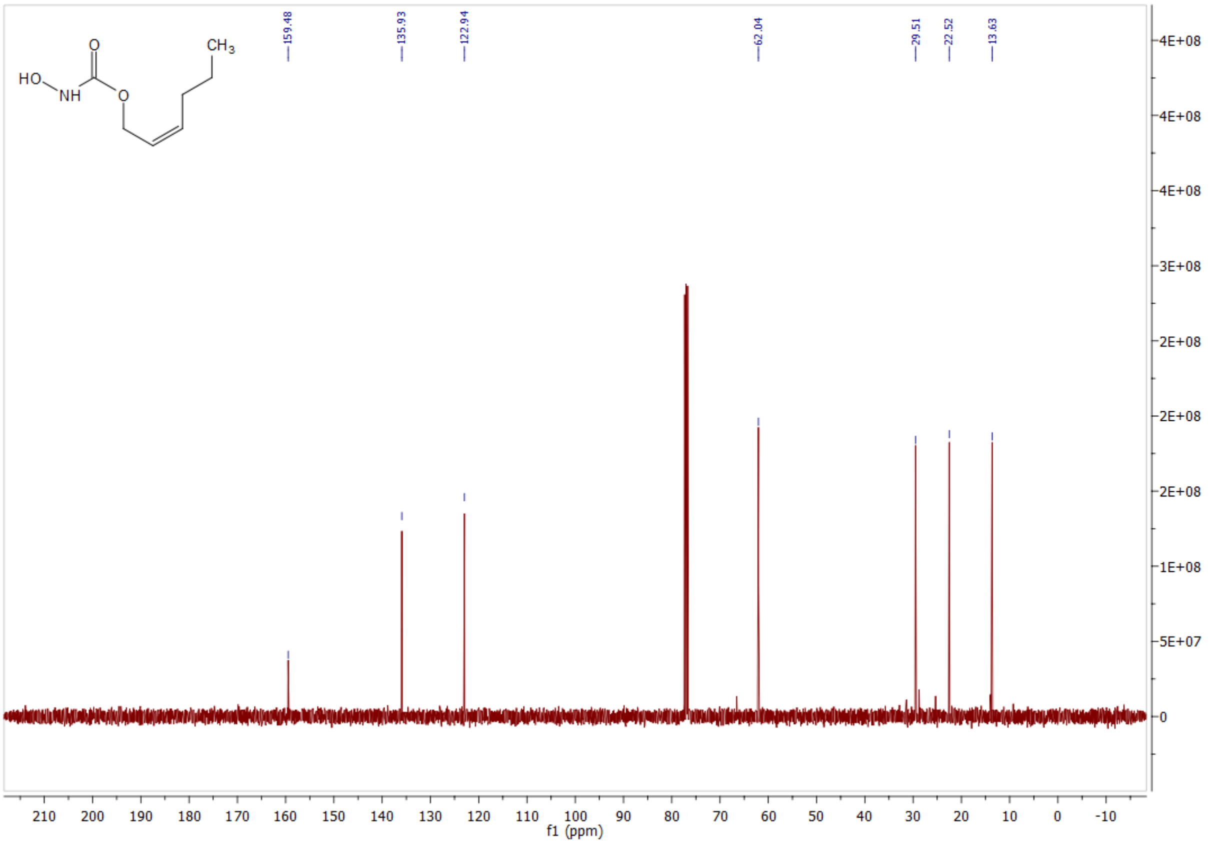
HO
NH
O
O
CH3
159.48
135.93
122.94
62.04
29.51
22.52
13.63
f1 (ppm)

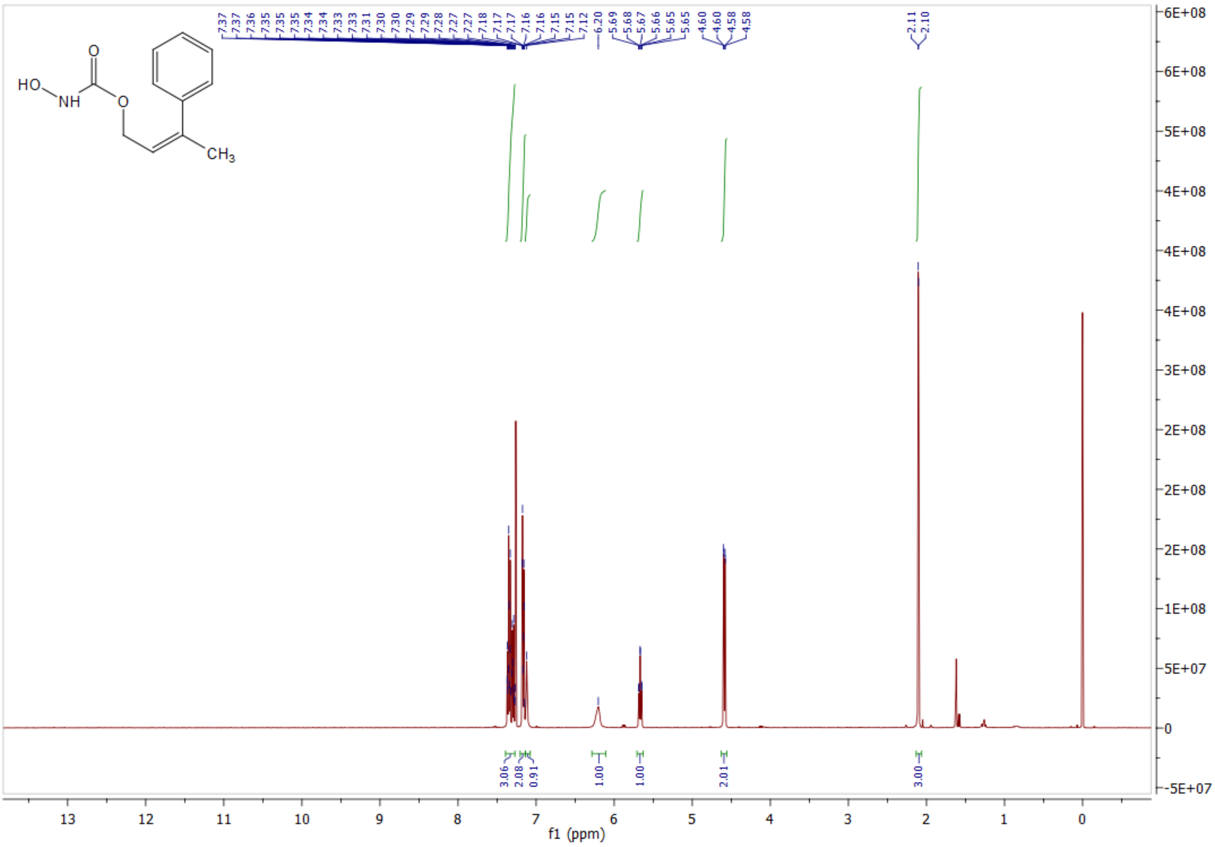

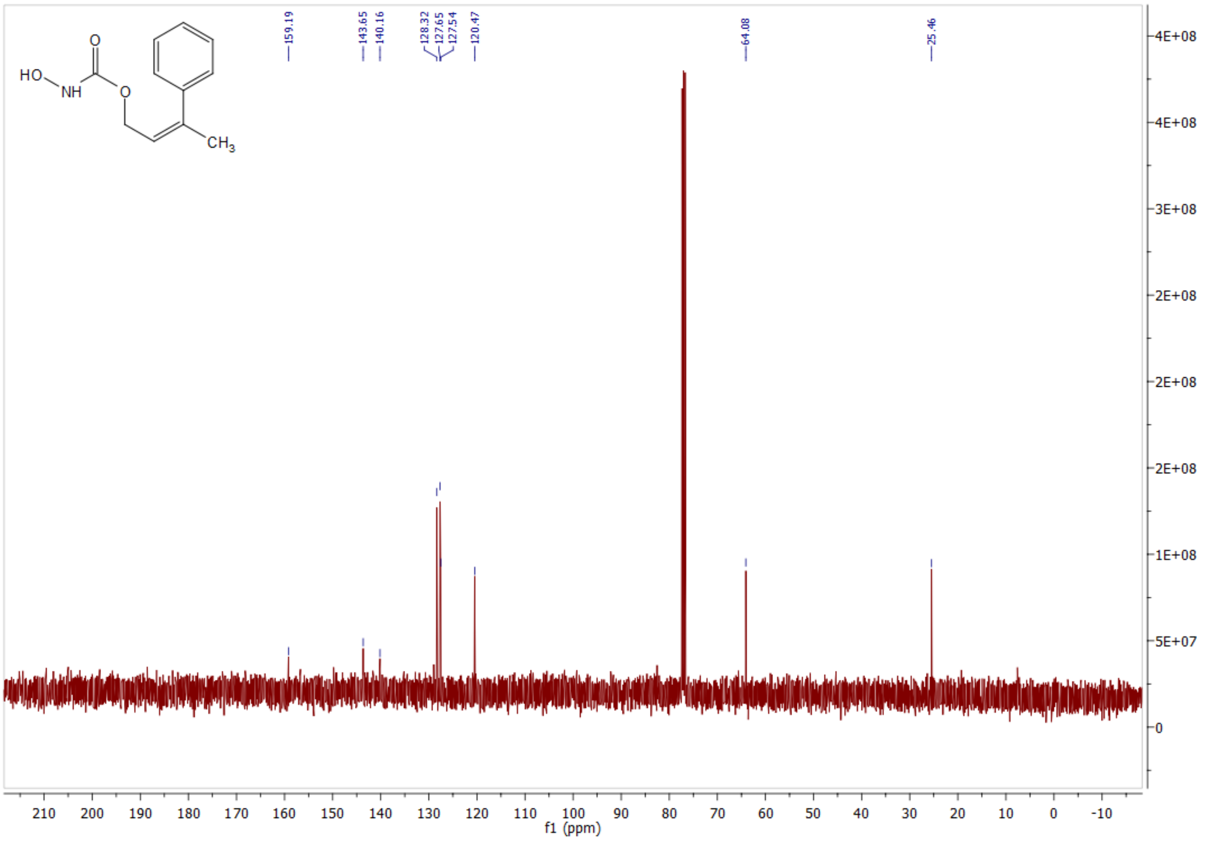

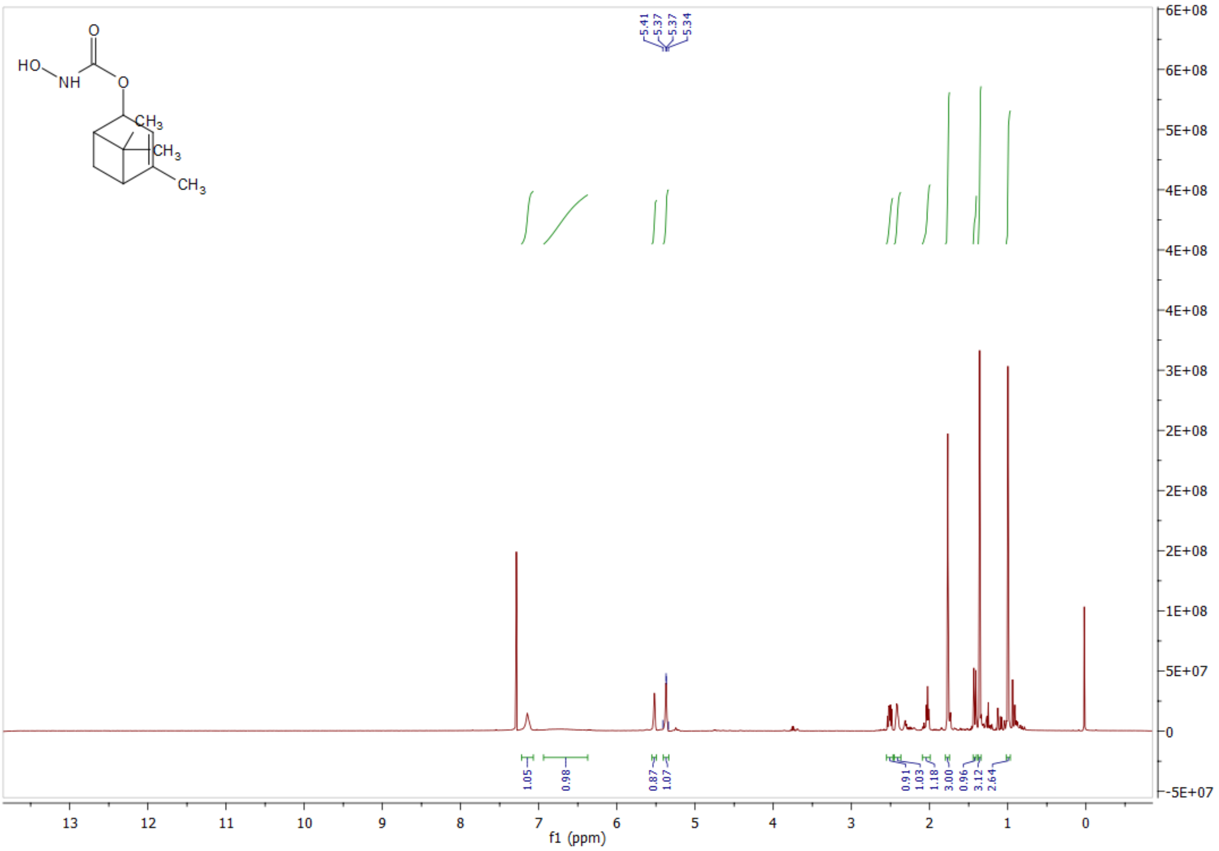
HO
NH
O
O
CH3
CH3
CH3
5.41
5.37
5.37
5.34
1.05
0.98
0.87
1.07
0.91
1.03
1.18
3.00
0.96
3.12
2.64
f1 (ppm)

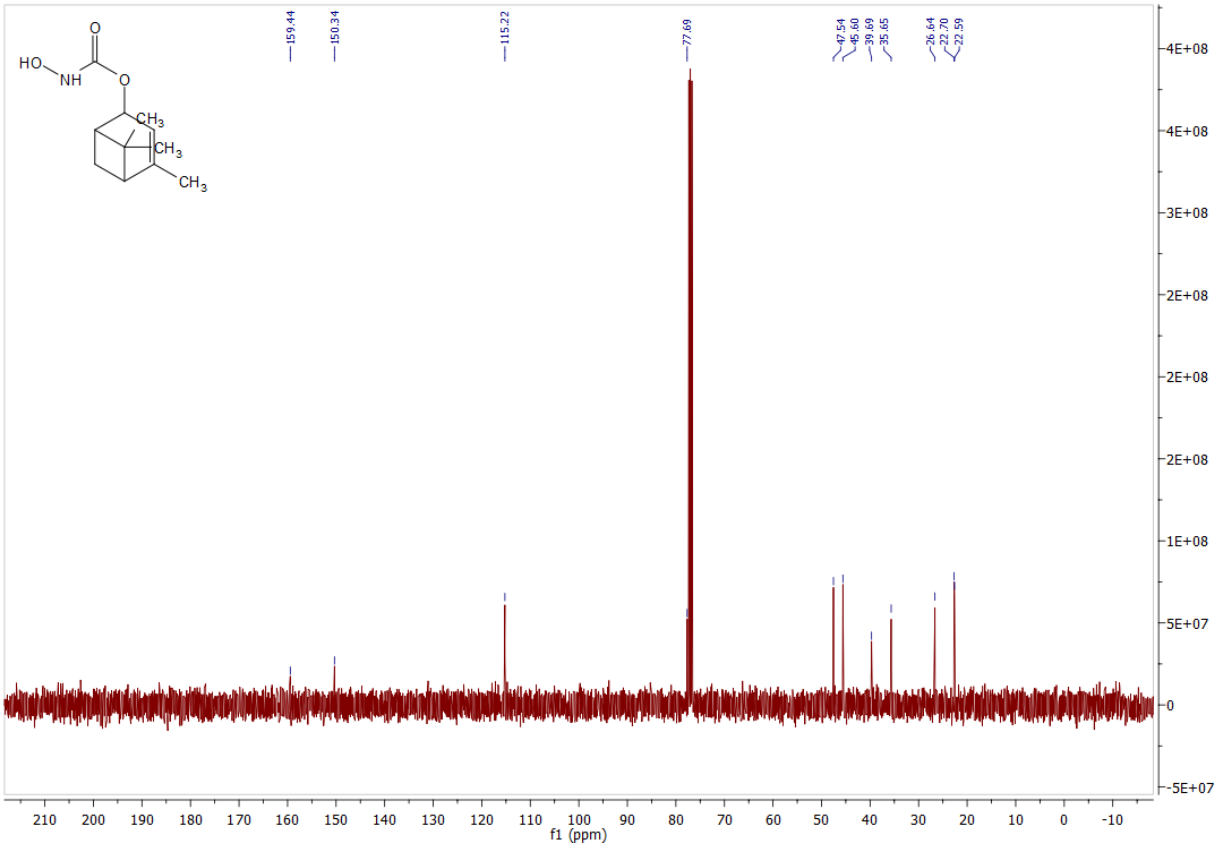
HO
NH
O
O
CH3
CH3
CH3
159.44
150.34
115.22
77.69
47.54
45.60
39.69
35.65
26.64
22.70
22.59
f1 (ppm)

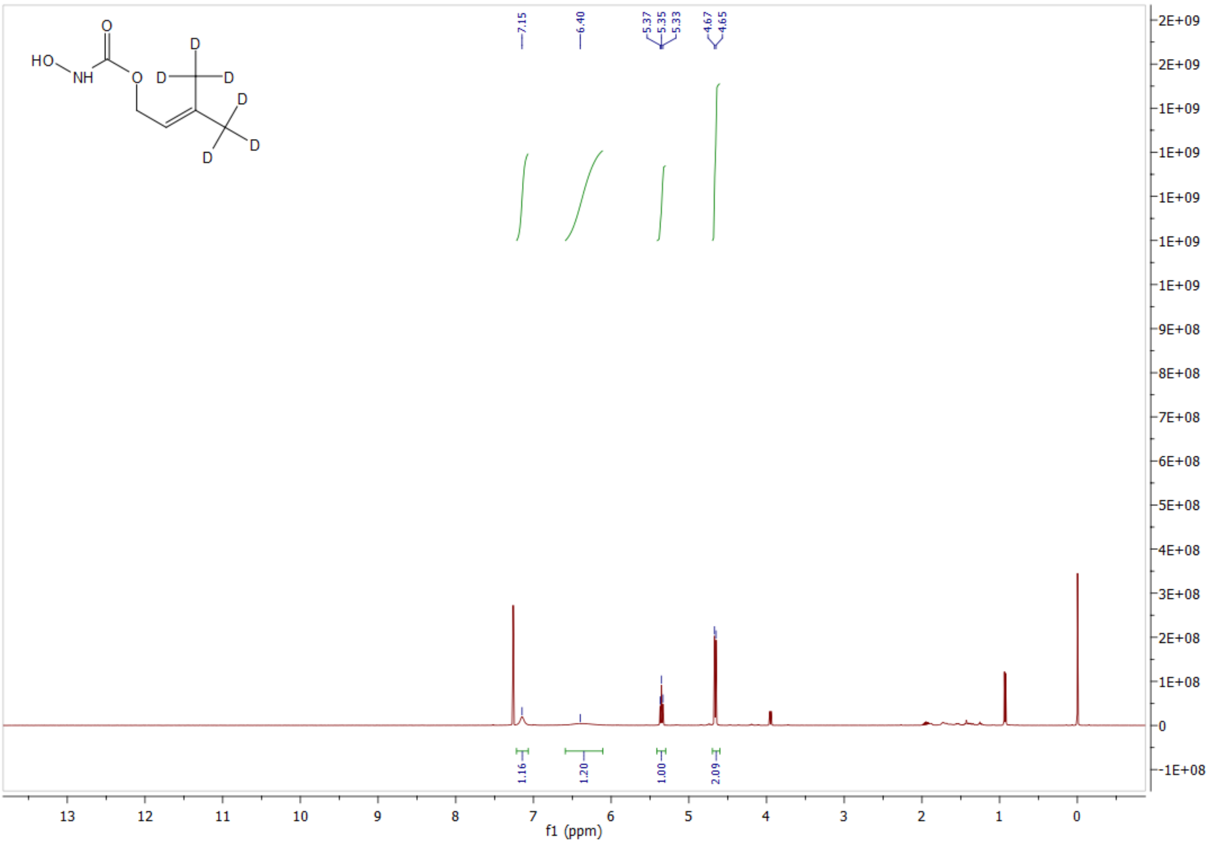
HO
NH
O
O
D
D
D
D
D
D
7.15
6.40
5.37
5.35
5.33
4.67
4.65
1.16
1.20
1.00
2.09
f1 (ppm)

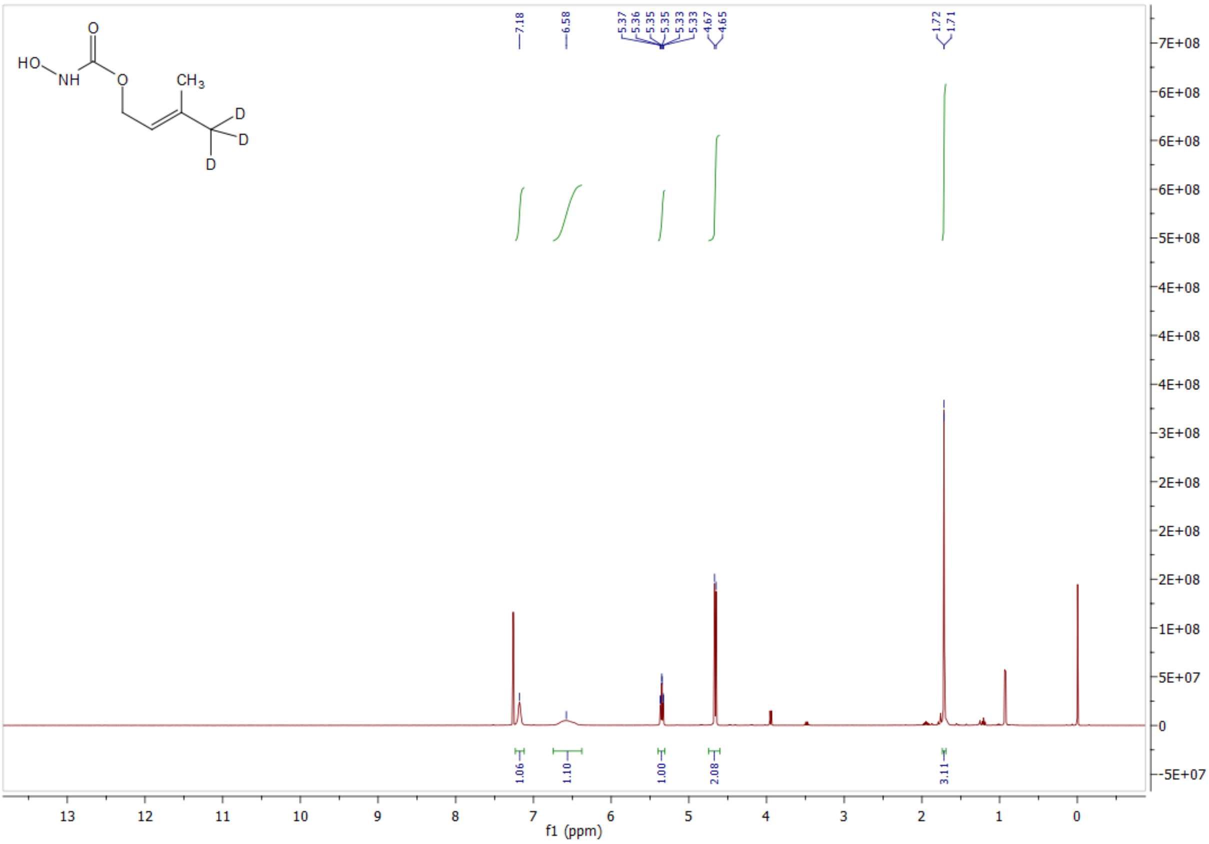
HO
NH
O
O
CH3
D
D
D
7.18
6.58
5.37
5.36
5.35
5.35
5.33
5.33
4.67
4.65
1.72
1.71
1.06
1.10
1.00
2.08
3.11
f1 (ppm)

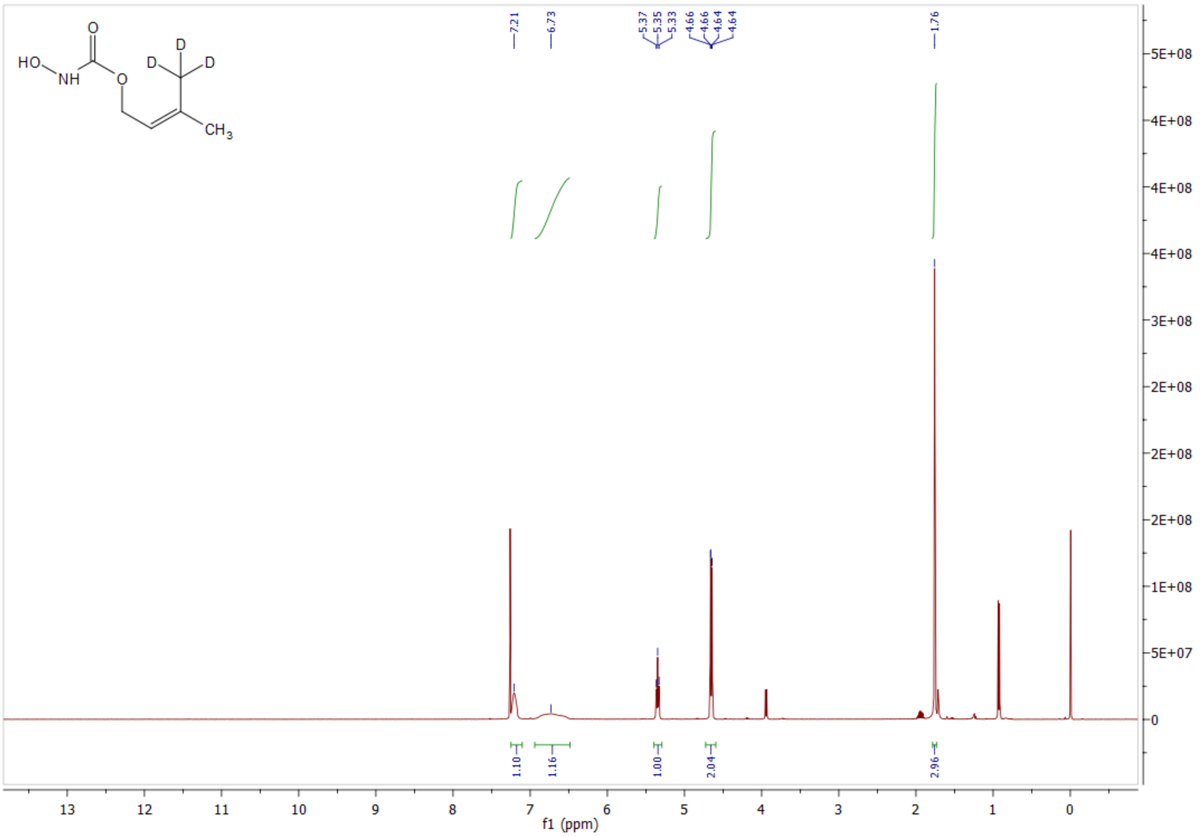
HO
NH
O
O
D
D
D
CH3
7.21
6.73
5.37
5.35
5.33
4.66
4.66
4.64
4.64
1.76
5E+08
4E+08
4E+08
4E+08
3E+08
2E+08
2E+08
2E+08
1E+08
5E+07
0
1.10
1.16
1.00
2.04
2.96
13
12
11
10
9
8
7
6
5
4
3
2
1
0
f1 (ppm)

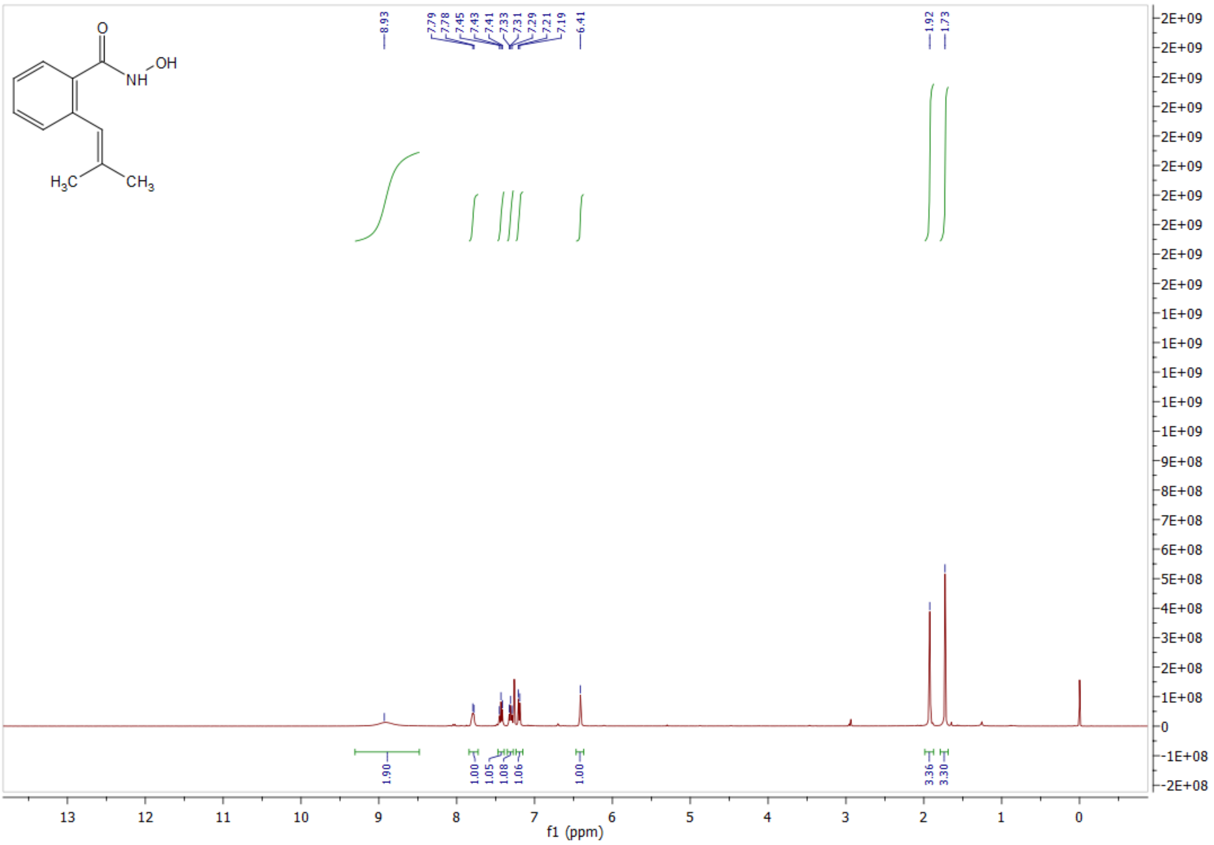

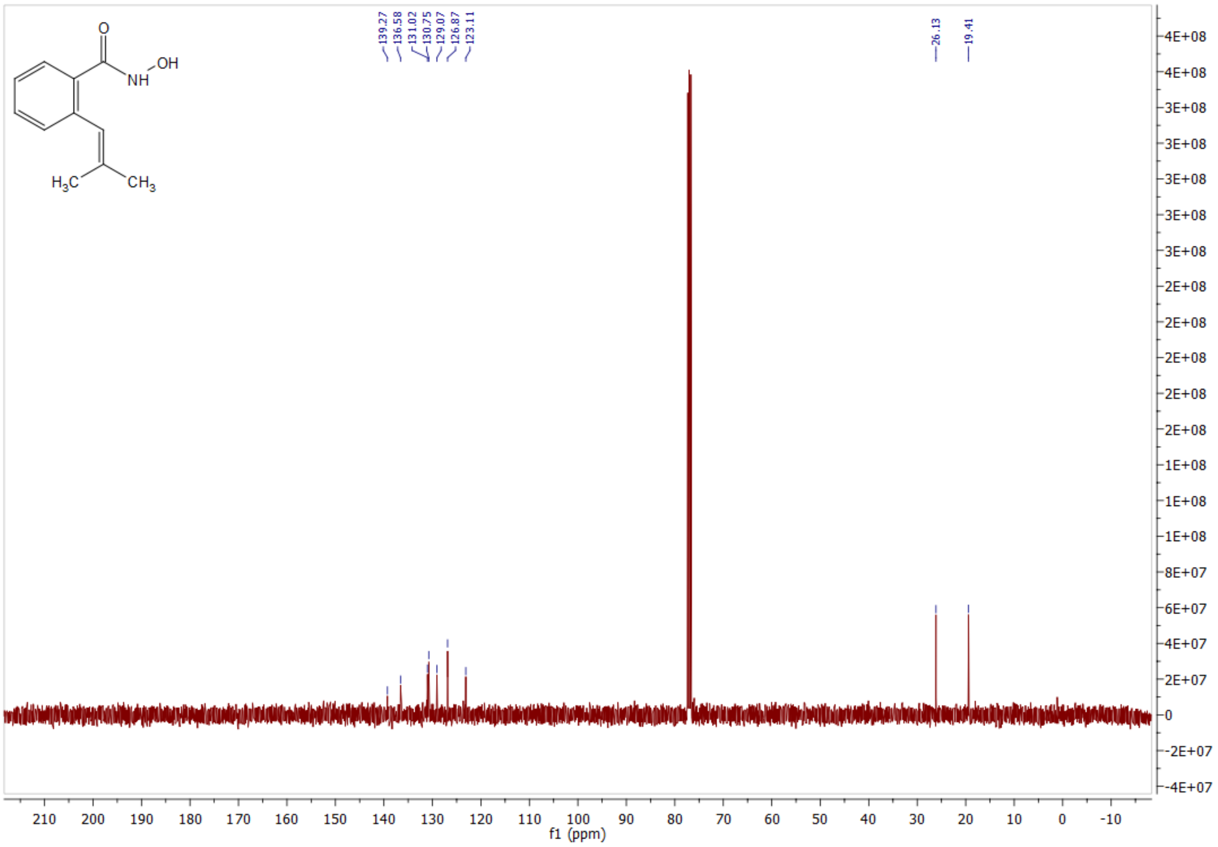

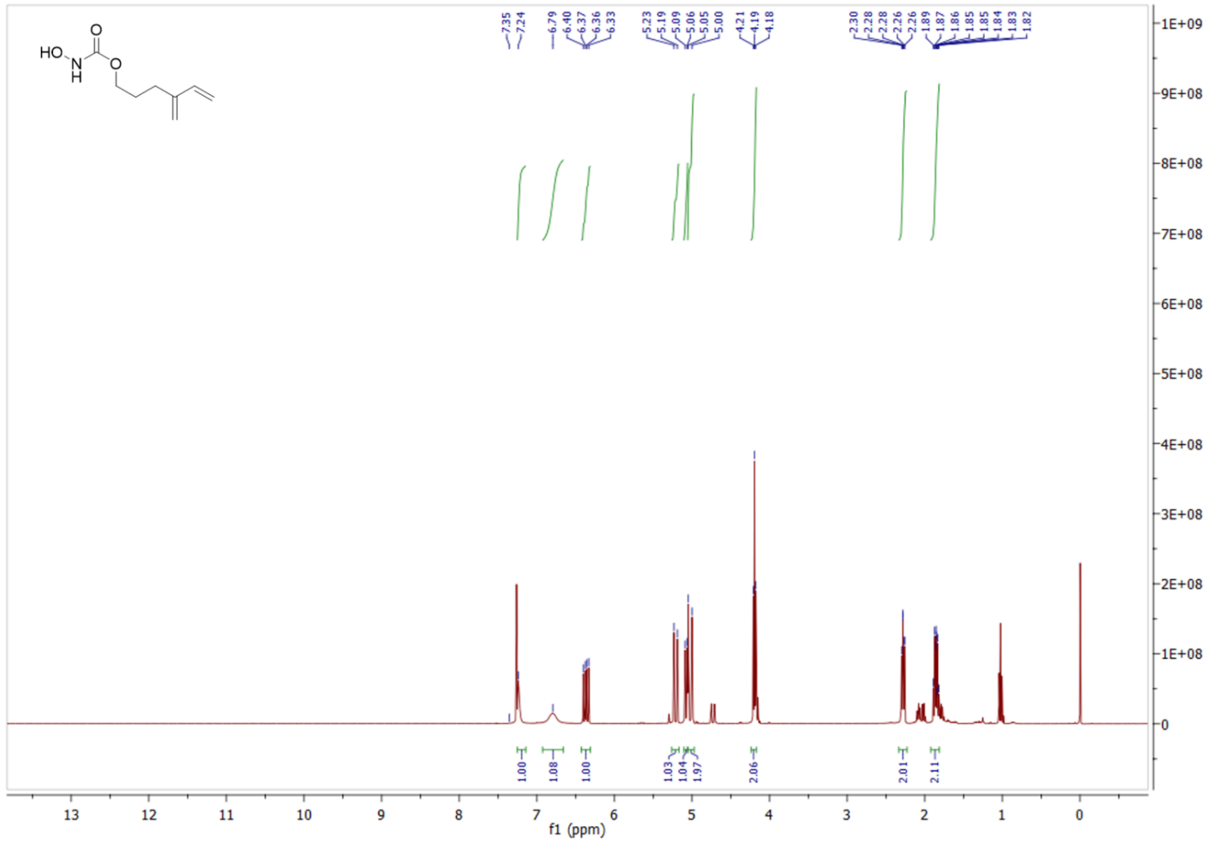

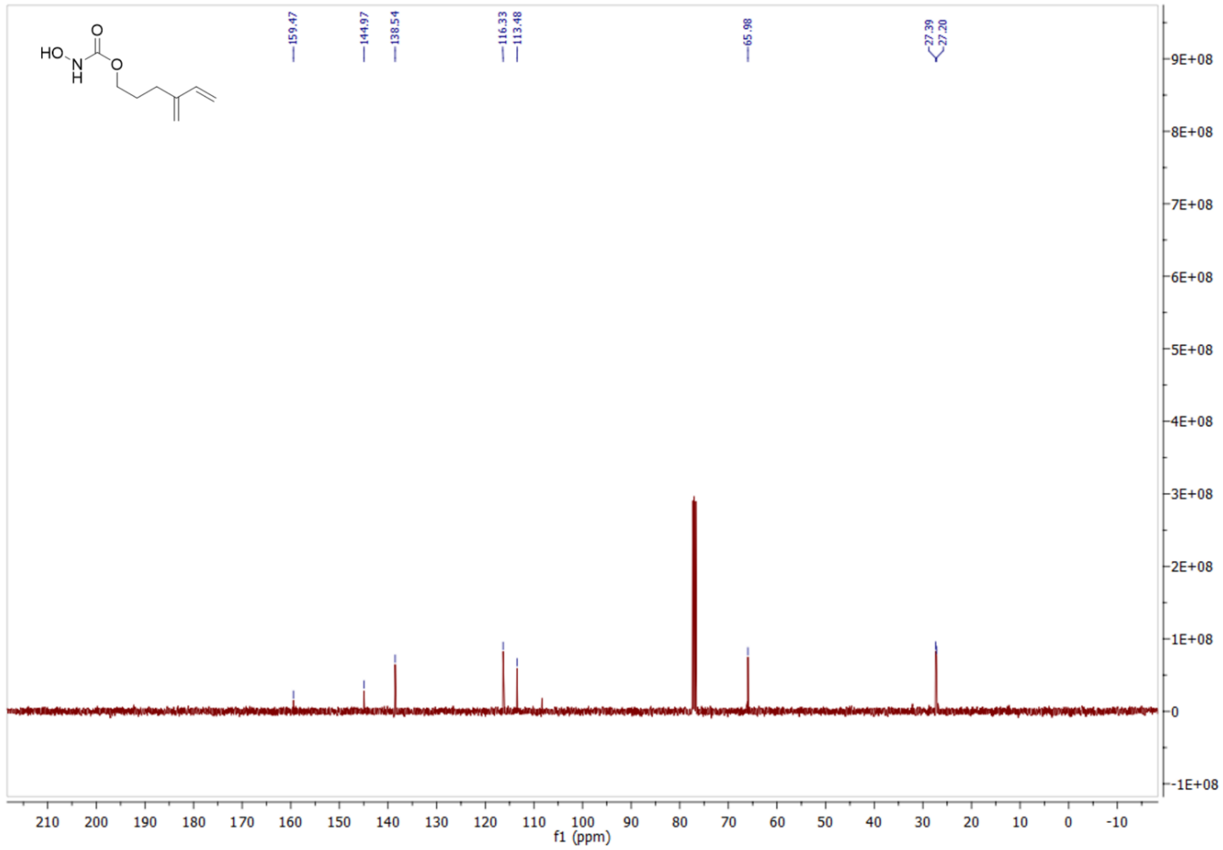

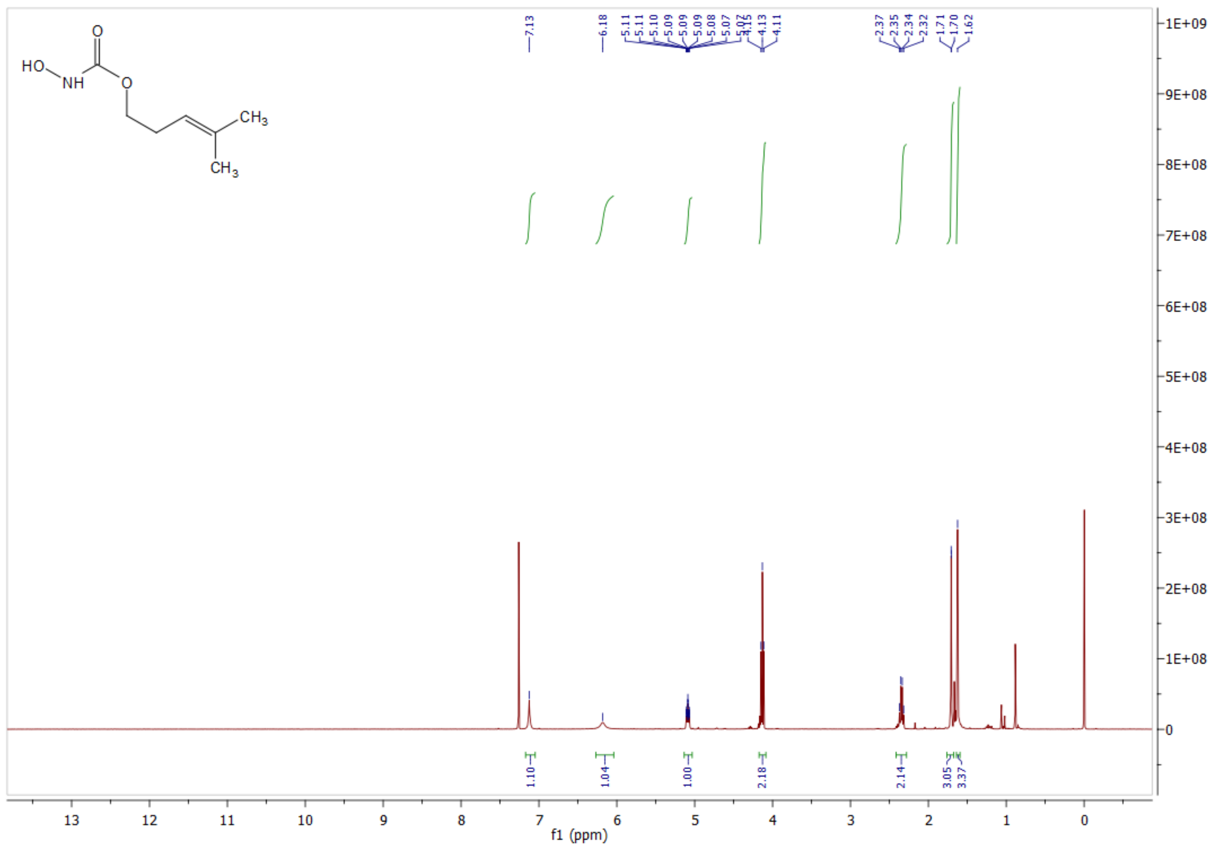

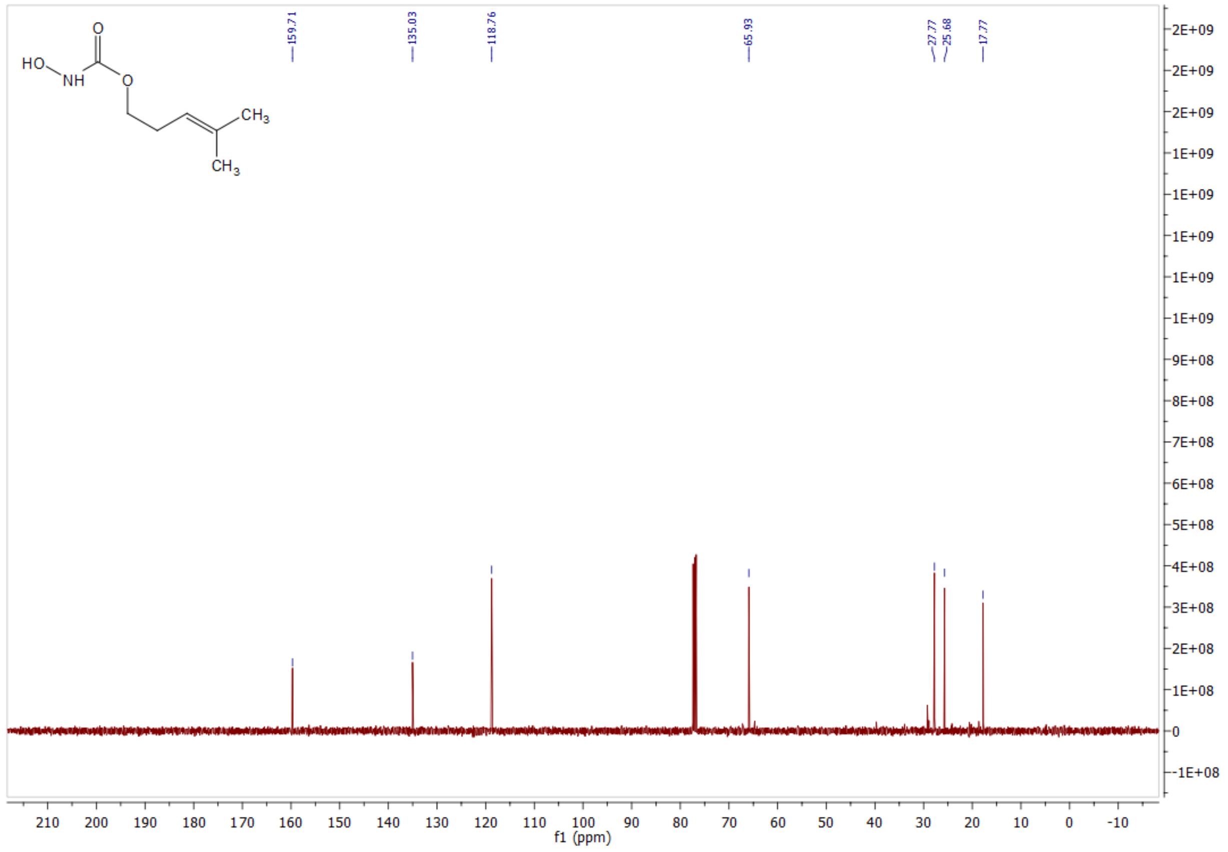

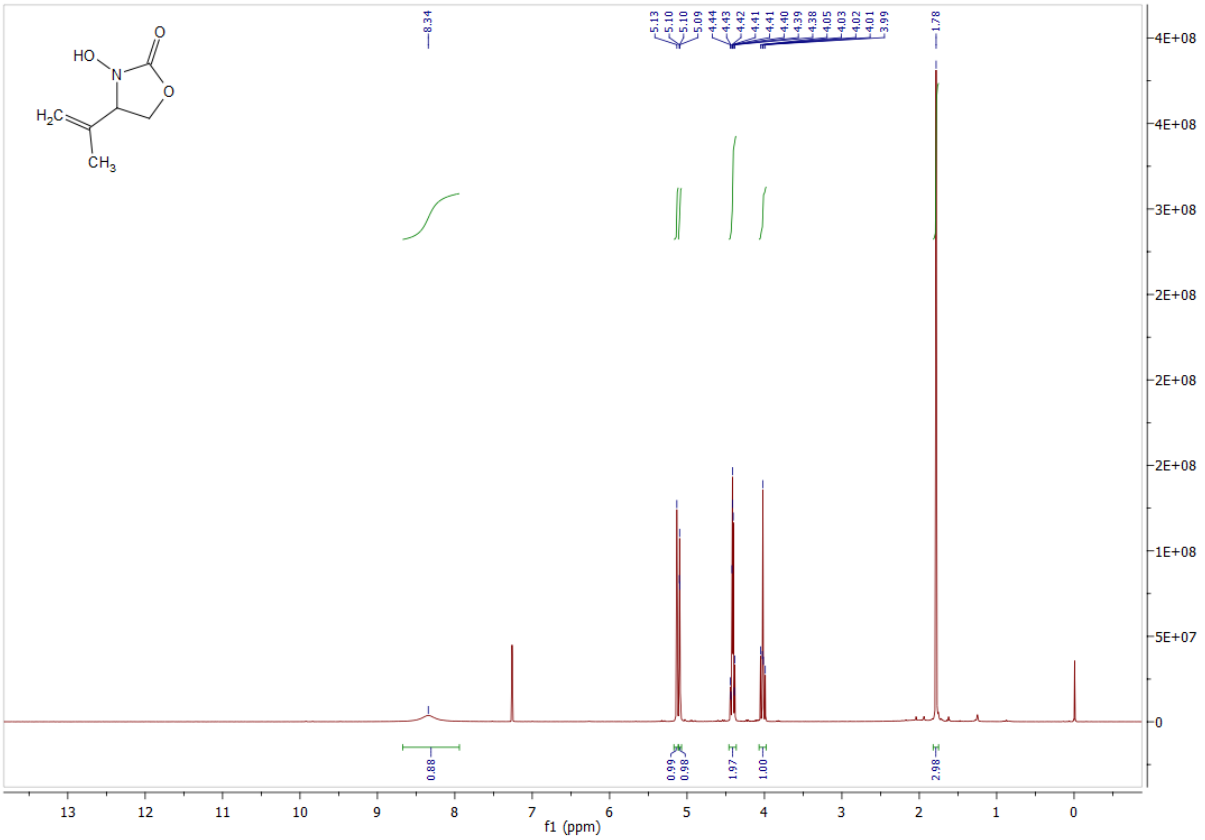
HO
N
O
O
H2C
CH3
8.34
5.13
5.10
5.10
5.09
4.44
4.43
4.42
4.41
4.41
4.40
4.39
4.38
4.05
4.03
4.02
4.01
3.99
1.78
0.88
0.99
0.98
1.97
1.00
2.98
f1 (ppm)

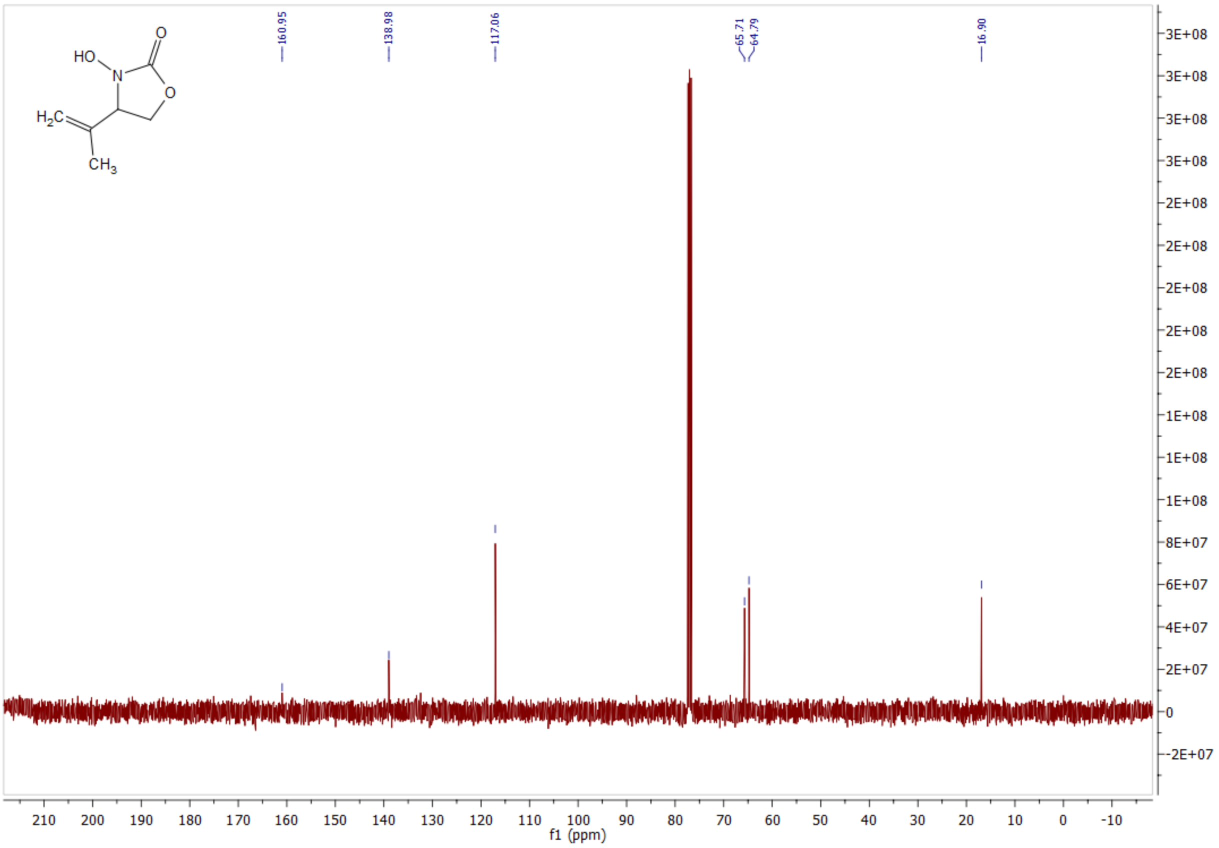
HO
N
O
O
H2C
CH3
160.95
138.98
117.06
65.71
64.79
16.90
f1 (ppm)

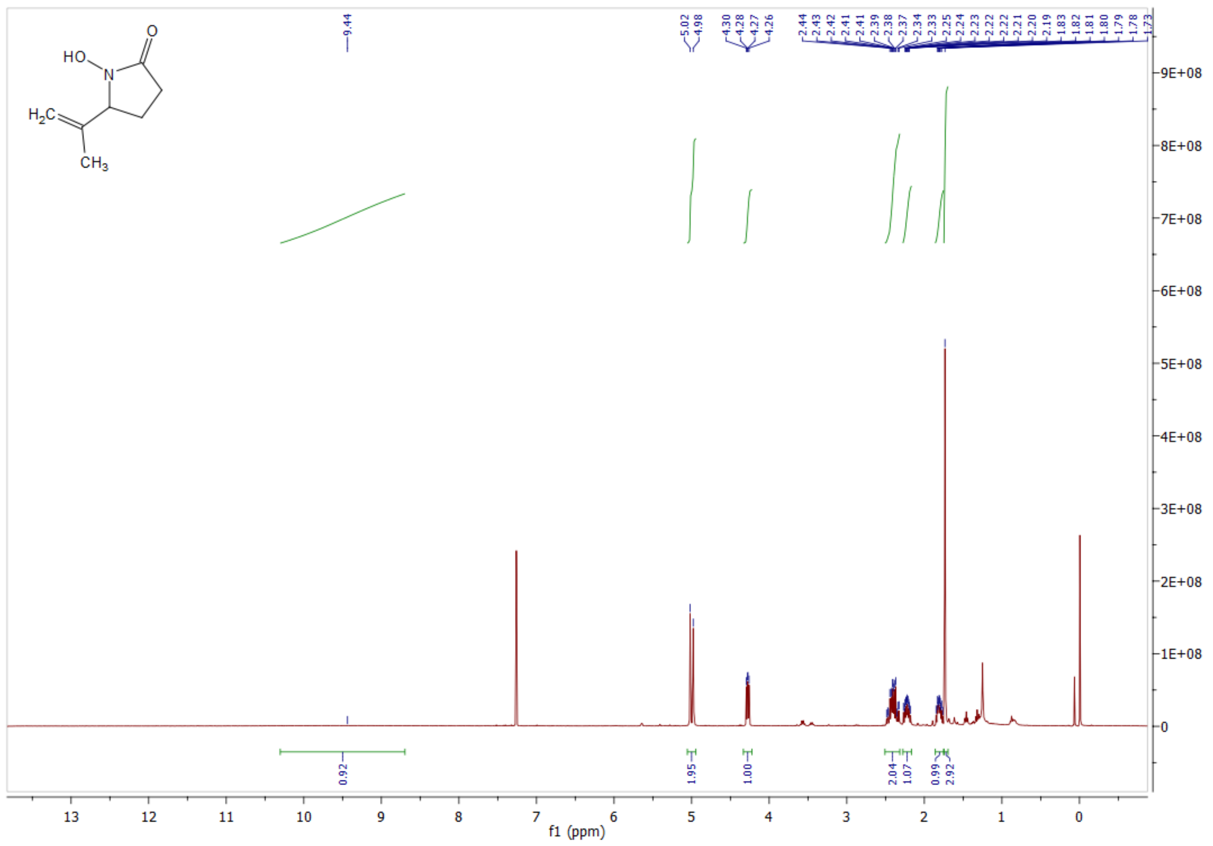
HO
N
O
H2C
CH3
9.44
5.02
4.98
4.30
4.28
4.27
4.26
0.92
1.95
1.00
2.04
1.07
0.99
2.92
f1 (ppm)

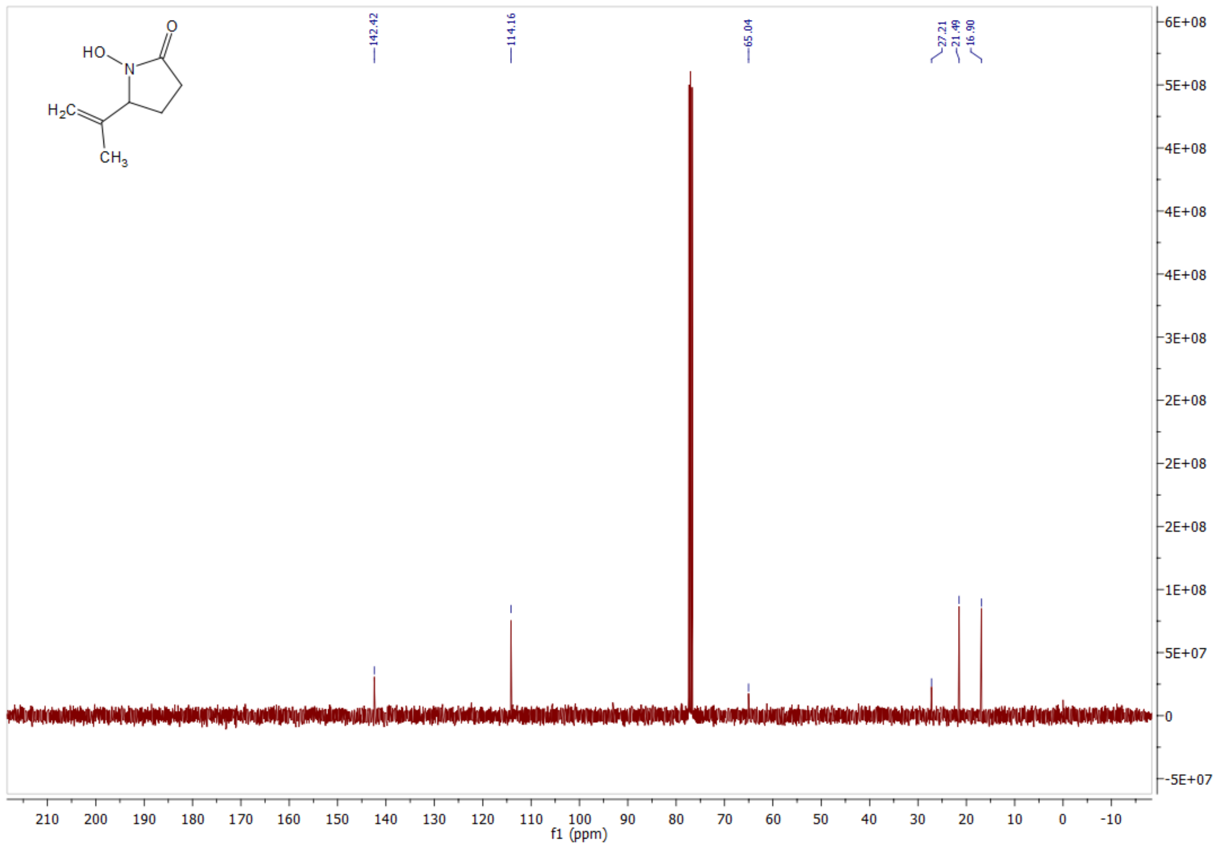
HO
N
O
H2C
CH3
142.42
114.16
65.04
27.21
21.49
16.90
f1 (ppm)

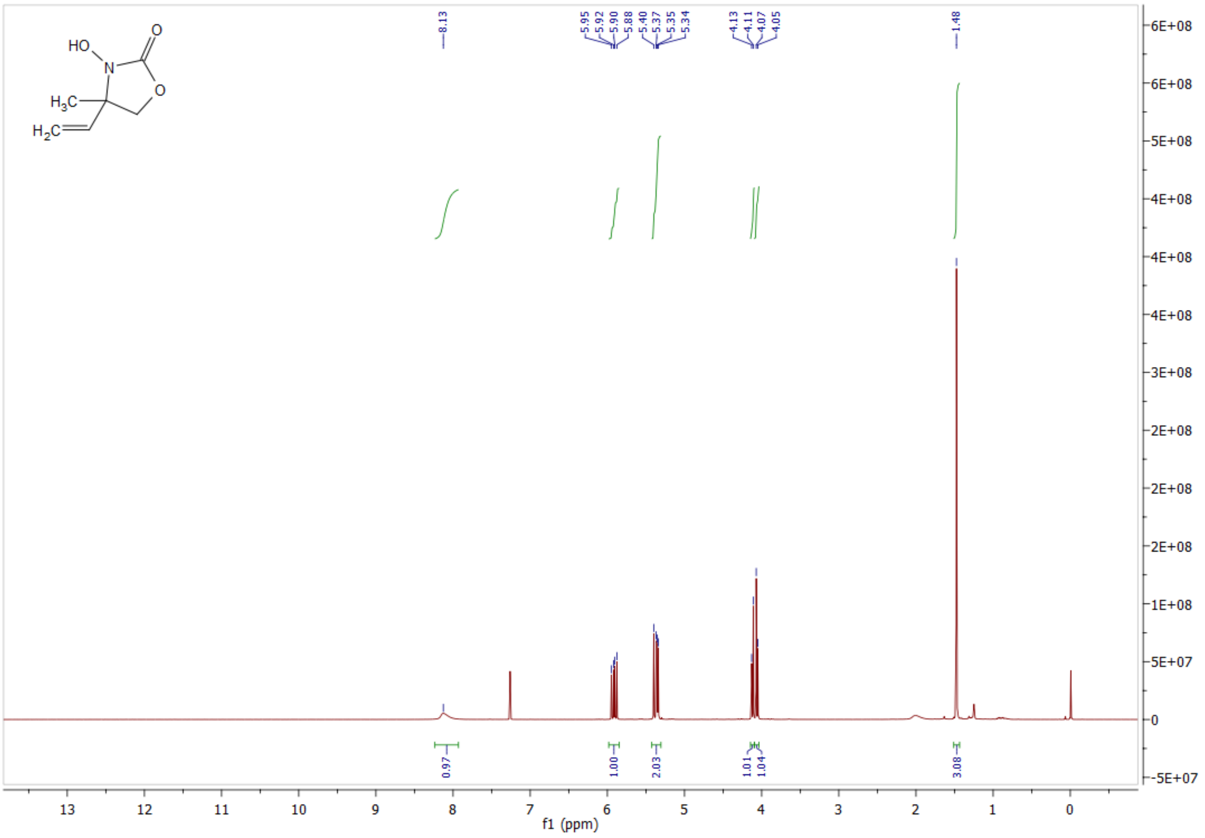
HO
N
O
O
H3C
H2C
8.13
5.95
5.92
5.90
5.88
5.40
5.37
5.35
5.34
4.13
4.11
4.07
4.05
1.48
0.97
1.00
2.03
1.01
1.04
3.08
f1 (ppm)

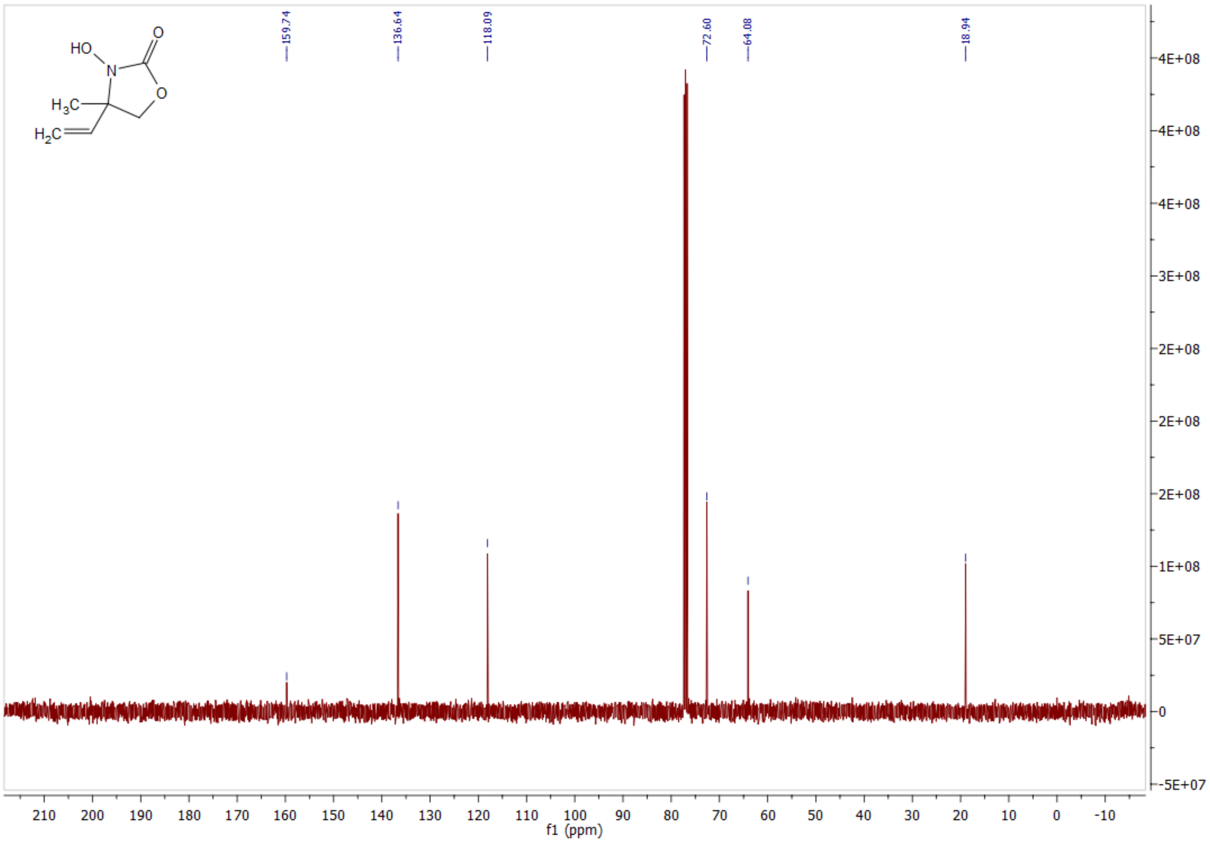
HO
N
O
O
H3C
H2C
159.74
136.64
118.09
72.60
64.08
18.94
f1 (ppm)

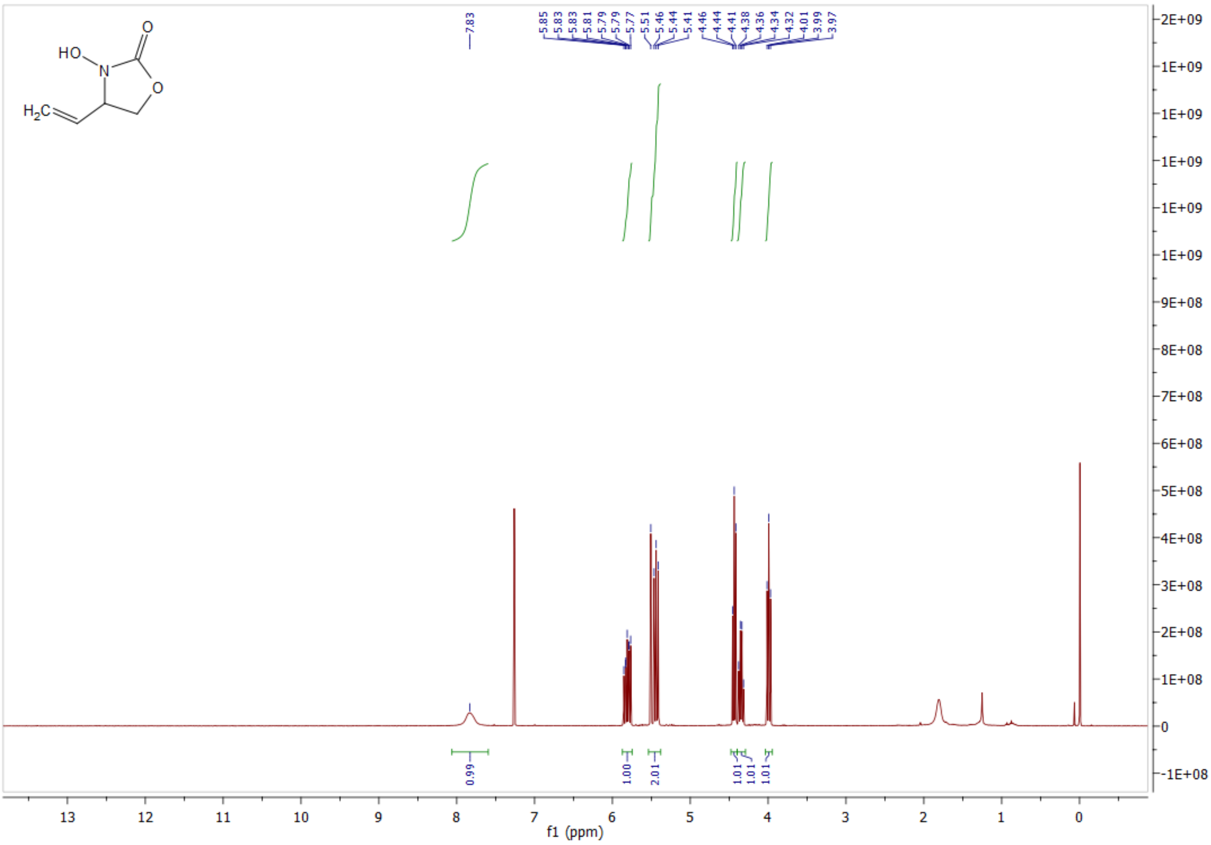
HO
N
O
O
H2C
7.83
5.85
5.83
5.83
5.81
5.79
5.79
5.77
5.51
5.46
5.44
5.41
4.46
4.44
4.41
4.38
4.36
4.34
4.32
4.01
3.99
3.97
0.99
1.00
2.01
1.01
1.01
1.01
f1 (ppm)

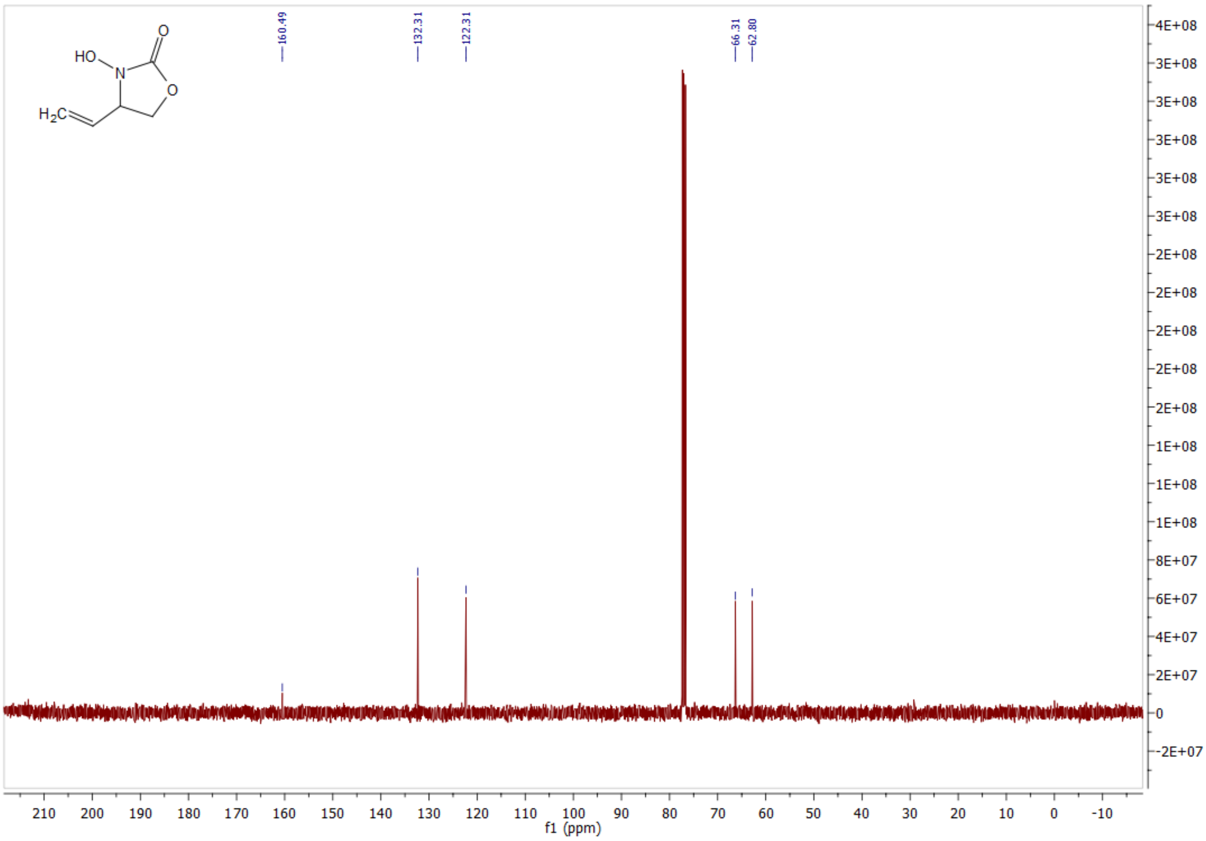
HO
N
O
O
H2C
160.49
132.31
122.31
66.31
62.80
f1 (ppm)

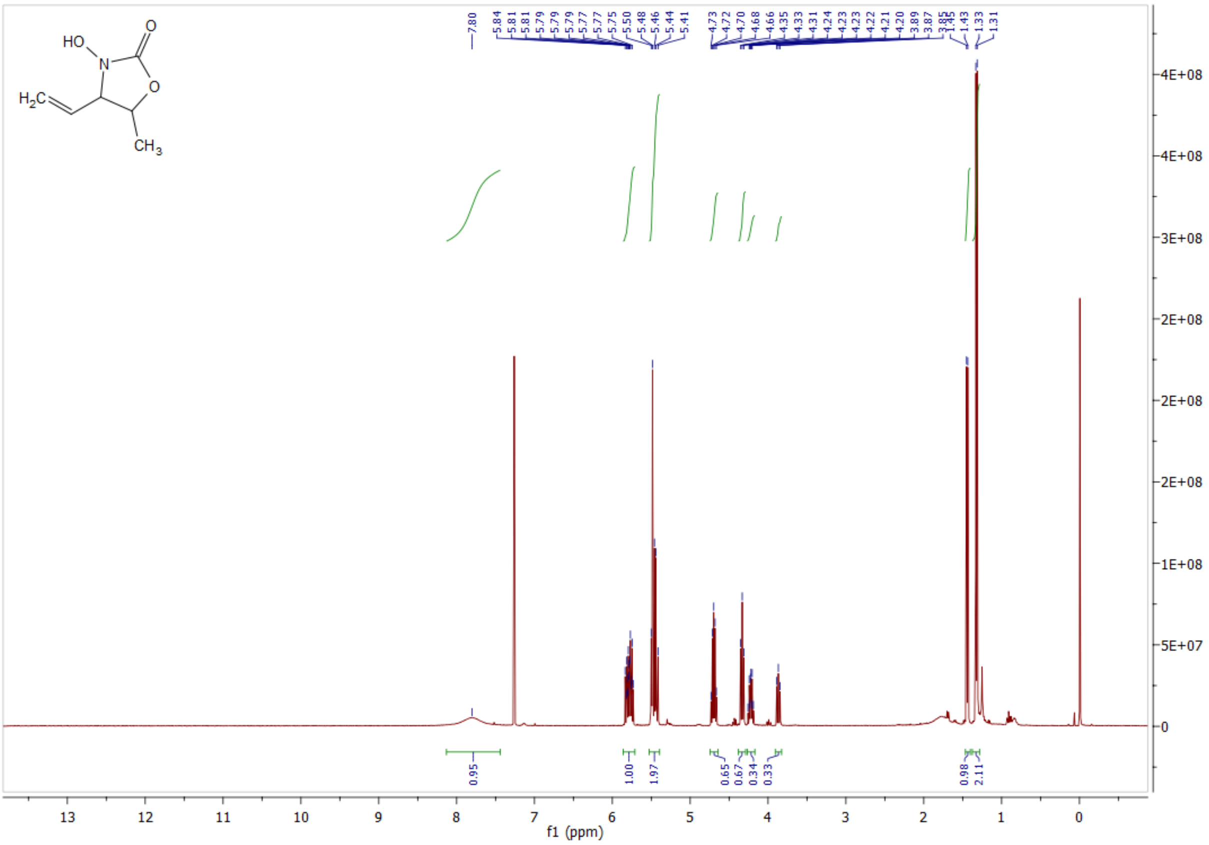
HO
N
O
O
H2C
CH3
7.80
5.84
5.81
5.81
5.79
5.79
5.79
5.77
5.77
5.75
5.50
5.48
5.46
5.44
5.41
4.73
4.72
4.70
4.68
4.66
4.35
4.33
4.31
4.24
4.23
4.23
4.22
4.21
4.20
3.89
3.87
3.85
1.45
1.43
1.33
1.31
0.95
1.00
1.97
0.65
0.67
0.34
0.33
0.98
2.11
f1 (ppm)

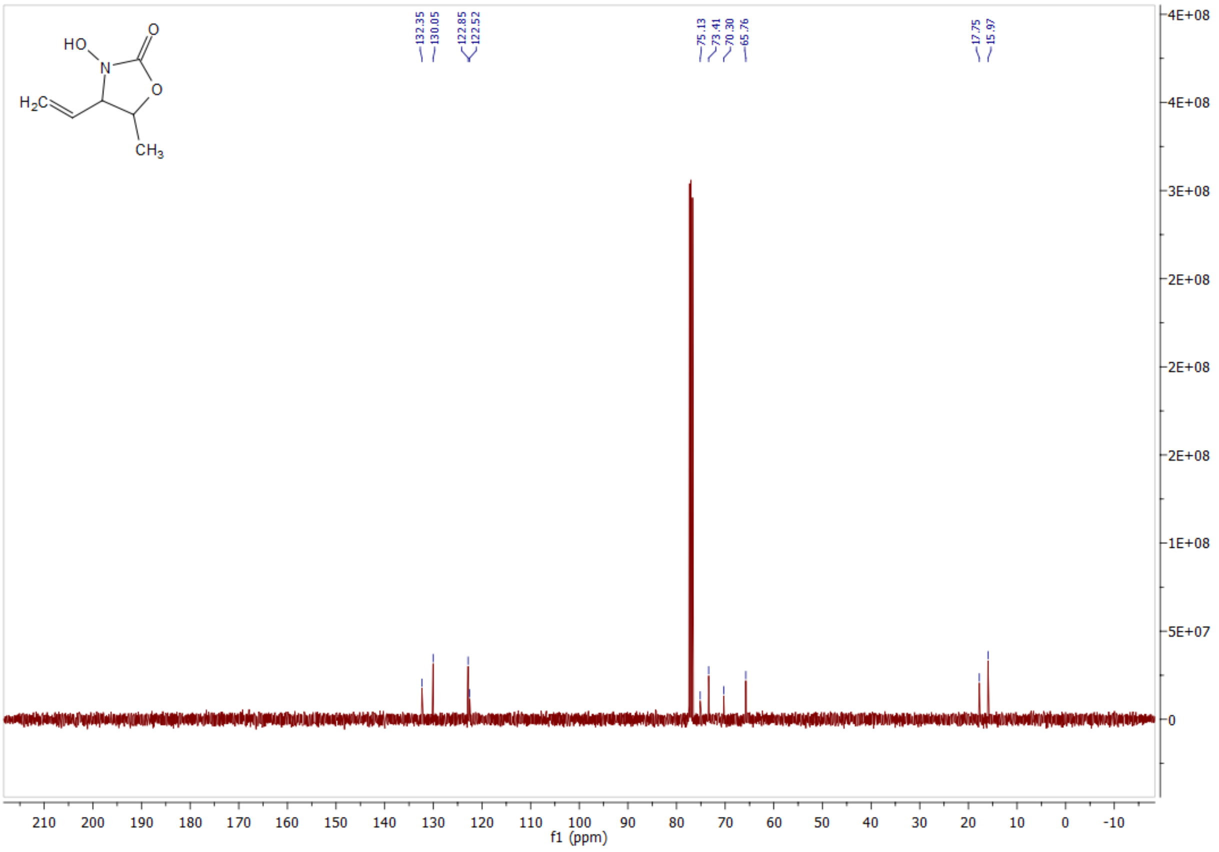
HO
N
O
O
H2C
CH3
132.35
130.05
122.85
122.52
75.13
73.41
70.30
65.76
17.75
15.97
f1 (ppm)

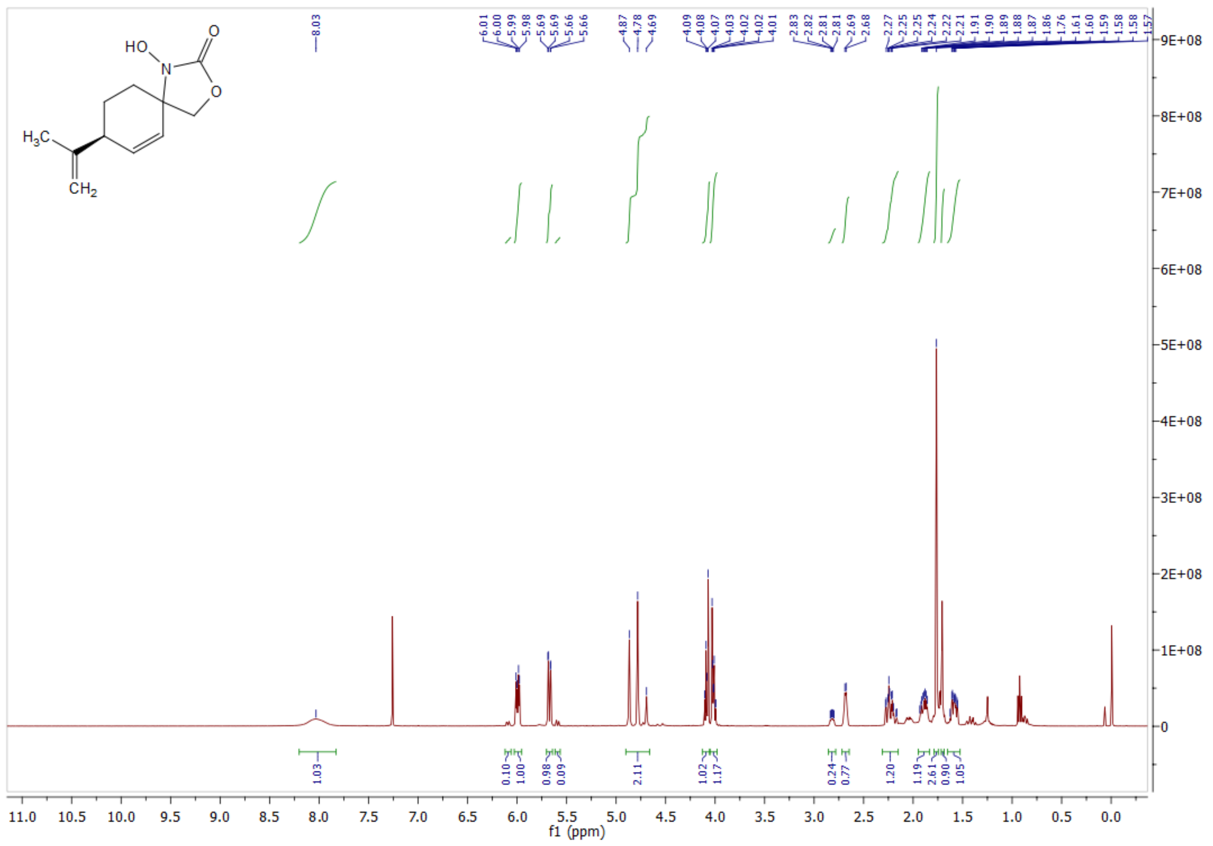

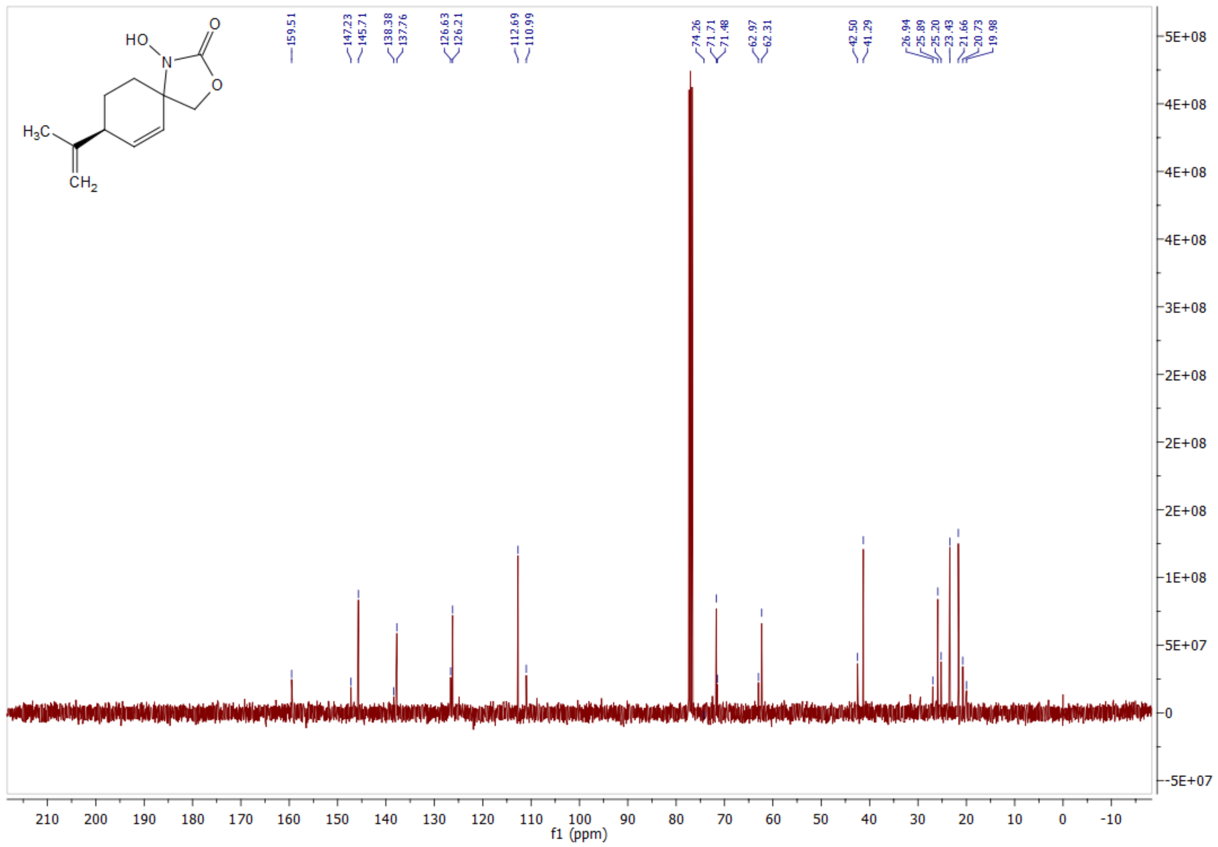

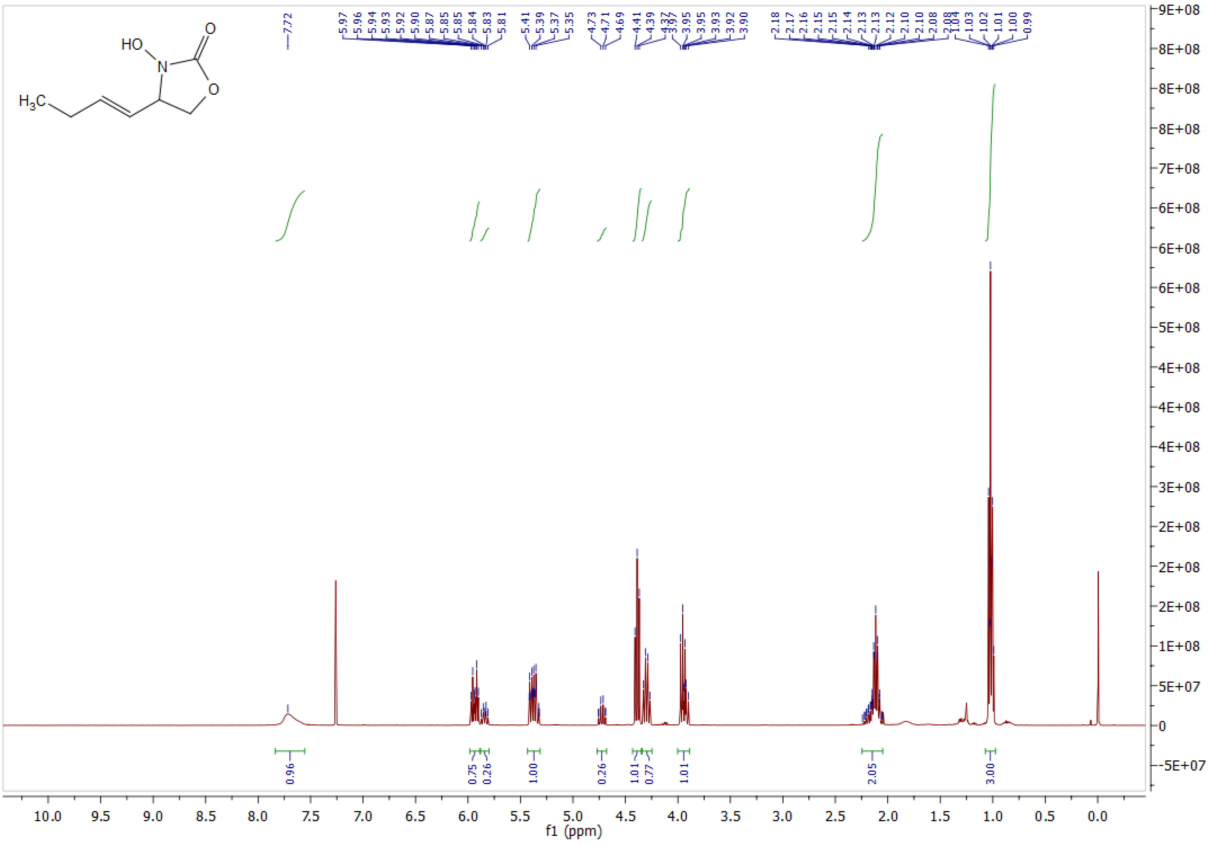
HO
N
O
O
H3C
7.72
0.96
0.75
0.26
1.00
0.26
1.01
0.77
1.01
2.05
3.00
f1 (ppm)

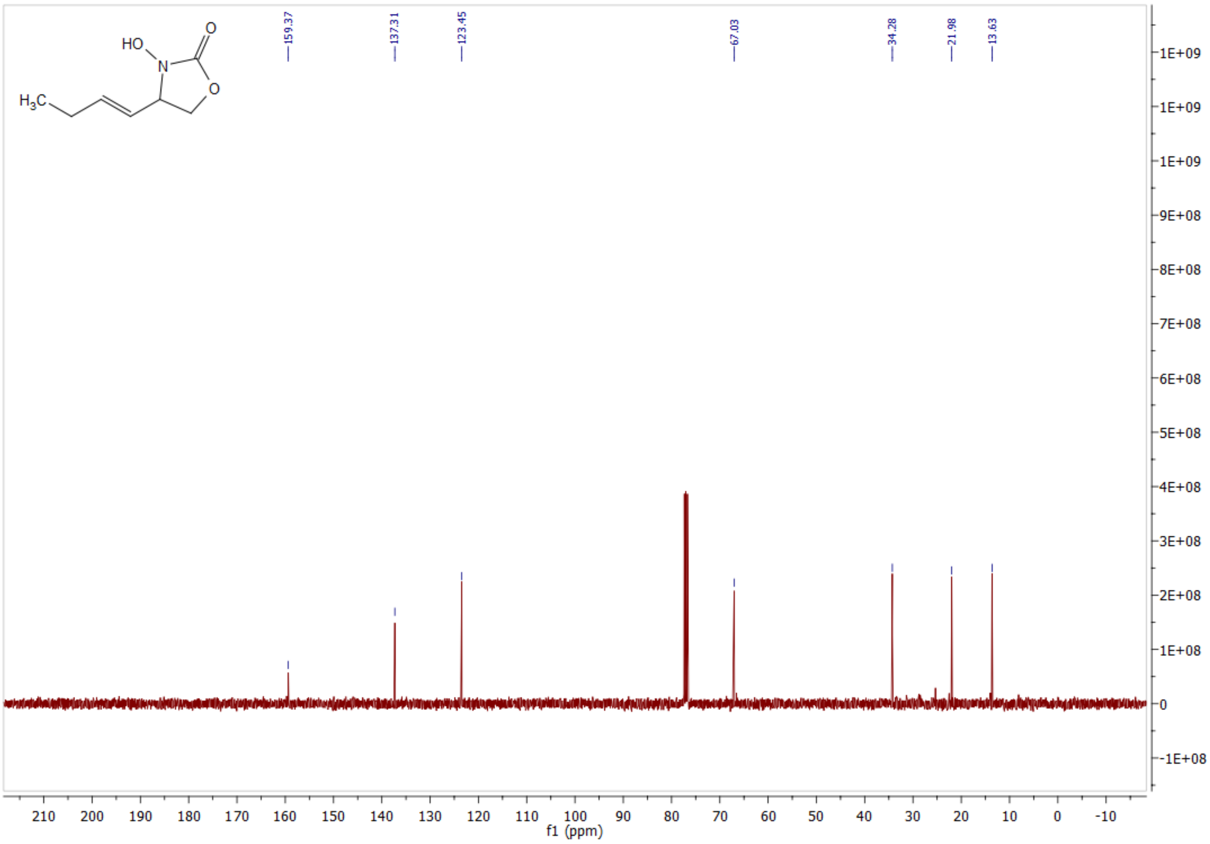
HO
N
O
O
H3C
159.37
137.31
123.45
67.03
34.28
21.98
13.63
f1 (ppm)

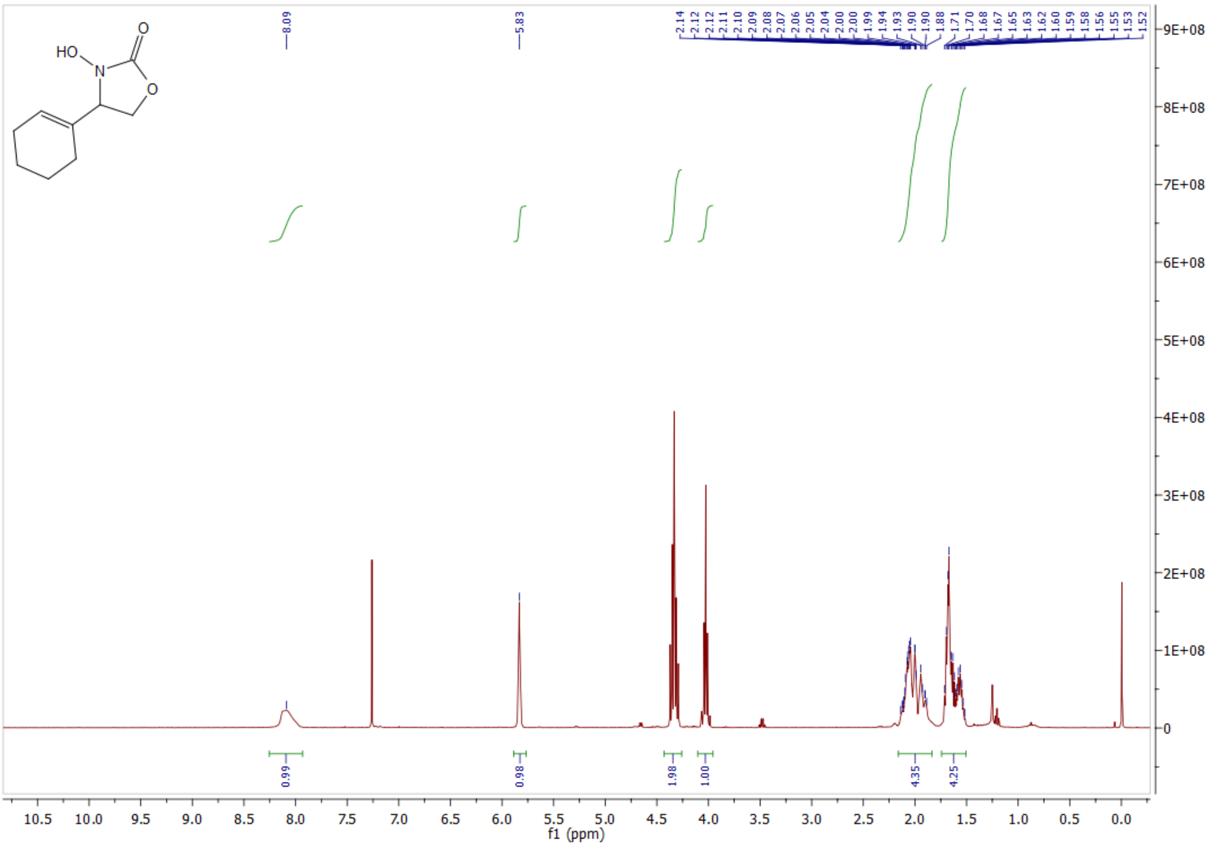
HO
N
O
O
8.09
5.83
2.14 2.12 2.12 2.11 2.10 2.09 2.08 2.07 2.06 2.05 2.04 2.00 2.00 1.99 1.94 1.93 1.90 1.90 1.88 1.71 1.70 1.68 1.67 1.65 1.63 1.62 1.60 1.59 1.58 1.56 1.55 1.53 1.52
0.99
0.98
1.98
1.00
4.35
4.25
f1 (ppm)

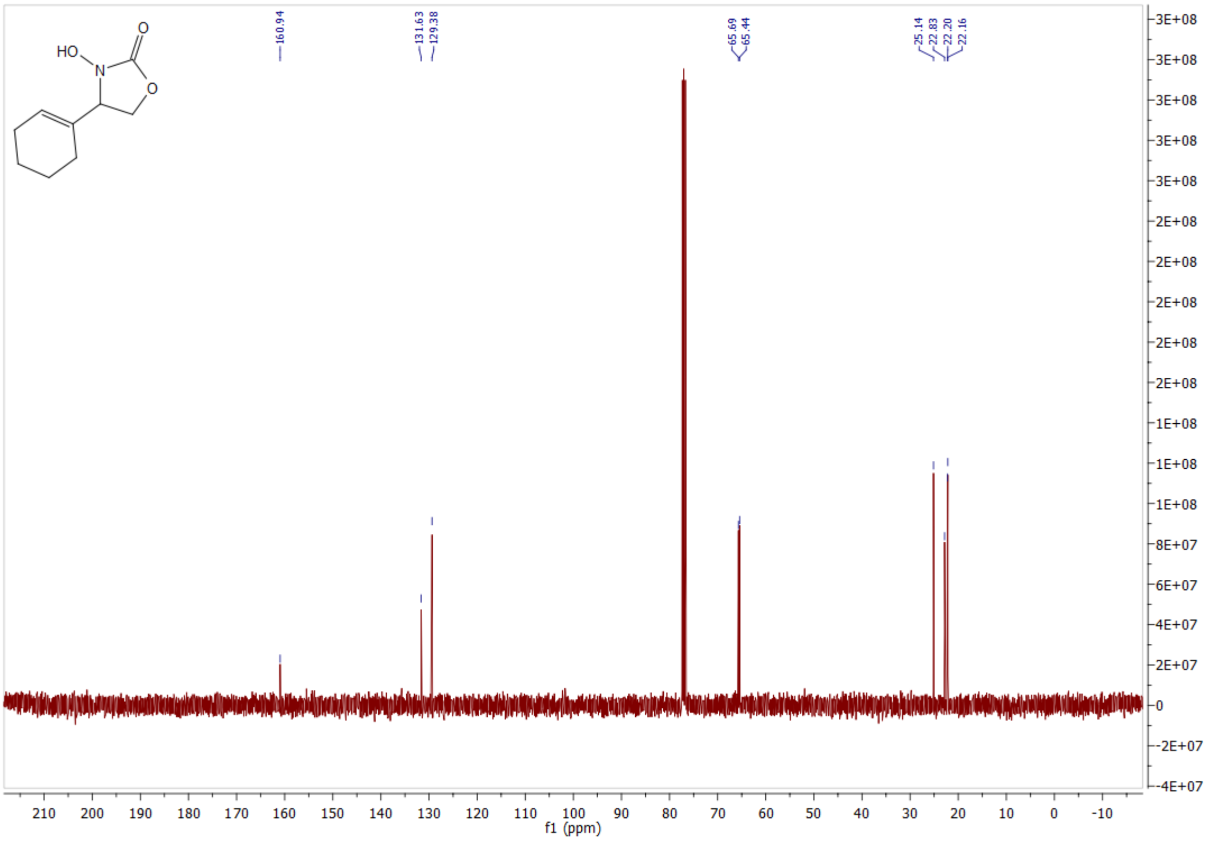
HO
N
O
O
160.94
131.63
129.38
65.69
65.44
25.14
22.83
22.20
22.16
f1 (ppm)

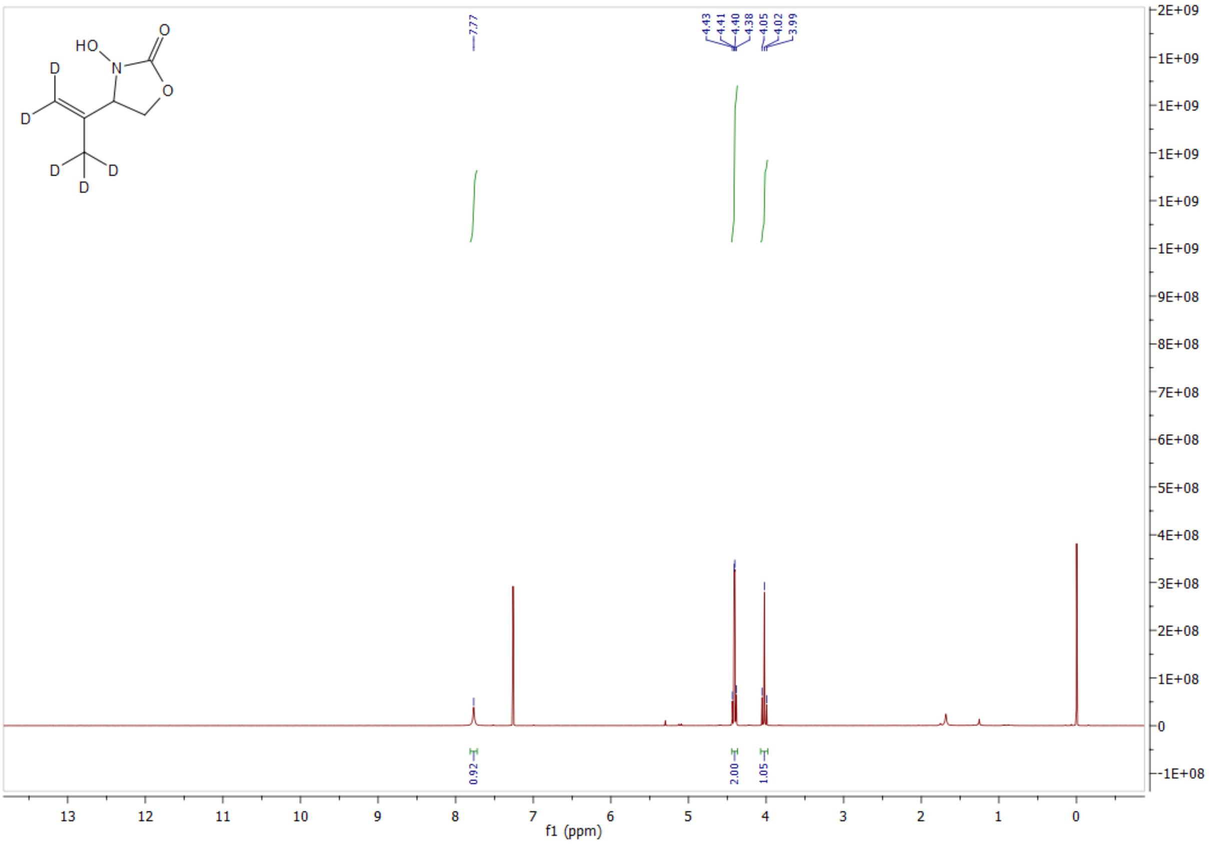
HO
N
O
O
D
D
D
D
D
7.77
4.43
4.41
4.40
4.38
4.05
4.02
3.99
0.92
2.00
1.05
f1 (ppm)

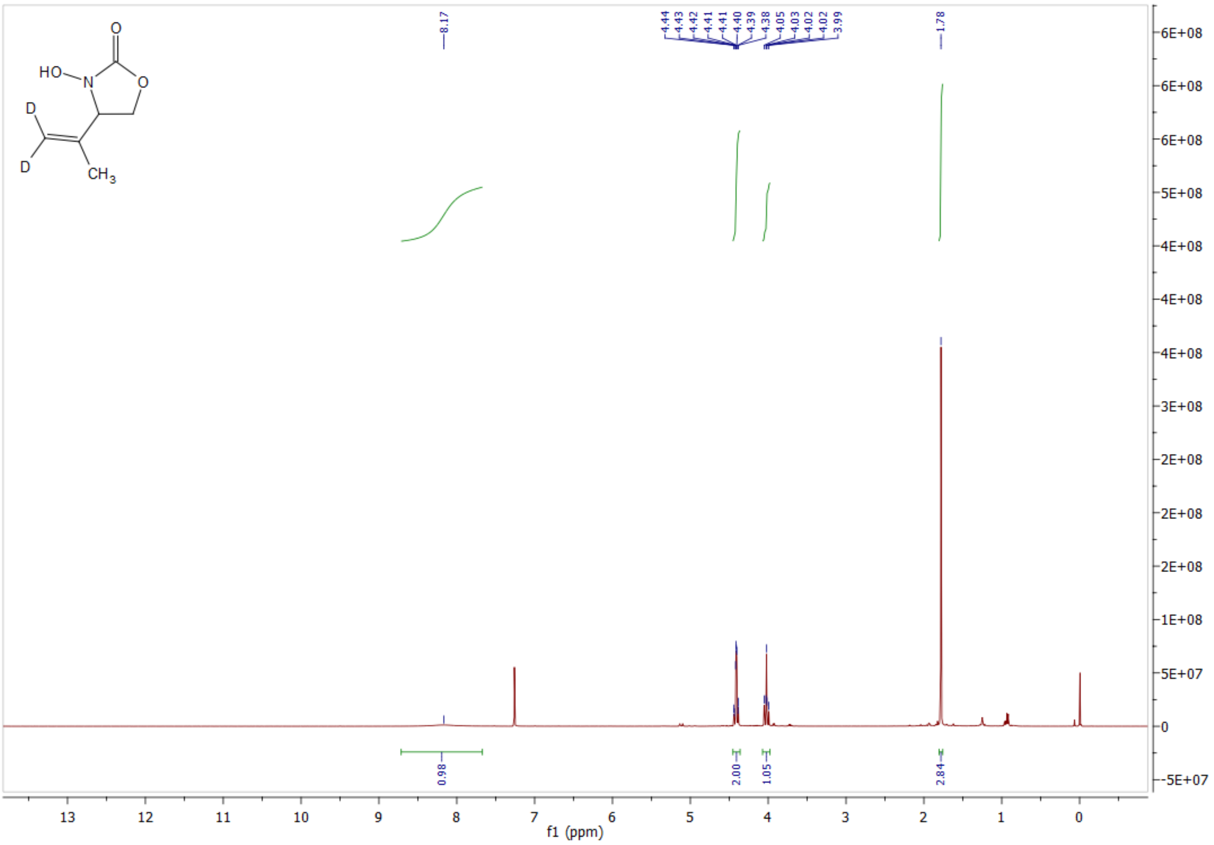
HO
N
O
O
D
D
CH3
8.17
4.44
4.43
4.42
4.41
4.41
4.40
4.39
4.38
4.05
4.03
4.02
4.02
3.99
1.78
0.98
2.00
1.05
2.84
f1 (ppm)

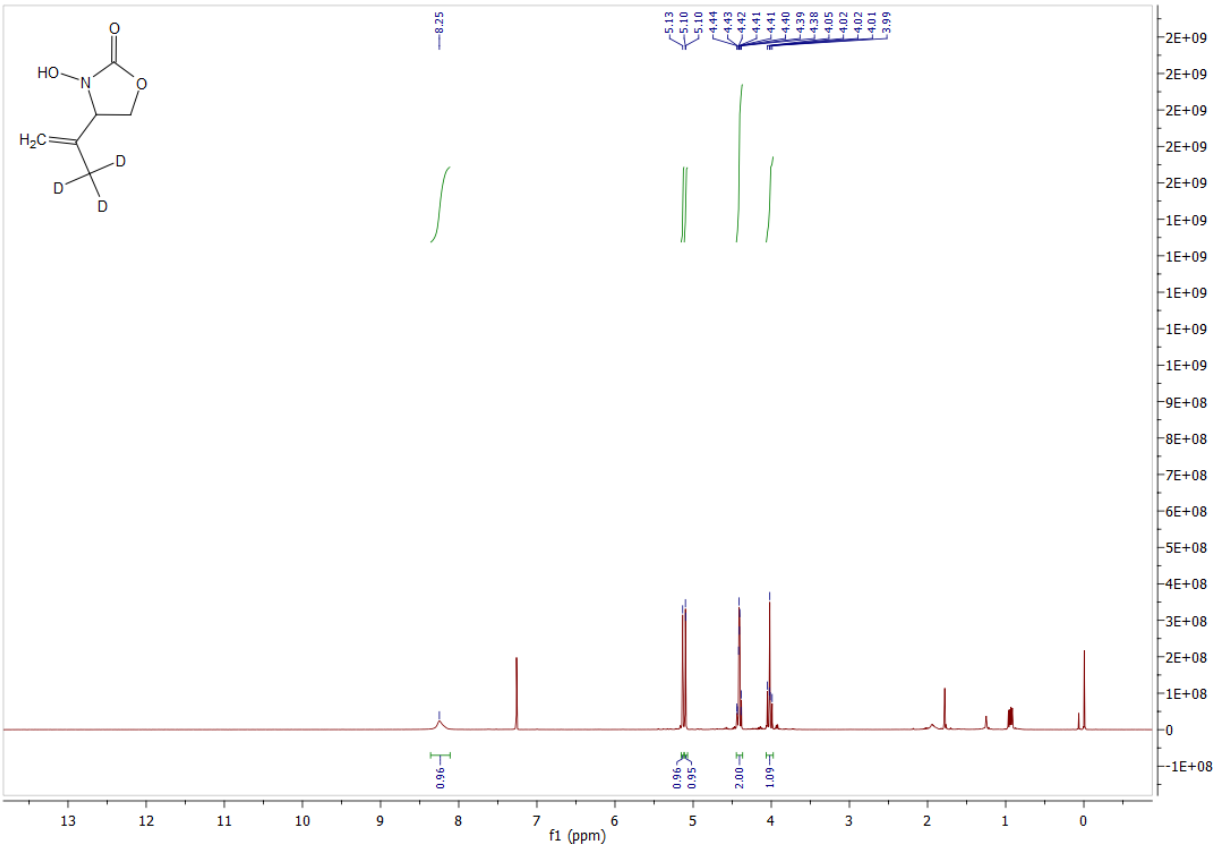

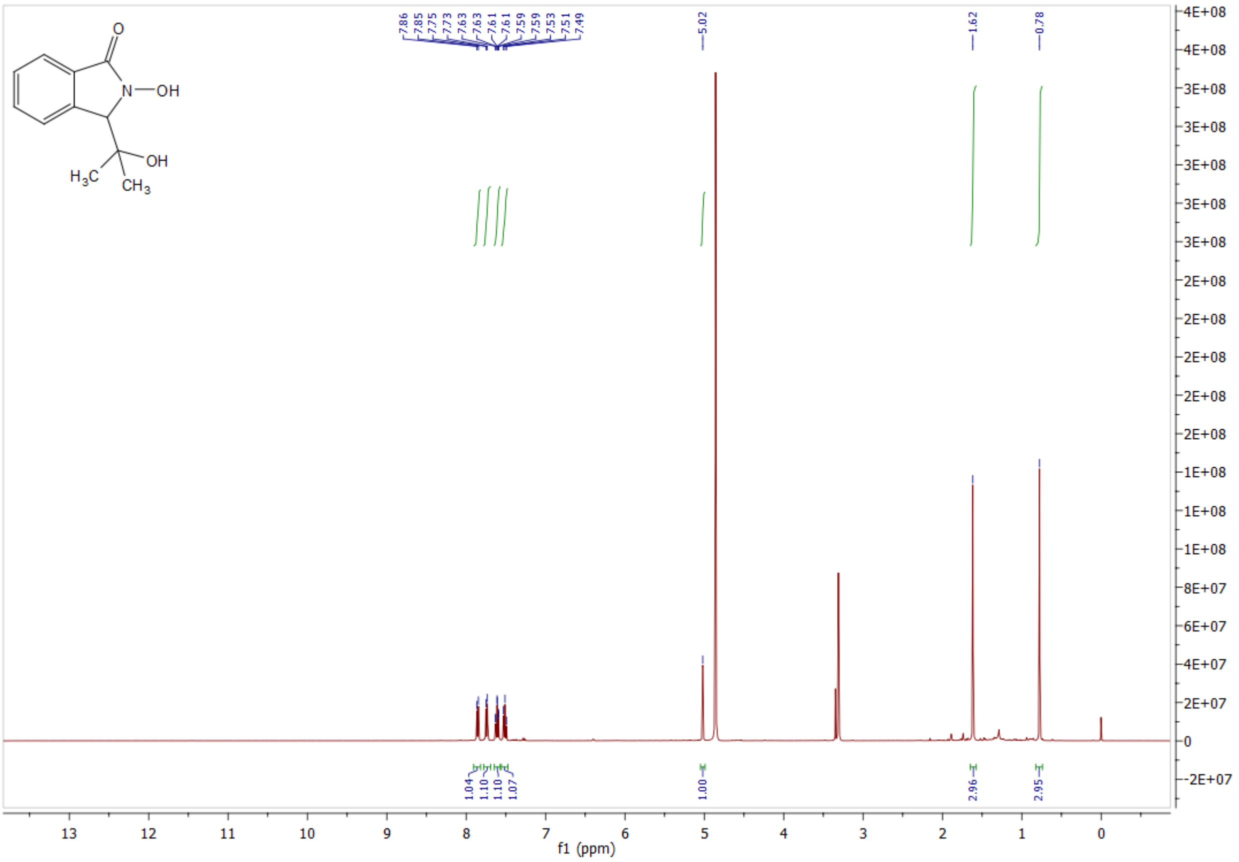
O
N—OH
OH
H3C
CH3
7.86
7.85
7.75
7.73
7.63
7.63
7.61
7.61
7.59
7.59
7.53
7.51
7.49
5.02
1.62
0.78
1.04
1.10
1.10
1.07
1.00
2.96
2.95
f1 (ppm)

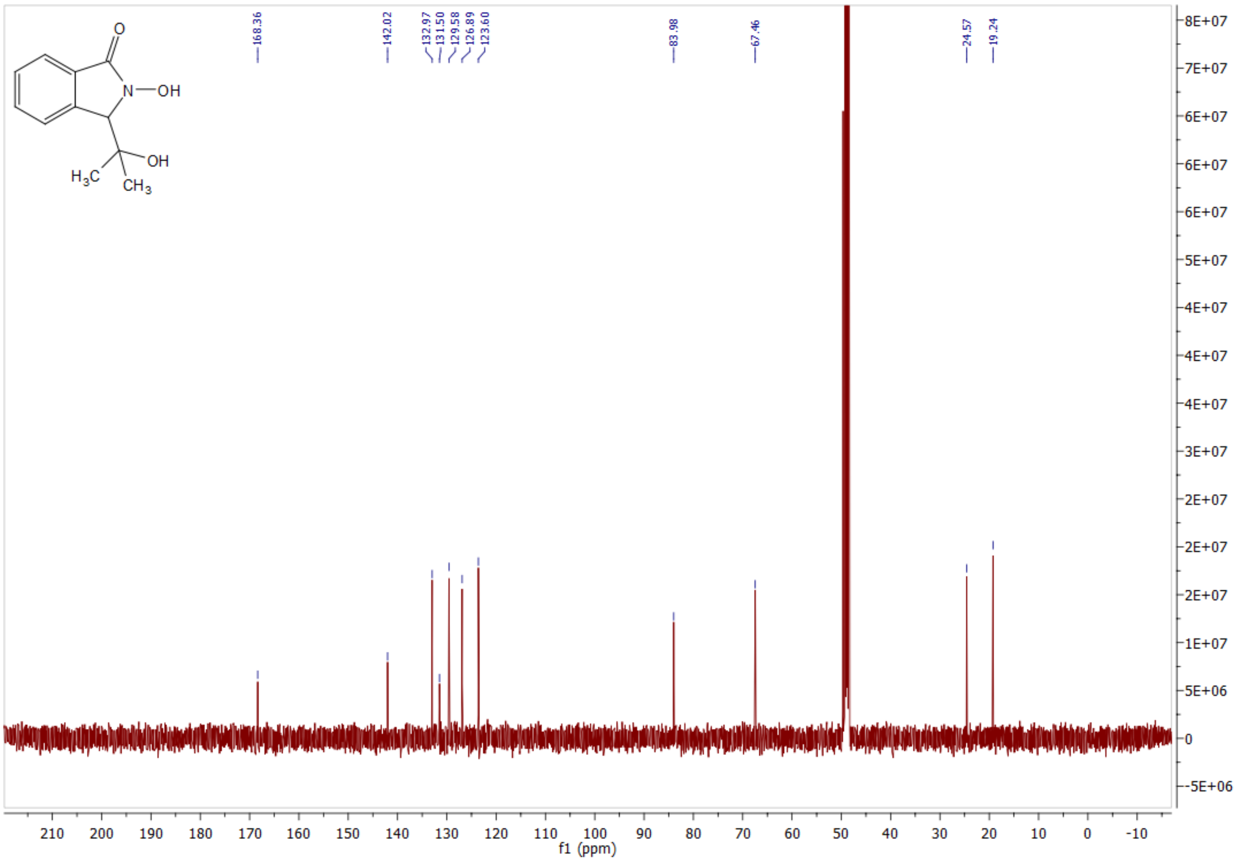
O
N—OH
OH
H3C
CH3
168.36
142.02
132.97
131.50
129.58
126.89
123.60
83.98
67.46
24.57
19.24
f1 (ppm)

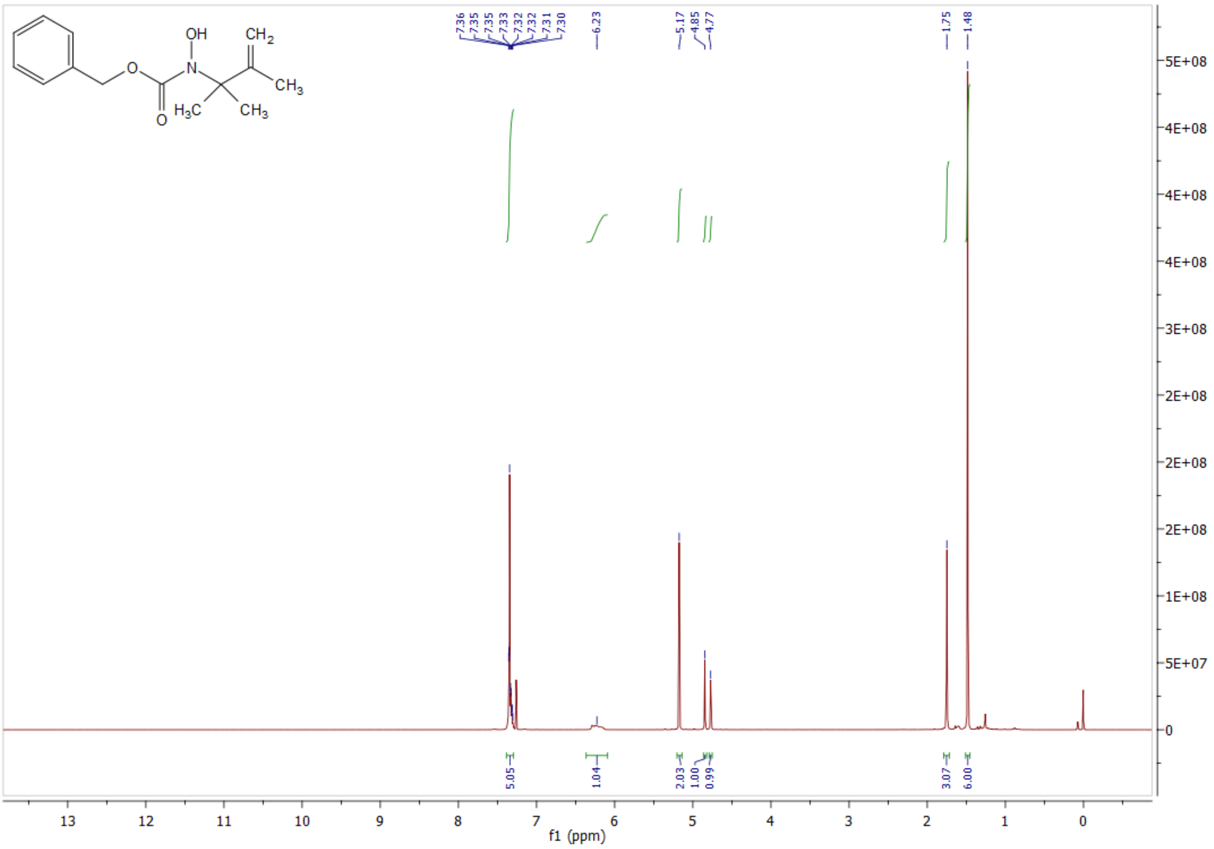
OH
CH2
CH3
H3C
CH3
O
N
7.36
7.35
7.35
7.33
7.32
7.32
7.31
7.30
6.23
5.17
4.85
4.77
1.75
1.48
5.05
1.04
2.03
1.00
0.99
3.07
6.00
f1 (ppm)

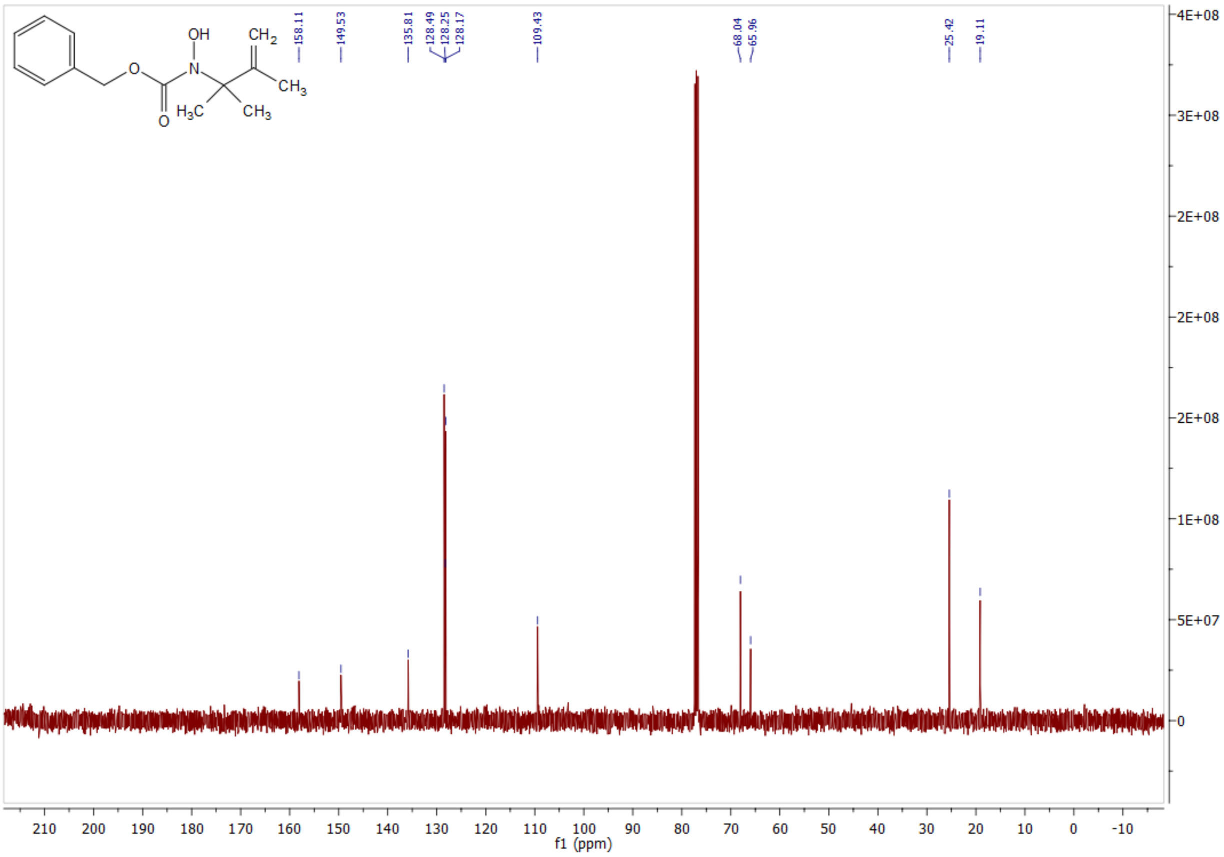
OH
CH2
CH3
H3C
CH3
O
N
158.11
149.53
135.81
128.49
128.25
128.17
109.43
68.04
65.96
25.42
19.11
f1 (ppm)

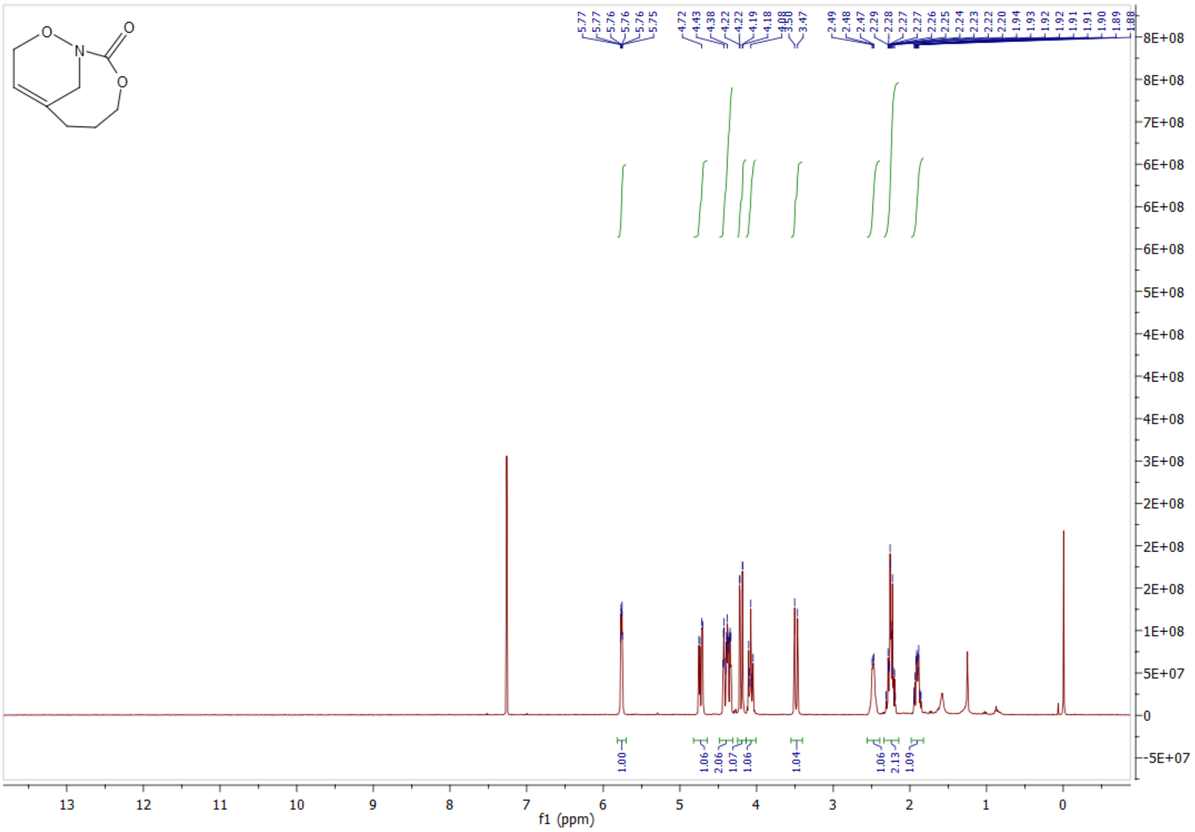
5.77
5.77
5.76
5.76
5.76
5.75
4.72
4.43
4.38
4.22
4.22
4.19
4.18
4.08
3.50
3.47
2.49
2.48
2.47
2.29
2.28
2.27
2.27
2.26
2.25
2.24
2.23
2.22
2.20
1.94
1.93
1.92
1.92
1.91
1.91
1.90
1.89
1.88
1.00
1.06
2.06
1.07
1.06
1.04
1.06
2.13
1.09
f1 (ppm)

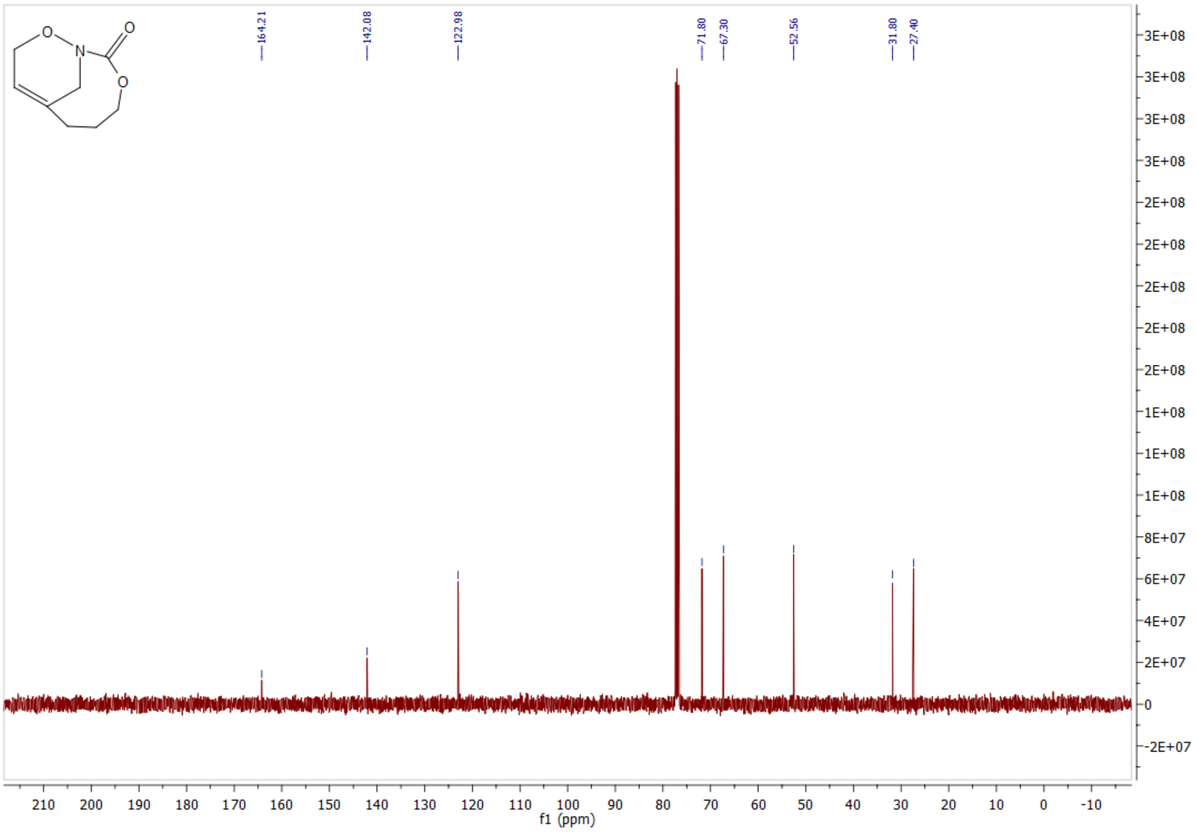
164.21
142.08
122.98
71.80
67.30
52.56
31.80
27.40
f1 (ppm)

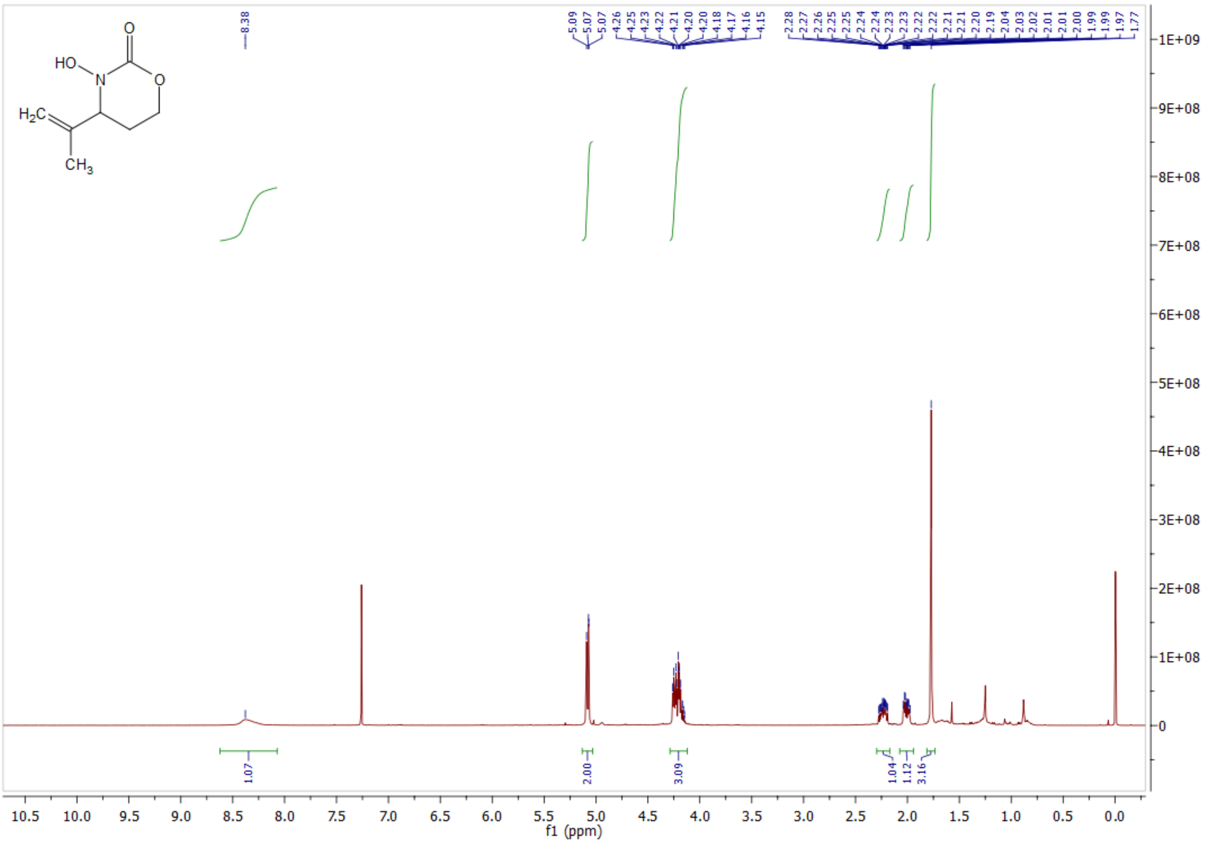
8.38
5.09
5.07
5.07
4.26
4.25
4.23
4.22
4.21
4.20
4.18
4.17
4.16
4.15
1.07
2.00
3.09
1.04
1.12
3.16
f1 (ppm)

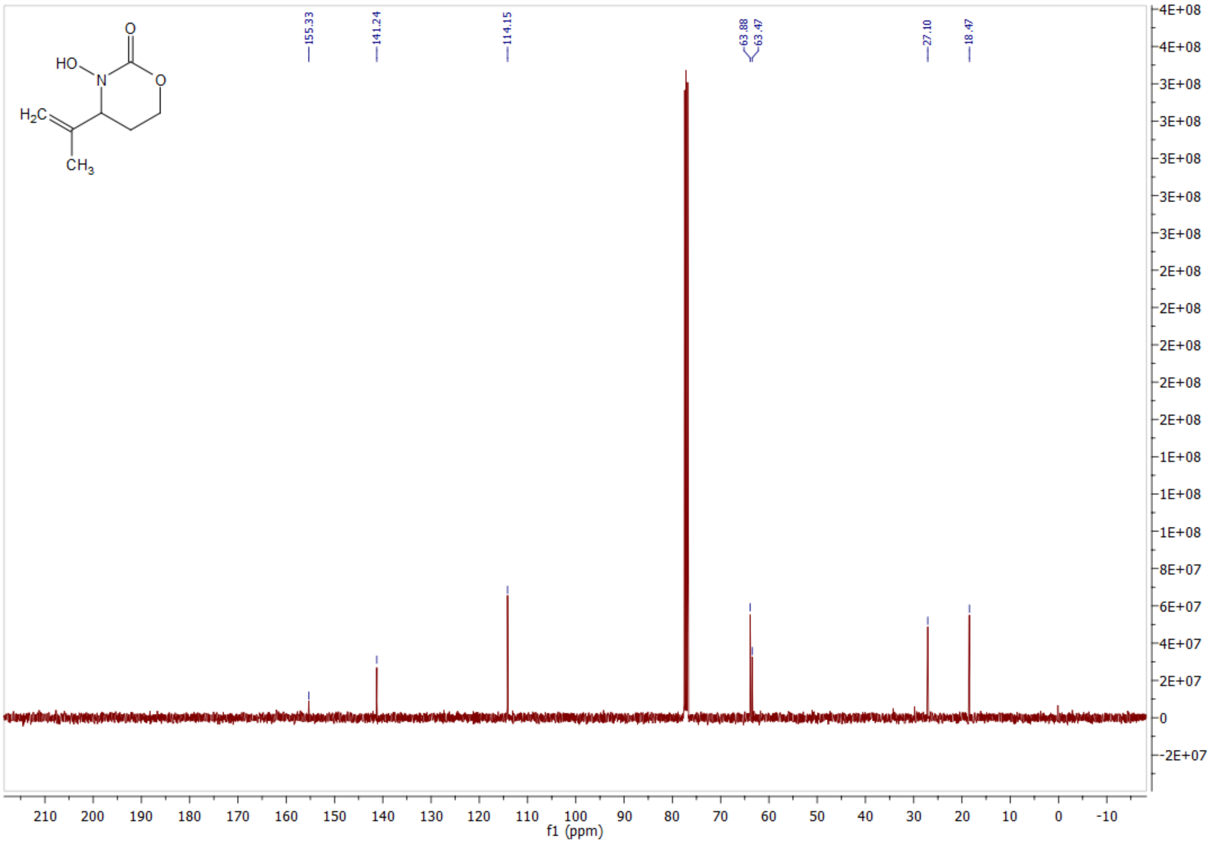
155.33
141.24
114.15
63.88
63.47
27.10
18.47
f1 (ppm)

Supplementary References

[1] P. Lopes, K. Koschorreck, J. N. Pedersen, A. Ferapontov, S. Lorcher, J. S. Pedersen, V. B. Urlacher, E. E. Ferapontova, *ChemElectroChem* **2019**, *6*, 2043-2049.

[2] K. Koschorreck, S. M. Richter, A. Swierczek, U. Beifuss, R. D. Schmid, V. B. Urlacher, *Arch. Biochem. Biophys*. **2008**, *474*, 213-219.

[3] A. Bronikowski, P. L. Hagedoorn, K. Koschorreck, V. B. Urlacher, *AMB Express* **2017**, *7*. 73.

[4] M. Gunne, V. B. Urlacher, *PloS one* **2012**, *7*, e52360.

[5] R. Liu, S. R. Herron, S. A. Fleming, *J. Org. Chem.* **2007**, *72*, 5587-5591.

[6] C. P. Frazier, J. R. Engelking, J. Read de Alaniz, *J. Am. Chem. Soc*. **2011**, *133*,10430-10433.

[7] H. Lebel, K. Huard, S. Lectard, *J. Am. Chem. Soc*. **2005**, *127*,14198-14199.

[8] S. Y. Hong, S. Chang, *J. Am. Chem. Soc*. **2019**, *141*, 10399-10408.

[9] D. Atkinson, M. A. Kabeshov, M. Edgar, A. V. Malkov, *Adv. Synth. Catal*. **2011**, *353*, 3347-3351.

[10] D. Konrádová, H. Kozubíková, K. Doležal, J. Pospíšil, *Eur. J. Org. Chem*. **2017**, 5204-5213.

[11] R. O. McCourt, E. M. Scanlan, *Org. Lett*. **2019**, *21*, 3460-3464.

[12] S.-R. Choi, M. Breugst, K. N. Houk, C. D. Poulter, *J. Org. Chem*. **2014**, *79*, 3572-3580.

[13] D. A. Candito, D. Dobrovolsky, M. Lautens, *J. Am. Chem. Soc.* **2012**, *134*, 15572-15580.

[14] J. Y. Wu, B. N. Stanzl, T. Ritter, *J. Am. Chem. Soc.* **2010**, *132*, 13214-13216.

[15] C. P. Chow, K. J. Shea, *J. Am. Chem. Soc*. **2005**, *127*, 3678-3679.

[16] G. E. Keck, R. R. Webb, J. B. Yates, *Tetrahedron* **1981**, *37*, 4007-4016.

[17] D. Chaiyaveij, L. Cleary, A. S. Batsanov, T. B. Marder, K. J. Shea, A. Whiting, *Org. Lett*. **2011**, *13*, 3442-3445.

[18] A. J. Mueller, M. P. Jennings, *Org. Lett*. **2007**, *9*, 5327-5329.

[19] C. Morill, R. H. Grubbs, *J. Am. Chem. Soc*. **2005**, *127*, 2842-2843.

[20] M. J. Fuchter, J.-N. Levy, *Org. Lett*. **2008**, *10*, 4919-4922.

[21] P. Bonilla, Y. P. Rey, C. M. Holden. P. Melchiorre, *Angew. Chem. Int. Ed*. **2018**, *57*, 12819-12823.

[22] H. V. Thulasiram, R. M. Phan, S. B. Rivera, C. D. Poulter, *J. Org. Chem*. **2006**, *71*, 1739-1741.

[23] G.-S. Liu, Y.-Q. Zhang, Y.-A. Yuan, H. Xu, *J. Am. Chem. Soc*. **2013**, *135*, 3343-3346.

[24] J. C. Ewing, G. S. Ferguson, D. W. Moore, F. W. Schultz, D. W. Thompson, *J. Org. Chem*. **1985**, *50*, 2124-2128.
